# Supplementary material for: Mantis: flexible and consensus-driven genome annotation
Source: Gigascience. 2021 Jun 2;10(6):giab042. doi: 10.1093/gigascience/giab042 (PMC8170692; doi:10.1093/gigascience/giab042)
Supplement: giab042_GIGA-D-20-00320_Revision_1 [file giab042_giga-d-20-00320_revision_1.pdf]

GigaScience

Mantis: flexible and consensus-driven genome annotation

--Manuscript Draft--

**Manuscript Number:**	GIGA-D-20-00320R1
**Full Title:**	Mantis: flexible and consensus-driven genome annotation
**Article Type:**	Technical Note
**Funding Information:**	Fonds National de la Recherche Luxembourg (PRIDE17/11823097) — Dr Paul Wilmes
**Abstract:**	Background<br><br>The past decades have seen a rapid development of the (meta-)omics fields, producing an unprecedented amount of high-result ion and high-fidelity data. Through the use of these datasets we can infer the role of previously functionally unannotated proteins from single organisms and consortia. In this context, protein function annotation can be described as the identification of regions of interest (i.e., domains) in protein sequences and the assignment of biological functions. Despite the existence of numerous tools, some challenges remain, specifically in terms of speed, flexibility, and reproducibility. In the era of big data analysis, it is also increasingly important to cease limiting our findings to a single reference, coalescing knowledge from different data sources, and thus overcoming some limitations in overly relying on computationally generated data from single sources.<br><br>Results<br><br>We implemented a protein annotation tool - Mantis, which uses database identifiers intersection and text mining to integrate knowledge from multiple reference data sources into a single consensus-driven output. Mantis is flexible, allowing for the customization of reference data and execution parameters, and is reproducible across different research goals and user environments. We implemented a depth-first search algorithm for domain-specific annotation, which significantly improved annotation performance compared to sequence-wide annotation. The parallelized implementation of Mantis results in short runtimes while also outputting high coverage and high-quality protein function annotations.<br><br>Conclusions<br>Mantis is a protein function annotation tool that produces high-quality consensus-driven protein annotations. It is easy to set up, customize, and use, scaling from single genomes to large metagenomes.<br><br>Mantis is available under the MIT license available at https://github.com/PedroMTQ/mantis.
**Corresponding Author:**	Pedro Queirós<br>University of Luxembourg<br>Esch-sur-Alzette, LUXEMBOURG
**Corresponding Author Secondary Information:**	
**Corresponding Author's Institution:**	University of Luxembourg
**Corresponding Author's Secondary Institution:**	
**First Author:**	Pedro Queirós
**First Author Secondary Information:**	
**Order of Authors:**	Pedro Queirós

Powered by Editorial Manager® and ProduXion Manager® from Aries Systems Corporation

	Francesco Delogu
	Oskar Hickl
	Patrick May
	Paul Wilmes
**Order of Authors Secondary Information:**	
**Response to Reviewers:**	We have provided a PDF document entitled "reviewer_response.pdf" where we list all the editor and reviewer comments, alongside the respective response.<br>Regardless, we also provide the same documents below:<br><br>We would like to thank both the editor and the reviewers for the very in-depth review. Overall, we have tried to explain our methods better in the revised version of the manuscript by providing more examples and figures. We have also repeated our analyses due to the proposed (and some additional) changes in Mantis. To provide better explanations to our original study, we have divided each review into bullet points to address each comment separately. Each comment is marked as “RXCY”, X being the reviewer number and Y the comment number. R0 corresponds to the GigaScience editor comments, R1 to the reviewer Karen Ross and R2 to the reviewer Carlos Cantalapiedra.<br>In response to the reviewer comments, we have made some substantial changes in the manuscript, benchmarking and implementation of Mantis. Major changes are listed below:<br>•We have added a new reference data source - NCBI protein family models, from this link https://ncbiinsights.ncbi.nlm.nih.gov/tag/protein-family-model/. These are referred to as NPFM in the manuscript, and, alongside eggNOG, are also used for taxa-specific annotation;<br>•The original comparison between the reference annotations and the annotations produced by the several tools used text similarity analysis and intersection of IDs. The new benchmark now only uses the intersection of IDs, as we believe it to be less error-prone;<br>•The following new subsubsections have been added to the subsection “Initial quality control” in the section “Analysis”: “Impact of hit processing algorithms”, “Contribution of the different reference data sources”, “Impact of consensus generation”, and “Hit processing approximation”;<br>•The following sections have been added: “Annotating metagenomes”, “Computational efficiency” (moved from the supplements);<br>•The following figures have been added: “Figure 2. Homolog selection for the three hit processing algorithms in Mantis” and “Figure 7. Inter-HMMs hit processing steps”. Please keep in mind figure numbers may have changed from the past manuscript version;<br>•The old supplemental PDF has been removed. The new supplemental PDF includes the following sections: “Impact of the e-value threshold”, “Execution commands”, and “Consensus between free text functional descriptions”.<br><br>Some sections have also been rewritten and other minor changes were made. For easier comparison, with our resubmission, we include a PDF comparison of the old and revised manuscripts “version_comparison.pdf” and a version of the manuscript with lines “manuscript_lines.pdf”.<br>Whenever applicable, we also include transcripts of the manuscript in our response to the reviewers.<br><br>R0C1:<br>register any new software application in the bio.tools and SciCrunch.org databases to receive RRID (Research Resource Identification Initiative ID) and biotoolsID identifiers, and include these in your manuscript.<br>Response:<br>Mantis has been submitted to both databases, the corresponding IDs have been added to the section “Availability of source code and requirements”.

Powered by Editorial Manager® and ProduXion Manager® from Aries Systems Corporation

Reviewer #1

R1C1:
presentation of the method and its testing needs clarification to enable understanding of exactly how the tool works and to properly evaluate its performance. Providing specific examples in a few key places would help a lot.
Response:
We thank the reviewer for this important point. We have expanded the “Multiple predictions per protein” section, explaining how each score is calculated and how combinations of hits are generated. Please see the rewritten “Multiple predictions per protein” section.
In addition, we have also expanded the section “Using multiple reference datasets” and added a new figure (7) for inter-HMMs hit processing, where we explain each step and give specific examples.

R1C2:
Figures 5 and 6 make it clear that Mantis can find matches to multiple non-overlapping regions of the protein (multiple domain matches). It seems plausible that sometimes Mantis would identify some but not all of the domains in a manually annotated SwissProt entry or might identify some domains correctly but then also have one or more false positive domains. However, the calculation of %TP and %FP appears to be done at the whole protein level--"The % TPs and % FPs are, respectively, defined as the number of TPs and FPs divided by the number of sequences in the sample." How does this calculation take into account multiple matches per protein? An example here would help.
Response:
We thank the reviewer for this very valid comment. Domain-specific benchmarking comes with a set of issues that we cannot address since Mantis uses multiple reference sources. While we agree that this may result in a sub-optimal benchmarking, we believe this compromise to be the only feasible benchmarking solution. However, please also see the new section “Impact of consensus generation” where we also tested the performance of Mantis with the BPO algorithm and without consensus generation (just one prediction per sequence) and showed that even without multiple predictions and multiple references per prediction, the BPO still achieves an F1 score of 0.7627. These results showcase the importance of having a broader pool of hits (due to the use of multiple references) when choosing the best predictions. As the reviewer mentioned, it is possible that Mantis may annotate more domains than it should. For example, the protein entry A8D8P8 (https://www.uniprot.org/uniprot/A8D8P8) contains two domains: Piwi (http://pfam.xfam.org/family/PF02171) and PAZ (https://pfam.xfam.org/family/PF02170). Mantis could theoretically annotate both these domains and an additional domain in a domain-centric benchmark. We could then consider that Mantis annotated 2 TPs and 1 FP. In the current whole-sequence-centric benchmark this will simply be considered as 1 TP. While the domain-centric approach may seem more plausible, Mantis does not only use the domain-specific Pfam database, instead, it relies on multiple reference sources, most of which have different scopes and wildly different resolutions. Some reference sources focus on whole sequence annotation (i.e. eggNOG), others aim at providing the general functional scope of the protein (i.e. TIGRFAM), and others domain-specific resolution (i.e. Pfam). Most of these references (besides Pfam) have little to no information on domains. Even if domain-specific information were available, we found that Swiss-Prot domain information mostly cross-links with Pfam, which would invalidate the benchmark for sequences annotated with other reference sources (and other tools). With such different resources, domain-centric benchmarking becomes extremely complex (if feasible at all). On the other hand, Swiss-Prot provides comprehensive cross-linking for whole-sequence annotations, which better fits the wide ranging scopes of the reference sources used by Mantis. It also allows for a fairer benchmark against other tools. To conclude, while we do agree with the reviewer, for practical reasons, we have decided whole-sequence benchmarking to be the only option (even if a sub-optimal one). We have also clarified this issue in the manuscript, please see the following transcript in page 11, lines 949-968, in the subsection ”Methods.Establishing a test environment”:
This has some significant limitations: (i) the functional description is the same but the corresponding set of identifiers is not; and (ii) when annotating multiple regions of the

Powered by Editorial Manager® and ProduXion Manager® from Aries Systems Corporation

protein (which is the case when using Mantis' DFS and heuristic algorithms), it is possible that only one of the annotated regions has IDs that intersect with the respective sequence reference annotation. Unfortunately, due to the different resolutions of the reference HMMs, it is not always possible to understand whether an annotation refers to a specific domain or a partial whole-sequence hit. While a domain-centric benchmark would be feasible for Pfam, the same is not true for the remaining reference HMMs with broader resolutions (e.g., TIGRFams provides general functional annotations). Despite these limitations, since whole-sequence reference annotations contain comprehensive cross-linking with other databases, it provides clear benefits: (i) it better fits the wide-ranging scopes of the reference data sources, and (ii) allows for a more fair benchmark of the different PFA tools that may use different reference data sources (and thus output annotations with different database IDs).

R1C3:
When creating the consensus annotation, Mantis evaluates whether the annotations from two different resources are similar. First, it is confusing that the authors use the word "complementary" to describe the situation where annotations from two resources are similar. "Consistent" or "confirmatory" might be better word choices. Second, it is unclear what the outcome would be if two resources find hits on two non-overlapping regions. These hits would obvious be very dissimilar in annotation. Would they both be included in the consensus annotation?
Response:
We acknowledge the reviewer’s comment. We used the term complementary since cross-linking can add new identifiers to the final annotation. Nevertheless, we changed the term “complementary” to “consistent” whenever applicable. To answer the second question, yes, both hits would be kept; an example is now available in the inter-HMMS figure 7.

R1C4:
In the "Establishing a test environment" sub-section of the Methods, it would be helpful to clarify what is meant by "reference annotation". Where does it come from and what exactly does it consist of?
Response:
The reference annotations come from Uniprot, it has now been specified in the "Establishing a test environment" section what exactly they contain and where they come from. Please see the following transcript in page 10, lines 927-942 in the subsection ”Methods.Establishing a test environment”:
For annotation quality benchmarking, we evaluate each annotation produced by Mantis and check whether it agrees (database IDs intersection) with the respective reference annotation, creating a confusion matrix. We created two main types of test samples, the first consisting exclusively of curated UniProt protein entries (and the respective annotations) which were then split by date of creation (2010-2020, 2015-2020, 2018-2020, 2020). The second type consisting of organism-specific UniProt protein entries, with a mix of curated and automatically generated annotations. Each sequence's reference annotation consists of the UniProt protein function annotations.
Each sequence reference annotation and the respective PFA tool's annotation is composed of a set of identifiers (if available: enzyme ECs, Gene ontology (GO) IDs, eggNOG IDs, KEGG orthology IDs, Pfam IDs, and TIGRfam IDs) and functional descriptions.

R1C5:
Also, this section says that Mantis annotations that don't match the reference are considered false positives, but it also says that when Mantis outputs "valid annotation but the reference is either of poor quality or non-existent" that this is considered a potential new annotation. How exactly is the distinction made between a false positive and potential new annotation?
Response:
A potentially new annotation (PNA) is a protein sequence that has been annotated with a protein function annotation tool but that does not have a proper reference annotation (inexistent or of poor quality - e.g. “hypothethical protein”). We do not consider these to be FPs because Uniprot also contains automatically generated annotations. For this reason, potentially new annotations mostly occur when we annotated the several organism samples, which may contain a large amount of automatically annotated sequences or un-annotated sequences. These PNAs are excluded from the confusion

Powered by Editorial Manager® and ProduXion Manager® from Aries Systems Corporation

	matrix but are included in the annotation coverage calculation. Please see the following transcript in page 11, lines 972-996 in the section ”Methods.Establishing a test environment”:<br>True-Positives (TP) occur when the PFA tool generated annotation and the reference annotation share one or more database IDs (e.g., Pfam ID), False-Positives (FP) when no database IDs are shared. False-Negatives (FN) when the PFA tool does not annotate a protein sequence, but a reference annotation is available, and True-Negatives (TN) when the PFA tool does not annotate a protein sequence, and no reference annotation is available. The functional text descriptions are not taken into account during the benchmark, therefore if an annotation has no identifiers, we simply consider there is no annotation. As such, it is possible that no reference annotation exists, but the PFA tool manages to annotate the sequence; when this is the case this is classified as potentially new annotation (PNA).<br>Protein sequences annotated with the descriptions “unknown function”, “uncharacterized protein”, ”hypothetical protein” or with Pfam’s "domain-unknown-function"/DUF IDs are not taken into account during benchmarking (for reference and PFA tools annotations). In addition, it is also possible that the reference contains no annotation at all. Should the PFA annotate the sequence, both these cases would correspond to PNAs.<br>Since no ground-truth exists in these scenarios, PNAs are excluded from the confusion matrix classes (not used during any performance metrics) and are only used to calculate the annotation coverage. PNAs can potentially provide novel insight into protein sequences without any previous annotation.<br><br>R1C6:<br>When calculating the similarity threshold (supplemental material), the authors analyzed a group of proteins where the identifiers disagreed. Were these cases where the Mantis annotation of a protein sequence had no identifiers in common with the Swiss-Prot annotation of the same sequence? Or was the Mantis-annotated sequence compared with a different sequence in Swiss-Prot that had no identifiers in common? If the sequences were the same, then why did they have no identifiers in common? A specific example would be helpful here.<br>Response:<br>As both reviewers pointed out (please see R2C23), the similarity threshold setting was unclear and was therefore removed entirely from the supplements. In addition, the method used to calculate the similarity between functional descriptions has been moved to the supplements, as we think it does not entirely fit the context of the manuscript. Please see the section “Consensus between free text functional descriptions” in the supplements.<br>The threshold benchmark was completely removed; instead, the similarity threshold was determined by empirically evaluating a set of 1000 pairwise descriptions. Documents above the threshold of 0.9 (0-1) were considered to be describing the same function.<br><br>R1C7:<br>How much does the text mining analysis of the free text descriptions contribute to the similarity score? Is the similarity dominated by identifier matching or does the free text analysis contribute significantly?<br>Response:<br>We analysed the contribution of both methods for the UniProt sample (2010-2020, and found that the free text analysis contributes roughly 35%.<br>Since one could argue that Mantis’ performance is simply due to the multiple domains algorithms and consensus generation, we also performed additional tests: (i) Mantis default execution with the DFS algorithm; (ii) Mantis without text analysis with the DFS algorithm; (iii) Mantis without text analysis and identifiers intersection with DFS algorithm; (iv) Mantis default execution with the BPO algorithm; (v) Mantis without text analysis with the BPO algorithm; (vi) Mantis without text analysis and identifiers intersection with BPO algorithm.<br>Condition iii refers to a protein annotation methodology without consensus generation, but that uses multiple reference datasets and outputs multiple annotations per sequence.<br>Condition vi refers to the baseline protein annotation methodology with the added benefit of using multiple reference datasets.<br>In essence, each test will check each feature of Mantis individually: inclusion of

Powered by Editorial Manager® and ProduXion Manager® from Aries Systems Corporation

different domains - DFS/BPO; consensus generation exclusively with IDs intersection; and no consensus generation (without IDs intersection and without text analysis). We calculated the F1 score of each condition, (i) had a F1 score of 0.8268, (ii) 0.7902, (iii) 0.7740, (iv) 0.8138, (v) 0.7789, and (vi) 0.7627. We can see that all conditions had "close" F1 scores indicating that Mantis' superior performance is not caused by just blindly adding more information. We can also see the additive effect of the algorithm and the consensus generation steps. We then also checked these results against eggNOG-mapper where a F1 score of 0.7027 was obtained. Put together, these results suggest that simply using multiple reference datasets (condition vi) already boosts the F1 score past eggNOG-mapper; adding the extra Mantis features (hit processing algorithms and consensus generation) boosts performance even further.
We have added this information to the manuscript in the new subsubsection "Impact of consensus generation" in the subsection "Initial quality control" in the section "Analysis", page 5, lines 346-368.
Please refer to supplemental table 4 for further details.

R1C8:
Although this information is in the Methods, it would be helpful to define briefly in the Results section how precision is being calculated. It would be useful to include the explanation of why precision is considered more important than recall in the Results as well.
Response:
While in general we do consider precision more important than recall (since recall tends to be high anyway), we now measure performance with the F1 score (harmonic mean of precision and recall). We think this metric will overall be more informative. As suggested, we have also added how each metric is being calculated in the Analysis introduction. Please see the following transcript in page 4, lines 261-275 in the section "Analysis":
In order to compare the performance between the different tests, we calculated a confusion matrix for each test. For future reference, a True-Positive (TP) occurs when a functional annotation (predicted from a PFA tool) shares one or more database IDs with the respective reference annotation (e.g., Pfam ID); a False-Positive (FP) when no database IDs are shared; a False-Negative (FN) when the PFA tool does not annotate a protein sequence but a reference annotation is available; and a True-Negative (TN) when the PFA tool does not annotate a protein sequence and no reference annotation is available. Precision is defined as TP/(TP+FP) , Recall as TP/(TP+FN), and F1 score(harmonic mean of precision and recall) as 2 × (Precision*Recall)/(Precision+Recall). The F1 score is used as a performance metric. Further details on the benchmark are available
in "Establishing a test environment"

R1C9:
It would be useful to move figure 5 earlier in the paper when the hit processing algorithms are first introduced.
Response:
Figure was edited to provide further details on the hit processing algorithms and was moved. It is now figure 2.

R1C10:
In the Background section, annotation iii could be removed from the first two examples of glucose degradation annotation because it isn't used to make the authors' point.
Response:
We removed the annotation iii as the reviewer suggested.

Reviewer #2

R2C1:
how several steps of the Mantis annotation algorithm actual work (specifically those related with inter-HMM and consensus-driven integration),
Response:

Powered by Editorial Manager® and ProduXion Manager® from Aries Systems Corporation

	We would like to thank the reviewer for the very in-depth review. We hope we have provided clarity in some of the more complex steps. Please also see R1C1 in relation to this point.<br><br>R2C2:<br>First, the list of keywords maybe should be reviewed: function as a keyword it is too generic in my opinion. protein; function; annotation could be merged into protein function annotation. Also, I could not find an explanation of what NLP means. Please, review also the other keywords included. The same for the list of abbreviations. For example, I could not find NLP there, but I found that BPO is also not included. Please, review all abbreviations both from the main text and from the list.<br>Response:<br>We thank the reviewer for this important point. We have reviewed the keywords and abbreviations as suggested. The keywords were changed to “bioinformatics; consensus; homology; hmm; protein function annotation”.<br><br>R2C3:<br>Please, review also the text for potential errors. For example, "whilst sing" --> "whilst using", or "herein after" --> "hereinafter". I am also not native English, so my review in this sense could be not the best, so maybe a professional language review could be good to improve the manuscript. I leave this decision to the authors, editor and maybe some Ensligh-native speaker from among the reviewers.<br>Response:<br>We would like to thank the reviewer for this comment; indeed, while the authors and a native speaker had previously reviewed the paper, some errors escaped our attention. Grammarly was also used for revision. We redoubled our efforts on this end, carefully reviewing the manuscript for typos/errors.<br><br>R2C4:<br>Regarding supplemental material, it has been a bit confusing for me. There are links throughout the text, but there are also other supplemental files at the end of the manuscript. Are all of them valid? Nomenclature of sections, tables, figures, etc from the different materials should be merged, and all supplemental material provided in a common way. This is what I had:<br>Throughout the text Supplemental Table 1 is a link to<br>https://htmlpreview.github.io/?https://raw.githubusercontent.com/wiki/PedroMTQ/mantis/Resources/tab_e_value.html<br>Supplemental Table 2 is a link to<br>https://htmlpreview.github.io/?https://raw.githubusercontent.com/wiki/PedroMTQ/mantis/Resources/tab_uniprot_algorithms.html<br>Supplemental Table 3 is a link to<br>https://htmlpreview.github.io/?https://raw.githubusercontent.com/wiki/PedroMTQ/mantis/Resources/tab_genomes.html<br><br>At the end of the manuscript<br>Supplements.pdf<br>Defining a similarity threshold (Figure 1)<br>Annotating metagenomes<br>0.1 Benchmarking annotation efficiency (Figure 2, Table 1, Table 2)<br>Supplementary benchmarking against other PFA tools (Table 3, Table 4)<br>Supplemental tables (Table 5, Table 6, Table 7, Table 8)<br>References<br>Supplements.xlsx<br>Mantis vs emapper<br>DFS vs BPO vs heuristic<br>Comparisons with Prokka, DeepEC, emapper, RAST<br>Response:<br>Previous links were used as a way to make the manuscript more “interactive”, and had the same data found in the supplements. Thank you for the suggestion. We have now moved all the tables into a single excel file. All the tables have been numbered and a table of contents has been included in the first spreadsheet.<br><br>R2C5:<br>Some comments about the abstract. What do the authors mean with "adaptable"? How

is this different from "flexible"? Also, I would say that "reproducible" is implicit of computational tools, in general terms. Why are the authors stressing this in the abstract as a feature specifically of Mantis? Also, the authors say that "Mantis is fast, annotating an average genome in 25-40 minutes", which I would like better "an average bacterial genome", for instance.
Response:
We used the adjective “flexible” as a means to say that Mantis can be deeply customized, whereas “adaptable” would be in regards to using different reference HMMs, so that it can be used in niche environments (for example). However, since they can be viewed as synonyms, we standardized nomenclature to only include “flexible”. We agree that reproducibility should be implicit in every bioinformatics software, but unfortunately, it still is not. Therefore, we kept reproducibility as a feature, because we think reproducibility remains a relevant topic in bioinformatics. In fact, dedicated tools were created to address this exact issue (e.g. Snakemake - https://doi.org/10.1093/bioinformatics/bts480), in addition, a paper from Mangul et al. (2019 - https://doi.org/10.1371/journal.pbio.3000333) also highlights that reproducibility remains a concern. We removed the execution time (since it depends on hardware and data at hand), opting only to mention that it is fast due to parallelization.

R2C6
And some comments about the Background section. I personally would remove the first 2 sentences, but this is completely personal preference. Also, I am not sure to agree with the protein function annotation definition which is given both in the abstract and in Background: "protein function annotation(PFA), which is the identification of regions of interest (domains) in a sequence and assignment of biological function(s) to these regions."
Response:
We understand there is no universal definition for protein function annotation. From the many available definitions, we give the one that is most appropriate for the task for which we have designed Mantis. Therefore, we now clearly state that this definition is our understanding of the task and the challenge. We changed the paper accordingly to reflect that this is only how we, the authors, define the problem we aim to address with Mantis.
Please see the following transcript in page 2 lines 21-25 in the section “Background”:
One of the ways to make sense of this data is through protein function annotation (PFA), which is, in the context of this paper, the identification of regions of interest (i.e., domains) in a sequence and assignment of biological function(s) to these regions.

R2C7:
Later, the authors say that: "We reviewed the implementation of three widely used PFA tools [13, 24, 14] and observed that the processing of candidate annotations (i.e. sequences or HMM profiles which are highly similar to the query sequence) is done by capturing only the most significant candidate between the references ("best prediction only", herein after called BPO)". That is: Prokka, eggNOG-mapper and InterProScan 5. And, "This classic PFA approach works well for single-domain proteins, but multi-domain proteins may have multiple putative predictions [30, 31, 32], whose location in the sequence may or may not overlap.".

The previous 2 sentences, although are true to some extent, are in my opinion an oversimplification. For example, stating that eggNOG-mapper picks the most significant candidate between the references is true, but only part of the story. eggNOG-mapper picks the most significant homolog as seed to retrieve orthologs from which annotations are
transferred. Therefore, a protein can be annotated with multiple domains which may or may not overlap, which I believe is true also for Prokka and InterProScan 5. Therefore, although I think that the authors have a valid point here, I believe that they should focus on the advantages of identification of multiple references, but not as a mandatory requirement for multi-domain annotation. Actually, the 3 "reviewed" tools are very different, and if they are mentioned specifically as reviewed, a more precise description of their approaches, weaknesses or features which could be improved would be expected.
Response:
From what we can tell, Prokka does return the best hit only, as seen in the commands

Powered by Editorial Manager® and ProduXion Manager® from Aries Systems Corporation

below (from Prokka's GitHub -
https://github.com/tseemann/prokka/blob/master/bin/prokka):

Line 69:

```
my $BLASTPCMD = "blastp -query - -db %d -evalue %e -qcov_hsp_perc %c -
num_threads 1 -num_descriptions 1 -num_alignments 1 -seg no";
```

In addition, it only returns one hit per sequence as seen in the code below:

Line 1061:

```
for my $db (@database) {
  # skip HMMs if --fast mode
  if ($fast && $db->{FMT} !~ m/blast/i) {
    msg("In --fast mode so skipping non-BLAST search against ".$db->{DB});
    next;
  }
  # create a unqiue output name so we can save them in --debug mode
  my $outname = "$prefix.".basename($db->{DB}).".tmp.$$";
  # we write out all the CDS which haven't been annotated yet and then search them
  my $faa_name = "$outdir/$outname.faa";
  open my $faa, '>', $faa_name;

  my %cds;
  my $count=0;
  for my $sid (@seq) {
    for my $f (@{ $seq{$sid}{FEATURE} }) {
next unless $f->primary_tag eq 'CDS';
next if $f->has_tag('product');
$cds{++$count} = $f;
print $faa ">$count\n",
        $f->seq->translate(-codontable_id=>$gcode, -complete=>1)->seq,"\n";
    }
  }
  close $faa;
```

As the reviewer suggested, eggNOG-mapper is indeed somewhat more complex, and it is relevant not to oversimplify.

Since it is not in the context of the manuscript to extensively review other tools, we changed that particular section of the manuscript. Please see the following transcript in page 2 lines 50-59 in the section "Background":

The most common method of processing candidate annotations (i.e., sequences or HMM profiles that are highly similar to the query sequence) is done by capturing only the most significant candidate ("best prediction only", hereinafter called the BPO algorithm). This PFA approach works well for single-domain proteins, but multi-domain proteins may have multiple putative predictions, whose location in the sequence may or may not overlap. This selection criterion may potentially lead to missing annotations and is therefore not suitable in complex PFA scenarios.

R2C8:

Then, regarding consensus integration of annotations the authors say that "This approach addresses three very relevant issues with PFA [34, 35, 51, 52]: over-annotation (through the use of overlapping but independent sources, thus obtaining a more reliable final annotation); under-annotation (through the use of multiple reference sources, which implicitly leads to a wider search space); and elimination of redundancy (through the creation of a consensus-driven annotation)." I agree that this approach would deal with under-annotation and with elimination of redundancy, due to integration of annotations from different sources. However, I fail to understand how this approach helps with over-annotation.

Response:

We separated redundancy and over-annotation as we think these are different issues. Redundancy is here simply the duplication of data (e.g. having the same identifier multiple times) which can be solved by making sure the annotation metadata (i.e. identifiers and text description) is not repeated. Over-annotation is more complex, and it pertains to the fact that if we use multiple reference datasets, we will most likely end up with annotations pointing to different functions. This is clearly a problem, and so this is something we address with the consensus generation; we do so by analysing all the functional annotations from the multiple independent reference datasets and checking which ones point towards the same function. If independent references point towards

Powered by Editorial Manager® and ProduXion Manager® from Aries Systems Corporation

	the same function, we consider it more likely for this function to be the “real” function of the respective protein sequence. This is, as we see it, one of the main benefits of using multiple references.<br>However, we agree that this was not clear in the initial manuscript, therefore please see the following transcript with the modified text in page 3 lines 178-189 in the section “Background”:<br>Under-annotation is addressed through the use of multiple reference data sources, which implicitly leads to a wider search space. Over-annotation is minimized through the generation of a consensus-driven annotation, which identifies and merges annotations that are consistent (i.e., similar function) with each other (e.g., if three out of five independent sources point towards the same function and two others point towards other, unrelated functions, then these three annotations are more likely to be valid), and eliminating the remaining inconsistent annotations. Redundancy is eliminated by removing duplicate database IDs and/or extremely similar descriptions.<br><br>R2C9:<br>First, regarding reference data, the authors say that eggNOG OGs are from eggNOG 5. However, actually TSHMMs and annotations downloaded by Mantis correspond to eggNOG 5 data, but the unspecific HMMs are from eggNOG 4.5. Checking some lines of code:<br><br>./source/MANTIS_DB.py: eggnog_downloads_page_hmm = 'http://eggnogdb.embl.de/download/eggnog_4.5/data/NOG/NOG.hmm.tar.gz'<br>./source/MANTIS_DB.py: eggnog_downloads_page_annot = 'http://eggnogdb.embl.de/download/eggnog_4.5/data/NOG/NOG.annotations.tsv.gz'<br>./source/MANTIS_DB.py: eggnog_downloads_page = 'http://eggnog5.embl.de/download/latest/per_tax_level/'+str(taxon_id)+'/'<br>./source/MANTIS_DB.py: url = 'http://eggnogdb.embl.de/download/emapperdb-5.0.0/eggnog.db.gz'<br><br>This is important, and it will impact both the use of the tool by users and the benchmarks presented in the paper. Note that eggNOG 4.5 and eggNOG 5 orthologous groups are not cross-linked, and therefore annotations for unspecific NOGs will be lacking in Mantis output. For example, I annotated with Mantis a protein using unspecific NOGs as ENOG4111x9y. This is an OG from eggNOG 4.5, as expected, and I got only the description as annotation ("uncharacterized protein yaho"), when I should be getting also annotation identifiers, GO terms for example. The equivalent OG in eggNOG 5 is ENOG502DN1T though. This can be checked comparing OGs from http://eggnog45.embl.de/ and http://eggnog5.embl.de/<br>Response:<br>Thank you for this comment, we initially used the data from eggNOG 4.5 as it contained a pre-compiled general HMM. As the reviewer mentioned, this will have an impact on the produced annotations, for this reason the general eggNOG HMM is now a compilation of all the non-redundant TSHMMs from the following NCBI IDs: 2157 (Archaea), 2 (Bacteria), 2759 (Eukaryota), 10239 (Viruses), 28384 (Others), and 12908 (Unclassified). These IDs correspond to NCBI’s top level taxonomy rank IDs. We have also added another set of TSHMMs from NCBI.<br>We have added this information to the manuscript, please see the following transcript in page 8 lines 704-711 in the subsection “Methods.Reference data and customization”:<br><br>A general NPFM HMM was created by pooling all non-assigned HMM profiles and the TSHMMS from the following NCBI IDs: 2157 (Archaea), 2 (Bacteria), 2759 (Eukaryota), 10239 (Viruses), 28384 (Others), and 12908 (Unclassified). These IDs correspond to NCBI’s top level taxonomy rank IDs. A general eggNOG HMM was created by pooling together the TSHMMs from the aforementioned taxon IDs.<br><br>R2C10:<br>Besides that, "TSHMMs metadata was extracted from the eggNOG SQL database" I would ask authors to specify that this is an eggNOG-mapper DB, not an eggNOG one, as can be seen in the link above.<br>Response:<br>This has now been rectified in the manuscript.

Powered by Editorial Manager® and ProduXion Manager® from Aries Systems Corporation

R2C11:
Also, the TSHMMs databases are huge as a whole. In my opinion, including in Mantis an option to download only TSHMMs from a specific taxon specific taxa would be great and almost mandatory for the "flexibility"
Response:
Thank you for the suggestion, this feature was implemented and can now be used by providing a list of NCBI IDs or organism names in the MANTIS.config file (with the line “nog_tax”). If an organism name is introduced, an automatic web search retrieves the respective NCBI ID. A lineage for each NCBI ID is then generated and all TSHMMs are downloaded and compiled. This is now mentioned in the Mantis’ wiki.

R2C12:
i) sample pre-processing, where samples are split in chunks for parallelization. Also, input data should be proteins. Are CDS queries (fasta in nucleotide/DNA format) also accepted?
Response:
No, Mantis doesn’t do gene calling or translation, we opted for this approach to eliminate the need to integrate further third party bioinformatic tools which often depend on the use-case and/or user preference.

R2C13:
"HMMER outputs a domtblout le [23], where each line corresponds to a hit/match between the reference dataset and the unknown protein sequence. The e-value within the HMMER command limits the available solution space to be analyzed in the posterior processing steps." Which is the e-value used by Mantis then? The e-value from "the HMMER command" or the e-value from the "domtblout" file? Note that e-value in "domtblout" is the "e-value of the overall sequence/profile comparison (including all domains)", which outputs also other values, like "c-evalue" and "i-evalue". (http://eddylab.org/software/hmmer/Userguide.pdf, pp70-71, see also pp 34-35). Therefore, if using the "e-value", either all the hits from such domain should be included for intra-HMM integration, or preferably some hits should be discarded using the "i-evalue", for example. Also note that as the score used to compare combinations through DFS includes e-value, if several repeated domains exist in a sequence maybe the i-evalue should be included in the score instead of the overall e-value several times, which would inflate the combination in which several domains are part of the sequence.
Response:
Regarding the sentence:
Which is the e-value used by Mantis then? The e-value from "the HMMER command" or the e-value from the "domtblout" file?
We are not sure what the reviewer means here, the e-value from the HMMER command is to set the threshold, the actual e-value that is extracted (and used by Mantis) is the one that is reported in the domtblout file.
As the reviewer noted the DFS and heuristic algorithms should use the independent e-value - column 12 (which they did and do), whereas the BPO should use the full sequence e-value - column 6 (it didn’t before). The full sequence e-value is used by the BPO algorithm since we are interested in only the best prediction for a certain protein sequence.
We have also now specified in HMMER’s execution to use the “--domE” threshold when using the DFS and BPO algorithm, and use the “-E” threshold when using the BPO algorithm.
We have now specified this in the paper, please see the following transcript from page 9, lines 758-763 in the subsection “Multiple hits per protein” in the section “Methods”:
Mantis uses HMMER's independent e-value when using the DFS and heuristic algorithms; whereas it uses the full sequence e-value when using the BPO algorithm (since only the best hit is extracted per protein sequence). For simplicity purposes, both are simply referred to as e-value throughout this paper.
And from the supplements section “Execution commands”:
When using the DFS or heuristic algorithm, Mantis uses HMMER's independent e-value as a confidence score, and, when using the BPO algorithm, it uses the full sequence e-value (see http://eddylab.org/software/hmmer/Userguide.pdf pp.71-72). The e-value threshold set in the hmmsearch will be the same for all algorithms, however, when running Mantis with the DFS or heuristic, the threshold variable will be -domE, whereas for BPO, it will be -E. The e-value type and threshold chosen reflect

Powered by Editorial Manager® and ProduXion Manager® from Aries Systems Corporation

	the fact that the DFS and heuristic algorithm may capture multiple hits per protein, whereas the BPO only captures the one hit per sequence.<br><br>R2C14:<br>"should the DFS algorithm running time exceed 60 seconds, Mantis employs the previously described "heuristic" algorithm [30], which scales linearly and outputs an unique combination of hits." How many queries in average could be affected by this? Is the user warned somehow if some results are produced with the heuristic algorithm due to this DFS time limit?<br>Response:<br>Yes, the user is warned if the DFS algorithm exceeds the time limit, this is now specified in the manuscript. The user can also customize the time limit and/or choose which algorithms to use (BPO, heuristic or DFS). We calculated how many times the heuristic algorithm was used as a backup during the hit processing of the Swiss-Prot sample (2010-2020). We found that for the intra-HMMs hit processing, the heuristic algorithm was used in roughly 7.2% of the sequences, and for the inter-HMMs hit processing in 0.5% of the sequences. This information has been added to the manuscript in the section “Hit processing approximation”.<br><br>R2C15:<br>Are "output_annotation.tsv" files a product of this 3rd step?<br>iv) metadata integration. Annotation description and identifiers are added to the respective hits. I guess this yields the "integrated_annotation.tsv" output files.<br>Response:<br>The "output_annotation.tsv" is a byproduct of the intra-HMM hit processing step, which is specific to each reference. However, to have all predictions in one file, we merge all hits from the different references into one file. The "integrated_annotation.tsv" is fundamentally the "output_annotation.tsv" but with metadata. The "consensus_annotation.tsv" contains, for each reference, a consensus annotation from the "integrated_annotation.tsv", meaning that if, for example, a protein sequence has five hits in the "integrated_annotation.tsv" file and three of those are consistent (either through identifiers or functional text description) then only those three are included in the "consensus_annotation.tsv", the remaining twp are rejected. Of course, the user may still be interested in the rejected hits, which is why we opted to also provide the "integrated_annotation.tsv" file.<br>We have now specified in which step each output file is generated. Please see the following transcript in page 8 lines 675-690 in the subsection “Methods.Input and output”:<br>Mantis outputs, for each sample, three tab-separated files, each corresponding to a different step in Mantis' workflow: (i) a raw output output_annotation.tsv, with all the hits, their e-value, and coordinates; (ii) integrated_annotation.tsv, with the same information as output_annotation.tsv, but also with hits metadata (e.g., KEGG orthology IDs (KO), enzyme commission (EC) numbers, free-text functional description, etc); (iii) the main output file consensus_annotation.tsv, with each query protein ID and their respective consensus annotation from the different reference data sources (e.g., Pfam). These files provide contextualized output in a format that is both human and machine-readable.<br>A Mantis.out file is also provided per sample, serving as a log file for each execution step.<br><br>R2C16:<br>v) inter-HMM reference hits processing.<br>I have many doubts with this step. First, in "Mantis" section it is said that "During inter-HMM hits processing the DFS algorithm is again used to generate all the combinations of hits for all HMM sources." However, in "Methods - Using multiple reference datasets" this is not confirmed not further explained.<br>Response:<br>We added a new figure (7) for inter-HMMs hit processing in the section “Using multiple reference datasets”, where we explain each step and give specific examples. Succinctly, here we explain how the DFS algorithm is used to generate the combinations of hits (similar to the inter-HMM hits processing but instead of generating reference-specific combinations we pool all hits and generate combinations from that merged pool). We have extensively changed the “Multiple predictions per protein” subsection, so please refer to that section. We hope the explanation is now clear.

Powered by Editorial Manager® and ProduXion Manager® from Aries Systems Corporation

	R2C17:<br>Also, if DFS is used here, how are the e-values from the different references used to compute the scores. Because e-values are depend on database size, in contrast with bit-scores.<br>Response:<br>Thank you for the remark regarding e-value, however we already took this into account when implementing Mantis. Quoting page 33 of HMMER's user guide (http://eddylab.org/software/hmmer/Userguide.pdf):<br>"The E-value is based on the sequence bit score, the second number.This is the log-odds score for the complete sequence. Some people like to see a bit score instead of an E-value, because the bit score doesn't depend on the size of the sequence database, only on the profile and the target sequence. The E-value does depend on the size of the database you search: if you search a database ten times larger,you get ten times the number of false positives"<br>And again quoting the same user guide, page 70:<br>"Well, first of all,they still wouldn't be identical, because the target database size used for E-value calculations is different (number of target sequences for hmmsearch, number of target profiles for hmmscan, and it's good not to forget this."<br>As implied by the quotes, when using hmmsearch, the reference database size does not matter, the e-value is only scaled to the number of sequences in the target sample (e.g. the same sequence in a sample of 1 sequence will have double the e-value if it was instead present in a sample of 2 sequences, however the same sequence searched against a database with 10 or 5000 HMMs will have the same e-value). Hmmscan is scaled to database size and so the e-value will now be independent of sample size but dependent on database size.<br>Since, during homologs search, Mantis uses hmmsearch and the same sample for different references, then the e-value can be safely used. There are two exceptions to this: (i) when Mantis splits the sample into chunks, and (ii) when using TSHMMs (since in each taxon we only annotate the protein sequences which have not been annotated in the previous taxons). In both cases, the e-values are adjusted to the original sample size by storing the original file sample size in a separate intermediate file.<br><br>R2C18:<br>Also, query coverage will be very different by the very nature of the HMM profiles of the different databases. For example, PFAM is a database of domains, whereas eggNOG contains profiles which usually span a large section of the protein sequence.<br>Response:<br>This is true, which is why we use a composite combination score. Query coverage is taken into account, yes but so is the e-value. In this manner (and since we scaled the e-value), small but highly significant hits are also taken into account when other hits are large but with poor significance. This is, of course, a compromise, but it does permit for a fairer use of both larger (i.e. eggNOG) and smaller (i.e. Pfam) hmm profiles.<br>Also to note that since Pfam is made up of domains, if, for example, a protein sequence has 2 Pfam hits and 1 eggNOG hit, then it is likely that these 2 Pfam hits will be contained within the eggNOG hit. During consensus generation, should these share identifiers or have similar functional descriptions, the Pfam hits would also be included (this is actually one of the benefits of the consensus-driven annotation). While we do agree that this is not a perfect solution, to our knowledge, it is one of the most viable approaches when dynamically integrating references that are heterogeneous in composition and scale.<br><br>R2C19:<br>Moreover, it said later that "Since several groups of consensus annotations may be generated, we evaluate their quality and select the best one, considering the following: percentage of the sequence covered by the hits in the consensus, the significance of the hits (e-value) in the consensus, significance of the reference datasets (customizable), and the number of different reference datasets in the consensus."<br>"Since some sources are more specific than others, the user may also customize the weight given to each source during consensus generation [73]." (Methods - Reference data and customization)<br>Does this have to do with the DFS algorithm for inter-HMM integration? Also, how is the weighting of different references used? In the GitHub it is explained that it is a [0-1]

Powered by Editorial Manager® and ProduXion Manager® from Aries Systems Corporation

value, but, is it just a priority value or is it included in the DFS scoring formula somehow? I fail to find an explanation about how this weighting is used at all.
Response:
The scoring variables for inter and intra-HMM hit processing are now fully explained in the manuscript, which includes the weight values of each reference. The default weight values were defined according to the level of curation and specificity of the reference sources but can be changed by the user. These weights are only used during inter-HMMs hit processing since it is only then that we compare different reference sources. As the reviewer mentioned, yes it is only used during the DFS scoring formula (since the other algorithms will only generate one solution with one - BPO or multiple hits - heuristic).
Please see the following transcript in page 10 lines 892-904 in the subsection “Using multiple reference data sources” in the section “Methods” for the changes in the inter-HMMs hit processing:
HMM reference weight (HMMW) - average weight of all the reference data sources within the combination. This is calculated by adding all hits' hmm weights and dividing this sum by the number of hits in the combination (e.g., if a hit comes from Pfam, that has a weight of 1, and another from eggNOG, that has a weight of 0.8, HMMW would equal to (0.9+0.8)/{2 = 0.85). The default weight for each default reference data source has been set according to the authors' perception of the reference quality - creation method, curation level, and annotation completeness (eggNOG - 0.8, Pfam - 0.9, NPFM - 0.7, and TIGRfam - 0.5). This weight is customizable, the default weight for custom reference data is 0.7 (which can also be customized).

R2C20:
vi) Consensus generation
From Background section: "However, to our knowledge, there is no tool for the dynamic generation of a consensus from multiple protein annotations." "We implemented a two-fold approach to build a consensus annotation, first by checking for any intersecting annotation identifiers and second by evaluating how similar the free-text annotation descriptions are." From the "Mantis" section: "all the combinations of hits are expanded and intersected (if possible), the best consensus combination of hits is then selected for each query sequence."

So the tool is "checking" and/or "evaluating" intersections of identifiers and similarity of descriptions. But I fail to understand what is the desired or expected output after the check is done. According to the text, one would expect to obtain the intersection of the identifiers, which actually does not make sense at all, since many identifiers would be discarded just because they are not part of one or the other reference. The same question regarding descriptions: several descriptions are compared in a pairwise manner and those pairs with similarity are kept and those without any similar description are discarded?
Response:
We have rephrased that specific part of the section Mantis, as it was somewhat confusing (we erroneously used the word “intersected”). Please see the following transcript in page 3 lines 227-232 in the section “Mantis”:
Finally, consensus generation ensures the best combination of hits among all hits from the multiple reference data sources is selected. This combination is expanded by adding additional hits with consistent metadata (intersecting identifiers or similar functional descriptions).
To answer the reviewer questions, we aim at finding consistent information across multiple references, doing so by checking for shared identifiers or similar functional descriptions. We hypothesize that finding consistent information between independently generated sources will implicitly lead to a functional annotation that is more likely the ground-truth.
While some information is lost due to the incapacity to find consistent data across different references (either through identifiers or descriptions), our data also suggests that extensive cross-linking is already available, as many databases already provide cross-linking between multiple databases (e.g. KEGG, eggNOG, Uniprot, etc). The consensus thus leverages this feature of most databases. This cross-linking has not been leveraged only by us, but many other projects (e.g. https://doi.org/10.1093/nar/gkv1117).

R2C21:

Powered by Editorial Manager® and ProduXion Manager® from Aries Systems Corporation

Again, in "Methods - using multiple reference datasets": "For the integration of multiple reference datasets, a two-fold text mining approach was used: 1. Consensus between identifiers; and 2. Consensus between the free-text annotation description." "The consensus between identifiers is calculated by identifying intersections between the different sources. Identifiers within the free-text annotation descriptions are extracted and used here."

And also, "If no consensus between identifiers is found, then we proceed with a consensus calculation between annotation descriptions." so I guess that similarity of descriptions is only evaluated when no intersection is found among all the annotation identifiers? If this is true, then multiple identical descriptions would be kept just because a single domain is common to both reference sources? My sincere apologies, but I am missing some key point for sure here. I would ask the authors to explain in more detail how this works.
Response:
The intersection of identifiers is a fast and reliable approach, however, when no intersection is found, we use the description similarity to find consistent hits, which is slower and less reliable. We chose to use a simple intersection of IDs (and not something like the Jaccard index) because some databases provide minimal cross-linking; in addition, if two functional annotations for the same protein sequence share at least one ID, then it is likely they are functionally similar or at least related (assuming independent references naturally converge to the same function).
In terms of the metadata (identifiers and text descriptions) that is kept, if two hits share an identifier (e.g. hit 1 “glucose degradation; ID1, ID2” and hit 2 “glucose degradation; ID1, ID3”) then the resulting consensus annotation would be the combination of non-repeated metadata (e.g. “glucose degradation; ID1, ID2, ID3”). Similar functional descriptions, unless exactly the same, are kept regardless of their text similarity (e.g. “glucose degradation” and “degrades glucose”). We chose to do so for two reasons: (i) eliminate the need to assess the similarity score for all functional descriptions (higher runtime), and (ii) avoid removing functional descriptions erroneously identified as highly similar (which of course still happens since similarity analysis, as most statistical tools, is also prone to false positives).

R2C22:
Also, if I understood correctly, there can be several descriptions from a single source. For example, if the right combination of hits for a query are 2 different PFAM domains, with different descriptions. How is this handled? Are all descriptions compared in a pairwise manner intra and inter reference?
Response:
If two hits are from the same reference source, these are not compared. In addition, two hits need to have a coordinate overlap of at least 70%, meaning that if a hit from Pfam is located in 3-10 and eggNOG in 8-20 and they have the same function, since their residues are not overlapping sufficiently, they are not merged in the consensus annotation. If another eggNOG hit (with the same function) now has coordinates in 5-12, then this hit and the Pfam hit are merged (since there is sufficient residues overlap). Please see the section “Using multiple reference datasets” for an explanation on this.

R2C23:
Regarding the similarity of descriptions, the authors say that "Alongside Mantis, We developed a standalone tool [58] that allows the use of multiple reference datasets through the generation of a consensus annotation. As we have shown in the supplement "Defining a similarity threshold", this tool has high specificity, thus, in the context of Mantis, it allows for the correct identification of similar free-text annotation descriptions."

I guess this "high specificity" and how the threshold is defined should be part of the main manuscript in the Methods section? Even if this is left in the supplemental, it should be explained how this "high specificity" is achieved, and also how the definition of 0.8 as threshold is not arbitrary, and it is not as good as 0.6, 0.7 or 0.9. Also they say (in supplemental) that: "In the current scenario, sensitivity is unimportant for two reasons:
1. the reference datasets from Mantis may contain data from Swiss-Prot (TPs inflation)
2. two annotations may completely agree in identifiers but, while describing the same

function, be lexically different (FNs inflation)"
which I fail to understand. They define "False-positives (FP) = similarity score is above the threshold and identifiers disagree", which they say in the introduction that it is the goal of the consensus-description annotation. Thus, if you define these as FPs, you are contradicting the goal you define in the introduction. Also, "False-negatives (FN) = similarity score is below the threshold and identifiers agree", even when FNs are not going to be processed if those annotations with common identifiers are not processed for consensus-description, it genuinely gives an idea of the FN rate you get, and thus the sensitivity of the method, which is relevant to assess the potential impact of the consensus-description method to expand the annotations from results without common identifiers.
Response:
Thank you for this comment. We expanded on this topic, please refer to R1C6.

R2C24:
Besides the above comments, I tried to understand how these v and vi steps work by looking at some examples.
Example 1: 5 descriptions from a query:
description:Response Regulator
description:Two-component systems (sub1role)
description:Signal transduction (mainrole)
description:Transcriptional regulator
description:two-component system, sensor histidine kinase ChiS
Shouldn't at least 2 of the descriptions be merged?
Response:
We assume the reviewer means the descriptions “Two-component systems (sub1role)” and “two-component system, sensor histidine kinase ChiS”, which share the sentence “two-component systems”. Despite possibly being redundant these two descriptions are quite dissimilar, through manual curation it is easy to identify them as redundant, however doing so automatically may also eliminate important descriptions. Please also see R2C21.

R2C25:
Example 2: 7 descriptions from a query
description:DNA metabolism (mainrole)
description:helicase
description:Restriction endonuclease
description:Type ISP C-terminal specificity domain
description:Restriction/modification (sub1role)
description:type iii restriction protein res subunit
description:Type III restriction enzyme, res subunit

Example 3: full annotation of a query
query_ID NOGG_merged;Pfam-A Plug;ENOG410XQM1 2 6 |
pfam:PF07715
description:tonB-dependent Receptor Plug
description:TonB-dependent Receptor Plug Domain
Response:
We assume the reviewer means the last two descriptions of both examples, in this case, we agree the descriptions are redundant. We have now implemented a description elimination redundancy method to check for these very small discrepancies. Please see the following transcript in page 10 lines 923-925 in the subsection “Methods.Using multiple reference data sources”:
Redundant (e.g., repeated identifiers or functional descriptions) or poor quality information (e.g. "hypothetical protein") is removed from the consensus annotation.
Since it would be too many to list we do not enumerate all the possible “poor quality information” in the manuscript. Please see the following code transcript regarding the redundancy elimination:

```
def remove_redundant_descriptions(self, all_descriptions):
   res = set()
   already_added = set()
   unspecific_tokens=[‘enzyme’,’protein’,domain]
   for d in all_descriptions:
       test = d.lower()
```

Powered by Editorial Manager® and ProduXion Manager® from Aries Systems Corporation

```
        for p in set(punctuation):
            test = test.replace(p, '')
        test = test.replace('\'', '')
        test = test.replace('\"', '')
        test = test.strip()
        test = test.split(' ')
        test = [i.strip() for i in test if i not in unspecific_tokens]
        test = ' '.join(test)

        if test not in already_added:
            res.add(d)
            already_added.add(test)
    return res
```

Please see the following code transcript regarding the “poor quality information”:

```
def remove_trash_descriptions(self, all_descriptions):
    res = set()
    for d in all_descriptions:
        current_d = d.strip().lower()
        if d.strip().lower() not in [
            'enzyme',
            'domain',
            'protein',
            'unknown function',
            'domain of unknown function',
            'protein of unknown function',
            'uncharacterised protein family',
            'unknown protein family',
            'uncharacterized protein',
            'uncharacterised protein',
            'uncharacterised conserved protein',
            'uncharacterized conserved protein',
            'hypothetical protein',
        ]:
            if re.search('(protein|domain|domian|family|repeat|short repeats|region) (of|with)
(unknown|unknwon) function(\s\(?[dp]uf\d{2,}\)?)?',
                    current_d):
                pass
            else:
                res.add(d)
    return res
```

R2C26:
Example 4: comparing hits with and without tax info (-od)
without -od
NP_416050.4 kofam_merged K15268 1 4 | cog:COG0697
kegg_ko:K15268 tcdb:2.A.7.3.2 description:O\-acetylserine/cysteine efflux transporter
with -od "Escherichia coli"
NP_416050.4 kofam_merged;NOGT561_merged K15268;3XP05 2 4 |
bigg_reaction:iECIAI39_1322.ECIAI39_1\835 cog:COG0697 go:0000101
go:0003333 go:0003674 go:0005215 go:0005575 go:0005623
go\:0005886 go:0006810 go:0006811 go:0006812 go:0006820
go:0006865 go:0008150 go:0015562 \ go:0015711 go:0015804
go:0015849 go:0016020 go:0016021 go:0022857 go:0031224
go:0032973 \ go:0033228 go:0034220 go:0042883 go:0044425
go:0044464 go:0046942 go:0051179 go:005123\4 go:0055085
go:0071702 go:0071705 go:0071944 go:0072348 go:0098655
go:0098656 go:014\0115 go:1903712 go:1903825 go:1905039
kegg_brite:ko00000
kegg_brite:ko02000 kegg_ko:K15268 tcd\b:2.A.7.3.2 description:O-acetylserine/cysteine efflux transporter description:May be an export pump for cysteine and other\ metabolites of the cysteine pathway (such as N-acetyl-L-serine (S) and O- acetyl-L-serine (OAS)), and for other amino acids \and their metabolites

In this case, the annotation with -od seems richer. Besides that, there is certain

	redundancy in identifiers (K15268) and possibly in descriptions also (cysteine transporter, export for cysteine).<br>Response:<br>Thank you for this comment, however please note that the quality of the annotations (regardless of using or omitting -od) is entirely dependent on the quality of the reference data sources. Should an organism contain protein sequences that have been well characterized in the reference, then inherently, the annotation will also tend to be richer. Mantis does not create any of the reference data de novo,it merely downloads and structures publicly available reference data.<br>Please note that the K15268 is not repeated, the first K15268 is in the HMM profile names columns, whereas the second K15268 ("kegg_ko:K15268") is in the annotation data. For easier parsing, hit information comes before the "|", and annotation information after the "|".<br>Lastly, regarding the description redundancy "cysteine transporter" and "export for cysteine", this issue was discussed in R2C21. Unfortunately, without the creation of a scalable biological lexicon, this issue is unavoidable. Creating a biological lexicon is not within the current scope of Mantis.<br><br>R2C27:<br>Example 5: comparing hits with and without tax info (-od) (II)<br>## without -od<br>NP_417950.1 kofam_merged;NOGG_merged COG0306;K16322 2 3 | enzyme_ec:3.4.14.10 enzyme_ec:5.4\<br>.2.12 bigg_reaction:iECABU_c1320.ECABU_c33930<br>bigg_reaction:iECO111_1330.ECO111_4301 bigg_reaction:iNJ661.Rv0545c bi\<br>gg_reaction:iNJ661.Rv2281 bigg_reaction:iPC815.YPO3967 cog:COG0306<br>… GO terms … KEGG brite terms … kegg_ko:K01280 kegg_ko:K03306<br>kegg_ko:K03569 kegg_ko:K04043 kegg_ko:K14640 kegg_ko:K15633<br>kegg_ko:K16322 kegg_ko:K16331 … KEGG modules, pathways, ...tcdb:2.A.20.1<br>description:inorganic phosphate transmembrane transporter activity \<br>description:low-affinity inorganic phosphate transporter<br>## with -od "Escherichia coli"<br>NP_417950.1 kofam_merged;NOGT561_merged K16322;3XMW3 2 3 | bigg_reaction:iECO111_1330.ECO111_430\1 cog:COG0306 … GO terms ...<br>kegg_brite:ko00000 \ kegg_brite:ko02000 kegg_ko:K16322 tcdb:2.A.20.1<br>description:low-affinity inorganic phosphate transporter<br>description:Low-affinity inorganic phosphate transport. Can also transport arsenate<br>In this case, the without -od annotation seems richier. Again, KEGG and descriptions consensus seem to be somewhat limited.<br>Response:<br>Please see R2C26. Additionally, we also found similar results with eggNOG-mapper, where, for some organisms, annotation quality was higher without using tax_scope.<br><br>R2C28:<br>Example 6: inter-HMM integration<br>from "output_annotation.tsv"<br>YP_025301.1 Pfam-A HOK_GEF PF01848.17 5.93440414507772e-<br>22 49 8 43 2 42<br>YP_025301.1 kofam_merged K18921 - 1.3407357512953368e-23<br>49 4 46 42 89<br>YP_025301.1 NOGG_merged NOG.ENOG410ZT31.meta_raw -<br>2.1979274611398962e-27<br>49 4 46 56 102<br>Here all the 3 hits overlap.<br>From "integrated_annotation.tsv"<br>Query HMM_file HMM_hit HMM_hit_ac evalue l qs qe ss se<br>YP_025301.1 Pfam-A HOK_GEF PF01848.17 5.93440414507772e-22 49 8<br>43 2 42	pfam:PF01848 \ description:Hok/gef family<br>YP_025301.1 kofam_merged K18921 - 1.3407357512953368e-23 49 4<br>46 42 89	kegg_ko:K18921 \ description:protein HokB<br>YP_025301.1 NOGG_merged ENOG410ZT31 - 2.1979274611398962e-27<br>49 4 46 56 102	description:Hok/gef family<br>The final "consensus_annotation.tsv"<br>YP_025301.1 Pfam-A;NOGG_merged ENOG410ZT31;HOK_GEF 2 3	

Powered by Editorial Manager® and ProduXion Manager® from Aries Systems Corporation

pfam:PF01848 description:Hok/gef f\amily

Why is the Pfam included here if it does overlap with the NOGG result? Why not the kofam hit? Descriptions of PFAM and NOGG match, and the evalue is better for NOGG. I would expect either PFAM or NOGG to be discarded, and, just maybe, kofam included due to slightly different description?
Response:
The idea of the consensus generation is to attempt to find different references that point towards the same function, thus, since the hits from Pfam-A and NOGG_merged point towards the same function and overlap in residues, they are kept as the final annotation. The hit from kofam_merged does not seem consistent (at least in terms of identifiers and similarity analysis) to the other hits, in addition, this hit's coordinates also overlap with the other hits, therefore, for these two reasons, it's rejected. Residues overlap as a requirement for hit consistency was recently added, please see the following transcript in page 10, lines 911-913 in the subsection "Methods.Using multiple reference data sources":
Note that hit metadata consistency (through IDs or descriptions) requires a minimum of 70% residues overlap (default but can be changed).

R2C29:
Example 7: the same as above, but with -od "Escherichia coli"
From "integrated_annotation.tsv":
YP_025301.1 Pfam-A HOK_GEF PF01848.17 5.93440414507772e-22 49 8 43 2 42 | pfam:PF01848 d\escription:Hok/gef family
YP_025301.1 kofam_merged K18921 - 1.3407357512953368e-23 49 4 46 42 89 | kegg_ko:K18921 d\escription:protein HokB
YP_025301.1 NOGT561_merged 3XRAS - 1.4945906735751294e-15 49 6 46 7 49 | description:Hok/gef family
Final "consensus_annotation.tsv"
YP_025301.1 kofam_merged K18921 1 3 | kegg_ko:K18921 description:protein HokB

In this case only the kofam hit is kept (due to being the one with the best evalue?).
Response:
The KOfam hit does not crosslink with the other reference data sources. While Pfam and NOGG have the same description, both hits have a higher e-value and cover a smaller percentage of the protein sequence. This behaviour depends on the scoring formula, but is working as intended.

R2C30:
Example 8:
From "output_annotations.tsv"
1000565.METUNv1_03812 tigrfam_merged TIGR00092 TIGR00092 1.1e-161 363 20 \344 1 368
1000565.METUNv1_03812 kofam_merged K19788 - 2.7e-151 363 20 \344 79 446
1000565.METUNv1_03812 Pfam-A MMR_HSR1 PF01926.24 3.5e-26 363 13 \158 2 87
1000565.METUNv1_03812 Pfam-A YchF-GTPase_C PF06071.14 3.7e-42 363 284 \357 1 84
1000565.METUNv1_03812 NOGG_merged NOG.C.meta_raw - 4.7e-144 363 20 \344 273 573
From "consensus_annotation.tsv"
1000565.METUNv1_03812 kofam_merged;tigrfam_merged;NOGG_merged COG0012;TIGR00092;K19788
The PFAM domains are discarded, whereas kofam and NOGG results are included despite overlapping with with TIGRFAM, which has the best e-value.
Response:
The reason PFAM was discarded was due to the Mantis's consensus to prefer annotations where sources are consistent with each other. Unlike Pfam, all other references are interconnected:
Tigrfam metadata: GTP-binding protein YchF; GO:0005525
eggNOG metadata: K19788, GO:0005525
KOfam metadata: K19788 , COG:COG0012

Powered by Editorial Manager® and ProduXion Manager® from Aries Systems Corporation

	This example also highlights the potential of cross-linking between reference data sources, an essential feature for the generation of a consensus annotation.<br><br>R2C31:<br>Example 9:<br><br>From "output_annotation.tsv"<br><br>362663.ECP_0061 kofam_merged K02336 - 0.0 783 41 \743 96 876<br>362663.ECP_0061 Pfam-A DNA_pol_B PF00136.22 5e-47 783 385 \744 22 414<br>362663.ECP_0061 NOGG_merged NOG.COG0417.clustalo_raw - 2.4e-127 783 70 \742 872 1449<br>362663.ECP_0061 Pfam-A DNA_pol_B_exo1 PF03104.20 4.6e-10 783 197 \290 241 337<br>362663.ECP_0061 tigrfam_merged TIGR00592 TIGR00592 8.4e-49 783 217 \736 597 1141<br><br>From "consensus_annotation.tsv"<br><br>362663.ECP_0061 kofam_merged;NOGG_merged K02336;COG0417<br><br>In this case TIGRFAM and PFAM are discarded, whereas NOGG is kept despite overlapping with kofam hit and having worse e-value.<br>Response:<br>The hits K02336 (the hit with the best e-value) and COG0417 share the kegg identifier K02336, which is why they are merged.<br><br>R2C32:<br>Overall, I have problems understanding steps v and vi: inter-HMM integration and consensus generation. In my opinion, the explanations about how the algorithm works should be improved, made more accurate and exhaustive, at least in the Methods section. It could be needed to adjust some of the procedures/parameters to obtain more comprehensive and less redundant annotations, which should be one of the major advantages of Mantis according to the authors.<br>Response:<br>Thank you for the exhaustive review of the algorithms, it really highlighted the need to provide more and better examples. We have prepared new figures and examples for the manuscript. We have also explained the reasoning behind some of the given examples, we hope it clarifies steps taken and removes all doubts. Please also see R1C1.<br><br>R2C33:<br>I think that if authors want to state that Mantis is scalable to metagenomes they should provide data supporting this in the main text. According to the results in the supplements, it is indeed scalable, in my opinion, although I ran a test with a sample with 100k queries and got rather slower results, but capable of running the analysis in a decent amount of time (120946 secs → 33.60 hours) anyway.<br>Response:<br>There are several parameters to make a tool scalable, one of the most important is workflow efficiency. Mantis execution time directly scales with the number of protein sequences, which is, of course, due to the fact that with more protein sequences, more searches need to be performed against the reference data. While an iterative execution approach scales linearly, we parallized all the steps in Mantis, thus tremendously reducing execution time.<br>Since parallel work needs to be distributed across different physical processors, hardware will also play a very important role. These include number of processors, processor speed, available RAM, read/write disk speed, etc.<br>When we state that Mantis is scalable, we say so for two main reasons - we parallelized the execution and we made sure that RAM usage is kept as low as possible. In this manner, Mantis can be used in a personal computer or on an HPC platform, where execution time will depend on the amount of sequences and the available hardware (which Mantis will automatically detect).

Powered by Editorial Manager® and ProduXion Manager® from Aries Systems Corporation

As such, scaling to metagenomes does not mean much, as it is only the annotation of a larger amount of protein sequences. Regardless, we also annotated different environmental samples to prove Mantis has no issues scaling to metagenomes. This has now been moved from the supplements to the main manuscript as section “Annotating metagenomes”.

R2C34:
Methods - Establishing a test environment "True-Positives (TP) are evaluated via two main methods: i) identifiers match and ii) description match"

Each query can have multiple annotation items (identifiers and descriptions). Each item that matches with one of the reference is a new TP? Or a TP is the annotation of query as a whole? This should further explained.

The same for FP "Annotations which do not match with the reference annotation are classied as False-Positives (FP)."

Is it the whole annotation or each of the items (identifiers and descriptions) could be a FP or TP independently of the others?
Response:
All the classes in the confusion matrix correspond to an evaluation of the whole sequence annotation. Please see R1C2.

R2C35:
How are FPs, TPs ... computed when benchmarking SwissProt proteins? What are the annotation identifiers used from SwissProt? Or is it only description used?
Response:
The previous benchmark included, besides IDs, an analysis of the text-similarity between functional descriptions. We changed the benchmark to only take into account identifiers (enzyme ECs, Gene ontology (GO) IDs, eggNOG IDs, KEGG orthology (KO) IDs, Pfam IDs, and TIGRfam IDs). The confusion matrix construction is the same across all benchmarks done in the paper. We added a more thorough description of this process. Please also see R1C4.

R2C36:
"Whenever Mantis outputs a valid annotation but the reference is either of poor quality or non-existent, then this annotation is considered a potentially new annotation." (Methods - Establishing a test environment)

I think this sentence is a bit obscure. What is a "valid annotation" from Mantis, in comparison with a reference which is of "poor quality or non-existent", and what does mean for FPs and TPs the consideration of "potentially new annotation"? Is it not considered as a TP, a FP, neither? Shouldn't references of poor quality or non-existent just be removed from the benchmark?
Response:
We opted not to remove these poor-quality/non-existent reference annotations from the benchmark, but instead to mark them as PNA (if the tool annotates the respective sequence). We think this is important since it can hint at a tool’s capacity to discover novel functions. While we consider UniProt the gold-standard during the benchmark, we know this is not exactly true; while UniProt does provide very high-quality annotations, it also contains plenty of poor-quality and automatically generated annotations. Please also see R1C5.

R2C37:
"Annotation coverage is defined here as the number of Mantis' annotations divided by the total amount of protein sequences in a sample." (Methods - Establishing a test environment)

Is this the same as the % of queries which have received (consensus) annotation?
Response:
This is not only valid for Mantis, but also the other tools. In the case of Mantis, yes, it is the same as the amount of queries/sequences that received the consensus annotation, out of all the sample sequences.

Powered by Editorial Manager® and ProduXion Manager® from Aries Systems Corporation

R2C38:
"As seen in supplemental Table 1, being more permissive (by using a higher e-value threshold e.g. 1e - 3) resulted in a higher annotation coverage and a higher precision." (Analysis - Initial quality control - Function assignment e-value threshold)

It is surprising that higher e-value threshold leads to higher precision: "This is due to Mantis' internal quality control in the form of the DFS hit-processing algorithm. Being too strict with the e-value threshold constricts the available solution space, resulting in a lower precision." However, expanding the solution space would yield higher TP rate, which potentially could lead to higher precision if FP rate does not increase so much as TP rate. However, in supplements.xlsx it is shown (e-value tab) that absolute number of FPs are reduced when using a higher e-value, and this is much more surprising. Although this could be true, the authors should check this result is an artifact of the definitions and methods used to compute TP/FPs should be discarded. If the authors really did, my apologies, and also maybe they could further explain why this happens.
Response:
Thank you for pointing this out. We redid the benchmark and found similar results. As an example, please imagine two scenarios, in the first we run Mantis with an e-value threshold of 1e-3 and obtain nine hits (five from the same database and four from other different databases); in the second we run Mantis with an e-value threshold of 1e-30 and obtain five hits (all from the same database). In the first scenario, out of all these hits, during hit-processing, we find that four hits from different databases point towards the same function, however, these don’t necessarily appear to be the most significant (e.g. four with e-values above 1e-30 and another with e-value 1e-35). In the second scenario, from the previous hits, only the hit with e-value 1e-35 is present (since all others are above the e-value threshold). In the first scenario, during hit processing Mantis decides to pick these five functionally consistent hits since they come from five different databases, whereas in the second scenario another hit is chosen (e.g. a hit with e-value 1e-36).
In the first scenario the final annotation will contain the unified functional annotation of the five hits, which incidentally will contain identifiers that match with the reference annotation and this is then considered a TP. In the second scenario the hit chosen does not contain any identifiers that match with the reference annotation and so it is marked as a FP.
This is of course a limitation of the benchmark (since we are limited to evaluating IDs) but more importantly also due to the fact that a lower threshold severely limits amount of available information and thus the integration of data from different databases.
The e-value threshold will also change the combination score during hit processing.
We have added this discussion (with an example) to the supplemental PDF for readers that might question the same. While we agree that these results are counter-intuitive (and hard to explain due to the amount of different parts in the workflow), we hope we provided a simple example that explains why this may occur.

R2C39:
Initial Quality Control "When comparing the hit processing algorithms we found that the DFS algorithm consistently outperformed the other algorithms, with an average precision 0.038 and 0.013 higher than the BPO and heuristic algorithms respectively." Is this difference statistically significative?
Response:
Due to the “low” amount of samples benchmarked we did not find it appropriate to test the significance of the differences between the algorithms (due to low power). We now include a new section “Impact of hit processing algorithms” where we create synthetic samples and test the impact of the algorithms on F1 score.

R2C40:
"for homology search, Mantis uses HMMER [23] and eggNOG-mapper uses Diamond [22]." (Methods - Establishing a test environment)
This is not entirely true, since eggNOG-mapper can use both HMMER and/or Diamond, depending on the eggNOG-mapper version being used. I would rather prefer "and for eggNOG-mapper we used the Diamond-based search".
Response:
Thank you for this remark, we initially checked with one of eggNOG-mapper‘s developers (coincidently, the reviewer) regarding this, and we found, at the time, that

	the current eggNOG-mapper v2 could not use HMMER, only Diamond. Please see the issues here (https://github.com/eggnogdb/eggnog-mapper/issues/219) and here (https://github.com/eggnogdb/eggnog-mapper/issues/177).<br>And also from HMMER search options in eggNOG-mapper's GitHub page here (https://github.com/eggnogdb/eggnog-mapper/wiki/eggNOG-mapper-v2-*refactor*#HMMer_search_options):<br>"requires -i FILE. Currently, it is only available for custom databases, and it does not provide annotations. see hmm_mapper.py documentation."<br>As can be read, HMMER is only allowed for custom databases but does not provide annotations (which somewhat defeats the whole point). Since this may change in the future, we changed the sentence to what the reviewer suggested.<br><br>R2C41:<br>"Mantis ran with the following command: python mantis run_mantis -t sample.faa -od "NCBI ID" eggNOG-mapper was executed with the following command: python emapper.py -i sample.faa -o output_folder -m diamond" (Methods - Establishing a test environment)<br>According to this, the comparison is performed between Mantis using -od but eggNOG-mapper without taxonomy parameter. For results to be comparable, both Mantis and eggNOG-mapper should be run with (-od for Mantis, --tax_scope for eggNOG-mapper) and without taxonomy.<br>Response:<br>We first ran eggNOG-mapper using target_taxa (which did not work as expected, so we removed it), the tax_scope was added in version v2.0.2-rf1 (which did not exist when we first ran the benchmark). We reran eggNOG-mapper with and without tax_scope. Each sample was annotated by calculating its respective taxonomic lineage (e.g. Escherichia coli would run with "--tax_scope 561,1236,1224"), and adding it to the tax_scope during eggNOG-mapper's execution.<br><br>R2C42:<br>Also regarding comparison of tools, I don't understand why comparison with other tools have not been performed, or those already done (included in supp xlsx) are not included in the main text.<br>Response:<br>We initially chose only to includebenchmarking against eggNOG-mapper as it is the most similar tool to Mantis. As suggested by the reviewer, we have now also added Prokka.<br>However it is to note that while Prokka is customizable, natively it includes a much smaller amount of data than Mantis (reformatting all the reference data used by Mantis so that it would be compatible with Prokka would be outside of the scope of this manuscript). It also mostly attempts to annotate metabolism-related proteins (e.g., output has one column just for EC numbers). Despite this, since Prokka is widely used, we believe it is still important to include it, if only as a point of reference to future readers.<br>Initially we also benchmarked against RAST and deepEC, however we chose not to include these in the main text for different reasons:<br>•DeepEC, this tool uses a pre-trained deep learning model which aims at annotating protein sequences with EC numbers. The lack of customization and the fact that it was pre-trained does not allow for a fair comparison against Mantis, which uses an extensive amount of HMMs. Other non-reference based tools were not used for benchmarking for similar reasons.<br>•RAST uses TSHMMs (like eggNOG-mapper and Mantis), unfortunately we could not successfully use it in a local environment, therefore high-throughput testing was not feasible. RAST, like Prokka, does its own gene-calling, however, unlike Prokka, a significant amount of RAST's gene called sequences did not align to the reference sequences used during the benchmark.<br><br>Benchmarking against interproscan was not performed because this tool does not output one annotation per protein sequence. In this regard, since interproscan and Mantis can be customized to use the same data, the main difference would fall in how hits are analysed, Mantis attempts to provide the "best annotation", whereas interproscan provides all annotations. This does not allow for an objective benchmark.<br>Overall, the current functional annotation paradigm is to create high-quality reference datasets, which is then used to discover novel functions or to functionally characterize

Powered by Editorial Manager® and ProduXion Manager® from Aries Systems Corporation

	samples. Mantis does not attempt to provide novelty in this manner, instead its goal is to be adaptable towards different goals whilst making the “best” use of the available data.<br><br>R2C43:<br>Also, running Mantis through the CAFA benchmark would be highly recommended (https://genomebiology.biomedcentral.com/articles/10.1186/s13059-019-1835-8)<br>Response:<br>Thank you for the suggestion, however, we opted not to perform this benchmark for several reasons:<br>1.GO terms centric benchmark : one of the reasons Mantis uses multiple reference data sources is to improve cross-linking in posterior data analysis, benchmarking only against GO terms does not capture this feature.<br>2.GO terms output broadness: Mantis and eggNOG-mapper both use the eggNOG reference data source. This reference contains extensive cross-linking, however, for some “network-based” IDs (e.g., GO), it has been designed so that each functional annotation contains a broad list of related IDs. While this proves advantageous in annotation completeness, it also comes with the downside of not allowing for a proper benchmark of these IDs (i.e., GO terms) against a reference annotation. For example, should a functional annotation contain the ID GO:0000451 (rRNA 2’-O-methylation), it will also contain the full list of related GO terms, including very broad GO terms (e.g., GO:0009987 Cellular process). Therefore, if a comparison is done between a PFA tool annotation and a reference annotation that happens to be linked to a cellular process (which is very likely), then this would result, at least in our benchmark, in a TP, when in fact it the two annotations could be referring to a different function altogether. Unfortunately, finding the leaf/terminal node in the gene ontologies network is not trivial (since it is not a truly hierarchical network). For the reasons above, during benchmark, we limit the amount of IDs of the same type (limit of 10 - empirically determined; please see quality_benchmark.py in the supplements), which in many instances excludes GO terms, instead benchmarking with the remaining types of IDs (e.g. Pfam).<br>3.Reference based approach - from our understanding CAFA is targeted towards machine learning approaches. While it is still feasible to benchmark with CAFA, since Mantis is reference-based this dataset is not very relevant.<br>4.CAFA is a subset of Swiss-Prot - the main reason we decided not to use it was because CAFA is a selection of Swiss-Prot protein entries. Mantis was already benchmarked against a much more extensive list of Swiss-Prot entries, therefore while CAFA is a valid benchmark, we did not find it as comprehensive as our own benchmark.<br><br>R2C44:<br>Finally, in my opinion precision (TP / TP + FP) is only part of the story, and besides "Annotation coverage", other metrics should be provided, specially some considering FNs (Sensitivity), which is the other part of the story I guess.<br>Response:<br>We have now included two other metrics in the manuscript, specifically recall/sensitivity and F1 score (harmonic mean of recall and precision). Indeed the F1 score is now used as a performance metric throughout the manuscript (the previous manuscript used precision). In addition, the supplements also contain all the confusion matrix classes, alongside other confusion matrix-derived metrics.<br><br>R2C45:<br>Finally, just 2 comments about the GitHub. I would be grateful if the web will point out that NOGT and NOGG are from eggNOG, for example here: "NOGT is the collection of taxon specific HMMs, NOGG the collection of all HMMs."<br>Response:<br>This has now been rectified in the GitHub page.<br><br>R2C46:<br>Also, I noticed that when using the "eggnog.db" sqlite3 file an index is created for the "name" field. I am not sure this is necessary, since "name" it is already a primary key in the "eggnog" table. I am not an expert on sqlite3 databases though.<br>Response:<br>The reviewer is correct, we removed this line from the code.
**Additional Information:**	

Powered by Editorial Manager® and ProduXion Manager® from Aries Systems Corporation

Question	Response
Are you submitting this manuscript to a special series or article collection?	No
**Experimental design and statistics**<br><br>Full details of the experimental design and statistical methods used should be given in the Methods section, as detailed in our Minimum Standards Reporting Checklist. Information essential to interpreting the data presented should be made available in the figure legends.<br><br>Have you included all the information requested in your manuscript?	Yes
**Resources**<br><br>A description of all resources used, including antibodies, cell lines, animals and software tools, with enough information to allow them to be uniquely identified, should be included in the Methods section. Authors are strongly encouraged to cite Research Resource Identifiers (RRIDs) for antibodies, model organisms and tools, where possible.<br><br>Have you included the information requested as detailed in our Minimum Standards Reporting Checklist?	Yes
**Availability of data and materials**<br><br>All datasets and code on which the conclusions of the paper rely must be either included in your submission or deposited in publicly available repositories (where available and ethically appropriate), referencing such data using a unique identifier in the references and in the "Availability of Data and Materials" section of your manuscript.	Yes

Powered by Editorial Manager® and ProduXion Manager® from Aries Systems Corporation

Have you have met the above requirement as detailed in our Minimum Standards Reporting Checklist?	

LaTeX - Main Document (TeX file)
Click here to access/download;Manuscript;main.tex

Placeholder for OUP logo oup.pdf

Placeholder for journal logo gigascience-logo.pdf

GigaScience, 2017, 1–14

doi: xx.xxxx/xxxx
Manuscript in Preparation
Paper

PAPER

Mantis: flexible and consensus-driven genome annotation

Pedro Queirós[1,*], Francesco Delogu[1], Oskar Hickl[2], Patrick May[2,†] and Paul Wilmes[1,‡]

[1]Systems Ecology, Luxembourg Centre for Systems Biomedicine, University of Luxembourg and [2]Bioinformatics Core, Luxembourg Centre for Systems Biomedicine, University of Luxembourg

[*]pedro.queiros@uni.lu
[†]patrick.may@uni.lu
[‡]paul.wilmes@uni.lu

Abstract

Background The past decades have seen a rapid development of the (meta-)omics fields, producing an unprecedented amount of high-result ion and high-fidelity data. Through the use of these datasets we can infer the role of previously functionally unannotated proteins from single organisms and consortia. In this context, protein function annotation can be described as the identification of regions of interest (i.e., domains) in protein sequences and the assignment of biological functions. Despite the existence of numerous tools, some challenges remain, specifically in terms of speed, flexibility, and reproducibility. In the era of big data analysis, it is also increasingly important to cease limiting our findings to a single reference, coalescing knowledge from different data sources, and thus overcoming some limitations in overly relying on computationally generated data from single sources.
Results We implemented a protein annotation tool - Mantis, which uses database identifiers intersection and text mining to integrate knowledge from multiple reference data sources into a single consensus-driven output. Mantis is flexible, allowing for the customization of reference data and execution parameters, and is reproducible across different research goals and user environments. We implemented a depth-first search algorithm for domain-specific annotation, which significantly improved annotation performance compared to sequence-wide annotation. The parallelized implementation of Mantis results in short runtimes while also outputting high coverage and high-quality protein function annotations.
Conclusions Mantis is a protein function annotation tool that produces high-quality consensus-driven protein annotations. It is easy to set up, customize, and use, scaling from single genomes to large metagenomes. Mantis is available under the MIT license available at **https://github.com/PedroMTQ/mantis**.

Key words: bioinformatics; consensus; homology; HMM; protein function annotation;

Background

On a cellular scale, life is, in essence, the activity and the interaction of a plethora of different molecules, among which proteins are responsible for the vast majority of processes. A primary task in understanding how biology works is to resolve its actors properly (e.g., the proteins) and place them into context. The past decades have seen the development of the (meta-)omics fields, unlocking an unprecedented amount of data and deepening our understanding in several fields of biology [1, 2]. Alongside the evolution of the technologies and the increase in data volume, the identification of proteins transitioned from purely experimental techniques (e.g., chemical essays and spectroscopy) toward the computational-based sequence analysis thanks to the discovery of the relationship between

Compiled on: March 22, 2021.
Draft manuscript prepared by the author.

conservation of proteins' functions and sequences [3]. Therefore, the current challenges are to make use of the vast number of protein sequences and annotations available and to link new protein sequences to the previously established knowledge. High-throughput methods, such as next-generation sequencing, are able to produce a large amount of data which then needs to be analysed and interpreted. One of the ways to make sense of this data is through protein function annotation (PFA), which is, in the context of this paper, the identification of regions of interest (i.e., domains) in a sequence and assignment of biological function(s) to these regions. This strategy has proven effective in the study of single organisms as well as consortia [4, 5, 6, 7, 8, 9]. Function prediction is based on reference data, i.e., transferring the function from protein X to the unknown protein Y if they are highly similar [3]. Different approaches may be used, the most common being the comparison of an unknown protein sequence to reference data composed of well-studied and functionally annotated proteins (homology-based methods) [10, 11, 12, 13, 14, 15, 16]. Other methods may infer function through the use of machine learning [10, 17], protein networks [18, 19], protein structure [20], or genomics context-based techniques [21], but these will not be covered in this paper. For sequence alignment, BLAST [22] or Diamond [23] are commonly used, whereas, for hidden Markov models (HMM) profiles, HMMER [24] is most widely used. In PFA, these tools are often integrated into larger pipelines to provide enhanced output interpretability, workflow automation, and parallelization [14, 15, 16, 25]. Some PFA tools target specific taxa [26], others are designed with large-scale omics analysis in mind [27, 28, 29]; indeed, each PFA tool is designed to cater to its niche research topic. While experimental validation remains the gold standard, PFA, despite its many shortcomings [30], is an increasingly valuable strategy that aims to tackle the progressively more difficult task of making sense of the large quantities of data being continuously generated.

The most common method of processing candidate annotations (i.e., sequences or HMM profiles that are highly similar to the query sequence) is done by capturing only the most significant candidate ("best prediction only", hereinafter called the **BPO** algorithm). This PFA approach works well for single-domain proteins, but multi-domain proteins may have multiple putative predictions [31, 32, 33], whose location in the sequence may or may not overlap. This selection criterion may potentially lead to missing annotations and is therefore not suitable in complex PFA scenarios. To tackle this problem, domain-specific PFA is necessary. A simple approach, previously discussed in Yeats et al.[31], would be to order the predictions by their significance and iteratively add the most significant one, as long as it does not overlap with the already added predictions (henceforth referred to as the **heuristic** algorithm). Due to the biased selection of the first prediction, this algorithm does not guarantee an optimal solution (e.g., a protein sequence may have multiple similarly significant predictions). It has been previously shown that incorporating prediction significance and length may produce better results [34]. We implemented a Depth-First Search (**DFS**) algorithm that improves on the previous approaches.

The selection of reference HMMs is also critical, as PFA will ultimately be based on the available reference data. Whilst using unspecific HMMs to annotate a taxonomically classified sample may result in a fair amount of true-positives (correct annotations), depending on how strict the confidence threshold is, it may also increase the false-positives (over-annotation, due to a high confidence threshold) or false-negatives (under-annotation, due to a low confidence threshold) [35]. Using taxa-specific HMMs (TSHMM) rather than unspecific HMMs should, in principle, provide better annotations on a taxonomically classified sample, a feature that is already integrated into some PFA tools such as eggNOG-mapper [15] and RAST [16]. In essence, TSHMMs-based annotation limits the available search space, which may have positive and negative consequences. Since the search space is more specific, the annotations produced should be of higher quality; however, this higher specificity of the TSHMM could also lead to under-annotation (incomplete reference TSHMMs) or mis-annotations (low-quality reference TSHMM) [36]. This underlines the necessity to use specific (e.g., TSHMMs) and unspecific HMMs in a complementary manner. In this regard, the use of multiple sources of reference data remains a challenging aspect of PFA, and, with multiple high-quality reference data sources available, it is increasingly important to coalesce knowledge from different sources. While some PFA tools allow for the use of multiple reference data sources, either as a separate [25] or a unified [15, 37] database, it is still challenging to integrate multiple data sources dynamically.

When using reference data from multiple high-quality sources, the most common and straightforward approach is to consider the output from each reference data source independently (e.g., [25]). However, by doing so, we overlook that many sources can overlap and/or complement each other. Commonly this is compensated via manual curation, which is feasible only for a limited number of annotations. An automated approach would be to assume only the most significant annotation source for any given sequence and disregard other sources; this may result in vast losses of potentially valid and complementary information (e.g., database identifiers). As this is not desirable, the challenge is both in deciding which source(s) provide the best annotation as well as identifying complementary annotations. In the current context, complementary annotations can be defined as functional annotations that are functionally similar but originate from difference data sources; as such, while functionally similar, different data sources are likely to contain information that is absent in other data sources and vice versa. This unique functional information (i.e., database identifiers or functional descriptions) may prove essential in downstream data analysis. A straightforward approach to verify if functional annotations are functionally similar is to check whether they share a database identifier (ID), for example:

i. Function: "Responsible for glucose degradation"; IDs: K00844, EC:2.7.1.1, **PF03727**
ii. Function: "Responsible for glucose degradation"; IDs: P52789, **PF03727**, IPR022673

We can observe that the annotations (i) and (ii) share the database ID **PF03727**, thus it can be concluded that these annotations are functionally similar. If we were only to select the first annotation, we would ignore potentially useful information (IDs P52789 and IPR022673). However, it may be the case that no IDs are shared between the different annotations, for example:

i. Function: **"Responsible for glucose degradation"**; IDs: K00844, EC:2.7.1.1
ii. Function: **"Responsible for glucose degradation"**; IDs: P52789, IPR022673

We can observe that even though the annotations (i) and (ii) no longer share an ID, they still have the same function "**Responsible for glucose degradation**". Humans can quickly surmise that these annotations are the same as they share the same function description. Should the descriptions be identical or very similar, a machine could achieve the same conclusion with relative ease. However, in our experience, these free-text functional descriptions are often moderately or heavily dissim-

ilar [38, 39], with only a few keywords allowing us to ascertain they are indeed the same. This then makes it more difficult to use multiple reference data sources. For example:

i. Function: "Responsible for **glucose degradation**"; IDs: K00844, EC:2.7.1.1
ii. Function: "Protein is an enzyme and it is responsible for the **breakdown** of **glucose**"; IDs: HXK2_HUMAN

In such a scenario, someone trained in a biology-related field can quickly identify the most important words ("degradation"/"breakdown" and "glucose") in both sentences and conclude both annotations point to the same biological function. The challenge is now to enable a machine, deprived of any intellect and intuition, to eliminate confounders (ubiquitous words, e.g., "the"), identify keywords and their potential synonyms, and reach the same conclusion. A possible strategy is to use text mining, which is the process of exploring and analysing large amounts of unstructured text data aided by software, identifying potential concepts, patterns, topics, keywords, and other attributes in the data [40]. Text mining has been previously used with biological data [41, 42, 43, 44, 45], and even more specifically with regards to gene ontologies [46, 47, 48, 49, 50, 51] and PFA [43]. However, to our knowledge, there is no tool for the dynamic generation of a consensus from multiple protein annotations. This paper solves the problem of scaling the integration of different annotation sources, integrating a compact and flexible text mining strategy. We implemented a two-fold approach to build a consensus annotation, first by checking for any intersecting annotation IDs and second by evaluating how similar the free-text functional descriptions are. This approach addresses three very relevant issues with PFA [35, 36, 52, 53]: over-annotation, under-annotation, and redundancy. Under-annotation is addressed through the use of multiple reference data sources, which implicitly leads to a wider search space. Over-annotation is minimized through the generation of a consensus-driven annotation, which identifies and merges annotations that are consistent (i.e., similar function) with each other (e.g., if three out of five independent sources point towards the same function and two others point towards other, unrelated functions, then these three annotations are more likely to be valid), and eliminating the remaining inconsistent annotations. Redundancy is eliminated by removing duplicate database IDs and/or extremely similar descriptions.

Another challenge in PFA is the lack of flexibility of some tools, as these are often intrinsically connected to their in-house generated reference data, and therefore hard to customize. In contrast, we developed a tool that, while offering high-quality unspecific and specific HMMs, is independent of its reference data, thus being customizable and allowing dynamic integration of new data sources.

We hereby present Mantis, a Python-based PFA tool that overcomes the previously presented issues, producing high-quality annotations with the integration of multiple domains and multiple reference data sources. Mantis automatically downloads and compiles several high-quality reference data sources and efficiently uses the available hardware through parallelized execution. Mantis is independent of any of the default reference data, resulting in a versatile and reproducible tool that overcomes the challenge of high-throughput protein annotation coming from the many genome and metagenome sequencing projects.

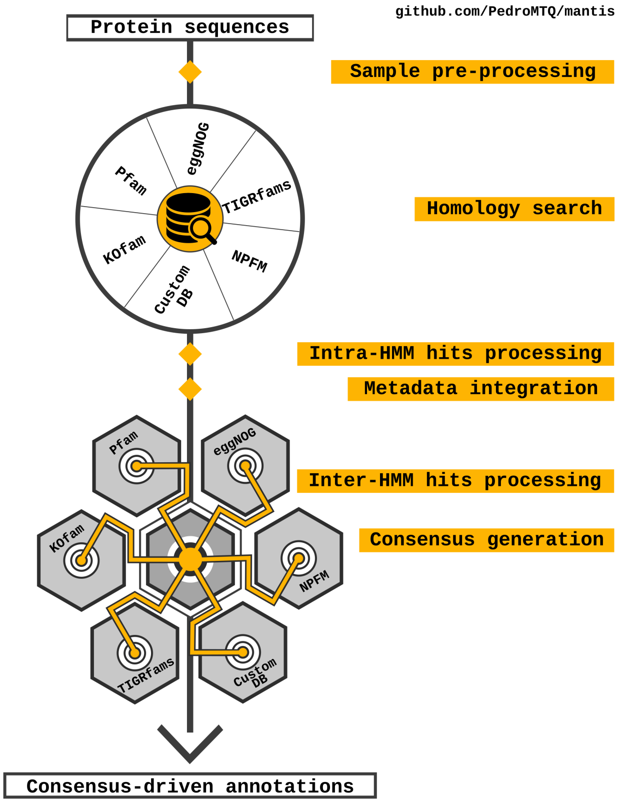

Figure 1. Overview of the Mantis workflow. KOfam [54], Pfam [55], eggNOG [56], NCBI protein family models (NPFM) [57], and TIGRfams [58] are the reference HMMs currently used in Mantis. CustomDB can be any HMM library provided by the user.

Mantis

Mantis is available at `https://github.com/PedroMTQ/mantis`, and its workflow (see **Figure 1**) consists of six main steps: (i) sample pre-processing, (ii) HMM profile-based homology search, (iii) intra-HMM hits processing, (iv) metadata integration, (v) inter-HMMs hits processing, and (vi) consensus generation. For future reference, an instance when an HMM matches with a protein sequence is referred to as a "hit". The workflow starts with sample pre-processing, in which the sample(s) is/are split into chunks. This is followed by homology search, where query sequences are searched against the available reference data using HMMER. During intra-HMM hits processing the DFS algorithm is used to generate and select the best combination of hits per HMM source; **Figure 2** shows how different algorithms may lead to a different selection of hits. Metadata integration adds the metadata (functional description and IDs) to the respective hits. During inter-HMMs hits processing, the DFS algorithm is used to generate all the combinations of hits from all HMM sources (in this step all hits are pooled together). Finally, consensus generation ensures the best combination of hits among all hits from the multiple reference data sources is selected. This combination is expanded by adding additional hits with consistent metadata (intersecting identifiers or similar functional descriptions). Please refer to the **Methods** section for a detailed description of all these steps. We provide default execution parameters, however, the user is free to fully customize Mantis, not only the parameters but also the reference databases used. Mantis requires a FASTA formatted protein sequence file as input, where the user can also provide the organism's taxon which will allow for taxa-specific annotation. Reference databases are downloaded automatically. The MANTIS.config file allows for configuration of the reference data,

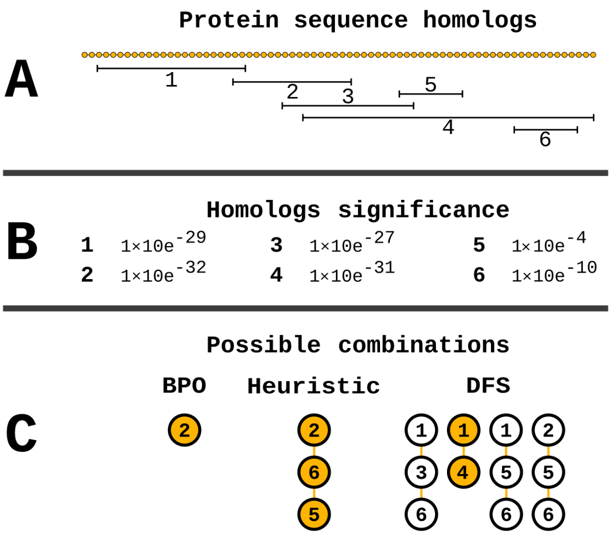

Figure 2. Homolog selection for the three hit processing algorithms in Mantis. The selection of the hit(s) depends on the underlying algorithm. In the case of the portrayed protein with six hits (A) (which are overlapping to various degree) that have varying significance values (B) the three algorithms would behave as follows: (i) BPO would select only the most significant hit (#2); (ii) the heuristic algorithm initially selects the most significant hit (#2) which then restricts (due to overlapping residues) the hits available for selection (hits #1, #3, and #4 can no longer be selected), leading to the selection of the next most significant hit (#6), and finally the selection of hit #5; (iii) the DFS algorithm generates all possible combinations of hits, which are then scored according to the e-value, hit coverage and total combination coverage (for more details, please see "**Multiple hits per protein**". According to these parameters, the most likely combinations of hits would be hits #1 and #4.

its respective weights and enables the compilation of specific eggNOG TSHMMs. For more details, see the documentation at [59]. Due to issues with Python's multiprocessing in MacOS, and the fact that HMMER is not available on Windows, Mantis is only available on Linux-based systems.

Analysis

To analyse and validate the performance of Mantis, we performed several in-silico experiments. We annotated a reference dataset containing curated protein entries from UniProt to set default parameters and evaluate the impact of different Mantis' features: (i) impact of the e-value threshold; (ii) impact of the hit processing algorithm; (iii) how each reference data source's contribution to the final output and (iv) impact of the consensus generation on annotations quality. Furthermore, we annotated several sequenced organisms, with and without TSHMMs, thus evaluating the impact of using taxa-resolved reference data. Finally, we compared Mantis against eggNOG-mapper [15] and Prokka [14]. A description of the samples used for this benchmark is available in "**Sample selection**". Prokka was only used for the annotation of prokaryotic data (i.e., all except for *Saccharomyces cerevisiae* and *Cryptococcus neoformans*). In order to compare the performance between the different tests, we calculated a confusion matrix for each test. For future reference, a **True-Positive** (TP) occurs when a functional annotation (predicted from a PFA tool) shares one or more database IDs with the respective reference annotation (e.g., Pfam ID); a **False-Positive** (FP) when no database IDs are shared; a **False-Negative** (FN) when the PFA tool does not annotate a protein sequence but a reference annotation is available; and a **True-Negative** (TN) when the PFA tool does not annotate a protein sequence and no reference annotation is available. **Precision** is defined as $\frac{TP}{TP+FP}$, **Recall** as $\frac{TP}{TP+FN}$, and **F1 score**(harmonic mean of precision and recall) as $2 \times \frac{Precision \times Recall}{Precision+Recall}$. The F1 score is used as a performance metric. Further details on the benchmark are available in "**Establishing a test environment**".

Initial quality control

Function assignment e-value threshold

It is known that the e-value threshold directly affects annotation quality, however, no gold-standard threshold exists [34]. Depending on the reference data source's size, quality, and specificity, we may use more or less stringent thresholds. It is therefore essential to test annotation quality with different thresholds. As such, we tested different static e-value thresholds and a dynamic threshold, which has been described in "**Testing different e-value thresholds**". As can be seen in the supplemental **Table 1**, precision was similar across the range of e-value thresholds tested, with recall/sensitivity decreasing with lower e-value thresholds. Unexpectedly, unlike recall, precision was not directly correlated with the e-value threshold; indeed a maximum precision of 0.747 was obtained for the e-value threshold $1e^{-6}$, with precision slightly decreasing with more stringent e-value thresholds. A maximum F1 score of 0.827 was observed for the e-value threshold $1e^{-3}$, as such, we chose this value as the default e-value threshold for Mantis.

Impact of hit processing algorithms

To understand whether the different hit processing algorithms resulted in statistically significant differences in F1 scores, we created synthetic samples and performed pairwise comparisons between the DFS and the other algorithms: (i) DFS and heuristic, and (ii) DFS and BPO. We rejected the H_0: "no differences in F1 score between the tested algorithms" in both comparisons since p-value < 0.01. The DFS algorithm resulted in a greater F1 score (mean = 0.827) than the heuristic (mean = 0.826) and BPO (mean = 0.816) algorithms. Further details on results can be found in the supplemental **Table 2**, and further details on the testing method can be found in "**Testing hit processing algorithms**".

Impact of sample selection

Testing exclusively against well-annotated organisms is a recurring issue with protein annotation benchmarking, resulting in the re-annotation of sequences already present in the reference data used, leading to a biased annotation quality evaluation. To avoid this bias, we downloaded all the curated UniProt (i.e., Swiss-Prot) protein entries (as of 2020/04/14) and selected entries by their creation date such that we have four samples that contain protein entries created in different date ranges (2010-2020, 2015-2020, 2018-2020, and 2020). Samples with more recent protein entries are increasingly more likely to lack any proteins used to generate Mantis' reference data, which increases the likelihood that potential annotations are due to true sequence homology (and not to circular re-annotations). We annotated these samples using three different hit processing algorithms (DFS, heuristic, and BPO), determining the impact of each on the F1 score.

As seen in **Figure 3**, the F1 score decreased as the sample was restricted to more recent data. As seen in the supplemental **Table 3**, when comparing the hit processing algorithms, we found that the DFS algorithm consistently outperformed the other algorithms, with an average F1 score 0.021 and 0.003 higher than the BPO and heuristic algorithms, respectively. In addition, the F1 score difference between the multiple hits algorithms (DFS and heuristic) and the single hit algorithm (BPO) increased as the entries in a sample were restricted to more recent years.

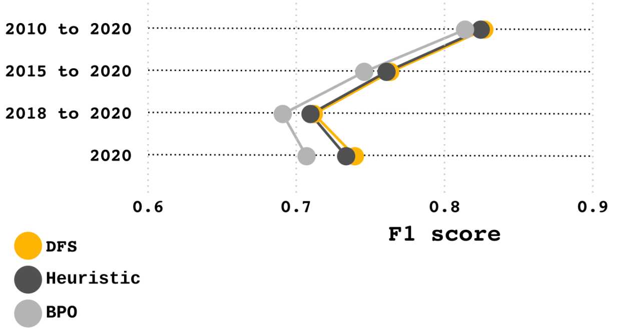

Figure 3. Annotation F1 score per hit processing algorithm and sample. The DFS algorithm outperforms the other algorithms.

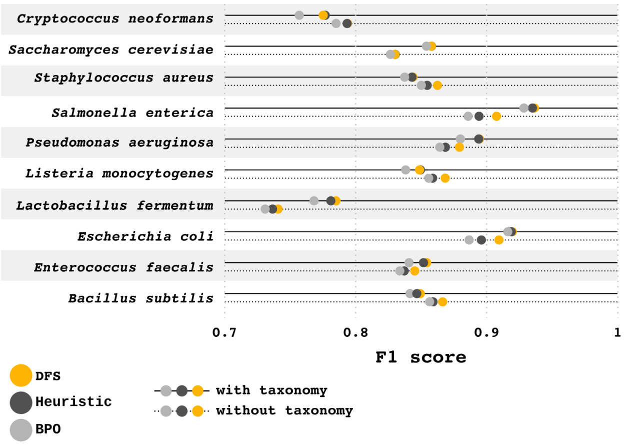

Figure 4. F1 score per hit processing algorithm and organism, with and without using taxonomy information. F1 score was higher for well-studied organisms, TSHMMs also tend to perform better with these organisms.

Contribution of the different reference data sources

We analysed each reference data source's contribution to the output annotation for the UniProt 2010-2020 sample. By checking the column "*HMM_files*" in the *consensus_annotation.tsv* file, we found that Pfam was present in 24.4% of the sequence annotations, KOfam in 62.37%, eggNOG in 76.52%, NPFM in 13.91%, and TIGRfam in 12.96%. Note that, since multiple reference data sources may be present in one sequence (due to the consensus generation and hit processing algorithms), the sum of the previous values is above 100%.

Impact of consensus generation

During consensus generation, two methods are used for checking the consistency of the hits metadata: IDs intersection and text mining. We analysed the contribution of both methods for the annotation of the UniProt 2010-2020 sample, and found that roughly 35.1% of the consistency checks were due to the text mining approach, and the remaining were due to IDs intersection.

We also tested the impact of text mining on annotation performance: to do so, we annotated the Uniprot 2010-2020 sample but restricted the consensus generation in different manners and with different algorithms. Six different test conditions were created: (i) DFS with default consensus generation, (ii) DFS with consensus generation restricted to IDs (i.e., IDs intersection but no text mining), (iii) DFS without consensus generation (i.e., neither IDs intersection nor text mining), (iv) BPO with default consensus generation, (v) BPO with consensus generation restricted to IDs, and (vi) BPO without consensus generation. We also annotated the same sample using eggNOG-mapper - condition (vii). Prokka was not used here since the current sample contains non-prokaryotic data. The F1 scores were as follows: (i) 0.827, (ii) 0.790, (iii) 0.774, (iv) 0.814, (v) 0.779, and (vi) 0.763, and (vii) 0.703. Further details can be found in supplemental **Table 4**.

Hit processing approximation

During hit processing, two algorithms may be used, the DFS, and, as a backup (if the DFS algorithm's runtime exceeds 60 seconds), the heuristic. We calculated how many times the heuristic algorithm was used as a backup during the hit processing of the 2010-2020 UniProt sample. We found that for the intra-HMM hit processing, the heuristic algorithm was used in roughly 7.2% of the sequences, and for the inter-HMMs hit processing in 0.5% of the sequences.

Quality control with sequenced organisms

As a secondary quality control, to assess the impact on F1 score when using taxa-resolved reference data, we annotated several sequenced organisms (for more details, see the supplemental **Table 5**) with and without TSHMMs. We also evaluated the impact of the different hit processing algorithms on these samples. As seen in **Figure 4**, well-studied organisms (e.g., *Saccharomyces cerevisiae*) had better annotations, especially when applying TSHMMs, unlike poorly described organisms. The average F1 score gain with TSHMMs was 0.006. With TSHMMs, the DFS algorithm had, on average, 0.001 and 0.010 higher F1 scores than the heuristic and BPO algorithms, respectively. Without TSHMMs, the DFS algorithm had, on average, 0.008 and 0.013 higher F1 scores than the heuristic and BPO algorithms, respectively. Further details can be found in the supplemental **Table 6**.

Comparison between Mantis and other PFA tools

The sequenced organisms enumerated in the supplemental **Table 5** were annotated with Mantis, eggNOG-mapper, and Prokka (for the latter non-prokaryote organisms were excluded). To evaluate the added value of using the very comprehensive eggNOG reference data source, we also assessed Mantis' F1 score using different reference data. In total, six different tests were performed for each organism: (i) Mantis with default data sources and with taxonomy information; (ii) Mantis with default data sources except for eggNOG's data and with taxonomy information; (iii) Mantis with default data sources but without taxonomy information; (iv) eggNOG-mapper without tax scope option; (v) eggNOG-mapper with tax scope option; (vi) Prokka with default data sources and default execution.
On average, (i) had a F1 score and annotation coverage of 0.857 and 96.56%, respectively, (ii) 0.832 and 89.82%, (iii) 0.850 and 96.14%, (iv) 0.734 and 88.45%, (v) 0.725 and 88.02%, and (vi) 0.507 and 62.38%. As seen in **Figure 5**, Mantis outperformed the other PFA tools in all tests (with one exception in the organism *Saccharomyces cerevisiae*, where eggNOG-mapper without taxonomy had an F1 score of 0.841 and Mantis without taxonomy had an F1 score of 0.830). The average Mantis F1 score with default execution and TSHMMs was 0.131 higher than eggNOG-mapper (with tax scope) and 0.360 higher than Prokka. Mantis' setting without the eggNOG reference data had an average F1 score 0.107 higher than eggNOG-mapper

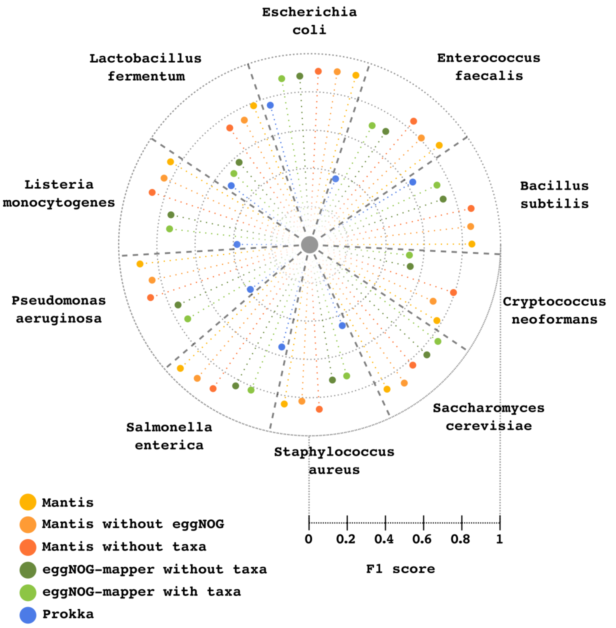

Figure 5. Annotation F1 score of Mantis, eggNOG-mapper, and Prokka using different reference data. Each slice represents an organism and contains the F1 score obtained between the different conditions.

(both tools with taxonomy information) and an average F1 score 0.025 lower than Mantis' with the eggNOG reference data. Further details are available in the supplemental **Table 7**.

Annotating metagenomes

To our knowledge, there are no manually curated metagenome annotations, therefore annotation validation was not performed, instead we only calculated the annotation coverage. We selected four samples from different environments and predicted the protein coding genes with Prodigal v2.6.3 [60]. The annotated samples were:

- Biogas highly efficient cellulose-degrading consortium (SEM1b) [61, 62] with 39411 sequences;
- Glacier-fed stream sediment (GFS) [63] with 270341 sequences (phenol-chloroform extraction batch number 37);
- Marine [64] with 605043 sequences (ERR1726751);
- Human gut microbiome (MuSt [7]) with 692061 sequences (M05-01-V1).

The performance of Mantis varied per metagenome sample; it annotated 213539, 162133, 33016, and 559792 sequences in the samples GFS, marine, SEM1b, and MuSt, respectively. The respective annotation coverage was as follows: 78.99%, 26.80%, 83.77%, and 80.89%. We repeated the same test for eggNOG-mapper and Prokka (in the case of Prokka by annotating the original nucleotide sequences), the coverage for the samples GFS, marine, SEM1b, and MuSt, was, respectively, 77.52% and 10.87%, 16.21% and 1.01%, 81.95% and 32.32%, and 78.72% and 20.37%.

Computational efficiency

We ran Mantis against samples with a different number of pseudo-random sequences and a different number of available CPUs. To generate pseudo-random sequences, we randomly selected sequences from the UniProt 2010-2020 sample, each sequence was then modified to include a random number of single nucleotide polymorphisms (deletion or insertion). We performed this test for the DFS and heuristic algorithm only. As expected, we found that the heuristic algorithm was faster than the DFS algorithm. The heuristic algorithm was, on average, 1.42 times faster than the DFS algorithm. As expected, runtimes were inversely correlated to the number of CPUs and sequences. Further details can be found in the supplemental **Table 8**.

We also aimed at allowing Mantis to be run on personal computers, which requires removing the eggNOG dataset. However, as we have previously shown in **Comparison between Mantis and other PFA tools**, this does not cause a high impact on F1 score. We annotated the previously enumerated sequenced organisms (supplemental **Table 5**) on a Dell XPS 13-9370 with Ubuntu 20.04.1 LTS 64 bit, 16GB RAM, 512 GB SSD, and an 8 core Intel Core it-8550U CPU. The average runtime for prokaryotes and eukaryotes was 28 and 93 minutes, respectively. Further details are available in the supplemental **Table 9**.

Discussion

We herein presented Mantis, an open-access PFA tool that produces high-quality annotations and is easily installed and integrated into other bioinformatic workflows. Mantis addresses some major challenges in PFA, such as flexibility, speed, the integration of multiple reference data sources, and use of domain-specific annotations. Mantis uses a well-established homology-based method and produces high-quality consensus-driven annotations by relying on the synergy between multiple reference data sources and improved hit processing algorithms.

We have shown that a stricter/lower e-value threshold did not necessarily lead to a higher F1 score. As expected, a lower threshold restricted the amount of hits, lowering the recall. However, we also found that more stringent e-value thresholds may result in a lower precision; this behaviour is connected to Mantis's consensus generation and hit combinations scoring. A thorough explanation is available in the supplemental PDF.

Well-curated and commonly used resources were chosen as the default reference data sources for Mantis, containing both unspecific and specific reference data (e.g., taxa-specific). As we have shown, no single reference data source accounted for most annotations, each offering both unique and overlapping insight into protein function, thus confirming their synergy and partial redundancy. These are integrated through a consensus-driven approach, which Mantis uses as an additional quality control step, and a means to automatically incorporate a broader variety of IDs. The intersection of IDs was, as expected, the main contributor towards this integration (since most databases provide cross-linking), however, we found that the text mining approach still contributed considerably (35.12% for the UniProt 2010-2020 sample), which clearly highlights the need to use such a method.

We additionally evaluated the impact of not using text mining during consensus generation and removing the consensus generation altogether on the DFS and BPO algorithms. The benchmark using the BPO algorithm without consensus generation represented the baseline approach towards the integration of multiple reference data sources (merely selecting the most significant hit during inter and intra-HMMs hit processing). In contrast, the benchmark using the DFS algorithm with the consensus generation depicted the accumulation of all the features introduced by Mantis. Overall, we found a difference of 0.064 in F1 scores, which suggests the additive effect of Mantis's various data integration methods. Mantis, in respect to this specific benchmark, also obtained a F1 score higher than eggNOG-mapper in all conditions, which suggests the impor-

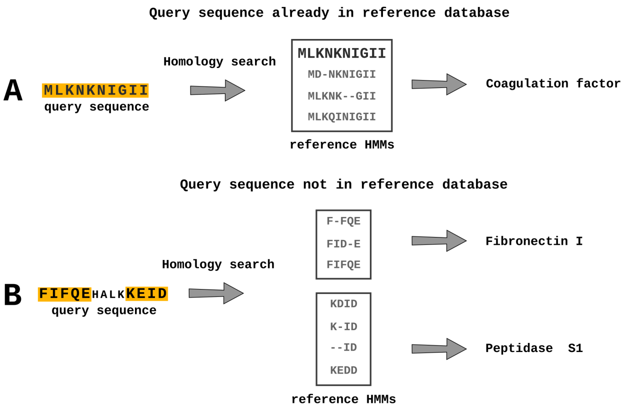

Figure 6. The impact of the reference data completeness on protein function annotation. A. the functional prediction is facilitated by the query sequence being previously identified and included in the reference HMMs. **B.** if the query sequence has not been previously annotated, multiple regions in the protein may match with different reference HMMs.

tance of using multiple reference data sources.

We have implemented two algorithms for domain-specific homologs search (DFS and heuristic as backup), and have not only shown that these algorithms perform better when annotating previously described protein sequences, but that their impact on the F1 score increased when annotating previously uncharacterized protein sequences (e.g., average F1 score gain with DFS and BPO algorithms in the UniProt 2010-2020 and 2020 samples was 0.013 and 0.033, respectively). We hypothesize that for the latter, a homology search is not capable of finding whole-sequence homologs, finding, however, multiple domains that partially constitute the protein sequence. As such, we argue that by increasing the resolution (sequence homology to domain homology) of homology-based reference data, domain-specific algorithms may become increasingly valuable. We think this would be especially important when annotating protein sequences without well-described homologs but that contain previously characterized conserved protein domains. In **Figure 6.A**, we can observe that the current query sequence is already used to generate the HMM profiles in the reference data, matching with the HMM profile containing it. Such a scenario is common when annotating well-described organisms (e.g., *Escherichia coli*). However, as is often the case when annotating non-model organisms and metagenomes, the query sequence is absent from the reference data (**Figure 6.B**), thus partially matching with several HMMs (which may correspond to multiple domains, depending on the resolution of the reference data). Unlike the BPO algorithm, the heuristic and DFS algorithms are able to incorporate multiple homologs. While these may not be enough to determine a protein's biological function, they still provide a better biological context than a single functional annotation.

Further improvements in annotation quality may also require the use of motif-based and/or genomic context-based (e.g., operon context information, co-expression, and subsystems) methods such as those described by Sigrist et al.[65], Mooney et al. [66], Mavromatis et al.[67], Overbeek et al.[21], and Hannigan et al.[68]. Nevertheless, the significantly higher F1 score seen when comparing the DFS and BPO algorithms highlights the need to adopt better hit processing methods, especially for non-model organisms. With samples ranging from thousands to millions of protein sequences, sub-optimal hit processing algorithms may cascade into unnoticeable pitfalls in downstream data analysis (e.g., accumulation of incomplete or low-quality genome annotation, which may lead to false biological interpretations). While we have shown that the DFS algorithm outperforms the heuristic algorithm, both achieve a very similar F1 score when applied to non-synthetic samples; since the heuristic algorithm is much more time efficient (as seen in supplemental **Table 8**), a user may confidently set it as primary algorithm.

The use of TSHMMs resulted in a 0.006 higher F1 score, however, this improvement (as seen in **Figure 4**) was not consistent across all the annotated organisms (as expected, a similar trend was also seen with eggNOG-mapper). We believe this is due to a poorer quality of the TSHMMs for some organisms, which is a consequence of the issues with the current taxonomy classification system [69, 70] and lack of knowledge regarding highly resolved taxa [71]. Model organisms such as *Escherichia coli* and *Saccharomyces cerevisiae* clearly benefited from TSHMMs, both since the reference data already contains data specific to these organisms and that functions of proteins within model organisms are better experimentally described. Conversely, non-model organisms are often only computationally annotated by association, contributing to a weaker reference annotation (which can be observed by the higher rate of potentially new annotations in these organisms, as seen in the supplemental **Table 6**). Nonetheless, while experimental evidence remains the gold standard, it is unfeasible to ignore the need for computational methods to infer function. While steps in this direction have been taken [56, 16], taxa-resolved PFA remains a challenge.

We benchmarked Mantis against two other PFA tools - eggNOG-mapper and Prokka, and have shown that Mantis achieves a higher F1 score (0.131 higher than eggNOG-mapper and 0.350 higher than Prokka). Although Mantis' default execution heavily relies on the eggNOG reference data, we have also shown that even without it, it is possible to achieve an almost similar F1 score. This attests to the quality of the various reference data used, showcasing as well the possibility of running Mantis on a personal computer (something that would be impossible with eggNOG's prohibitive size).

We also evaluated the annotation coverage of Mantis and the other PFA tools when annotating metagenomes. Mantis had the highest annotation coverage among the tested PFA tools, but eggNOG-mapper was close behind. All PFA tools had a low annotation coverage for the marine sample. We believe this may be due to a lack of reference HMMs for this specific environment. This metagenomic sample has data from varying ocean depths, with many novel sequences from viruses, prokaryotes, and picoeukaryotes [72].

Finally, as shown in **Accessibility and Scaling**, a conda environment and automated reference data download are provided. In addition, Mantis accepts several formats as input (i.e., protein FASTA file, TSV file with paths, directories, or compressed archives), outputting easy to parse TSV files. We believe these features address some of the reproducibility challenges the bioinformatics community still faces [73].

As discussed, there is still room for improvement in the hit processing algorithm DFS (since it does not provide large F1 score gains over the heuristic algorithm). In the future, Mantis could also include genomic context-based annotation methods. Despite the previously discussed challenges, we have clearly shown that Mantis is a flexible tool while also producing annotations with high precision and recall.

Conclusion

By making use of the synergistic nature of differently sourced high-quality reference data, Mantis produces reliable homology-based annotations. By allowing for total customization of these reference data, Mantis is also flexible, easily integrated and adapted towards various research goals. In con-

clusion, we have shown that Mantis addresses a number of the current PFA challenges, resulting in a highly competitive PFA tool.

Methods

Accessibility and Scaling

Mantis automatically sets up its reference data by downloading HMMs from different sources, and, when necessary, reformatting the data to a standardized format and downloading any relevant metadata. Reference data can be customized via a config file [74]. It also dynamically configures its execution depending on the resources available. A conda environment and extensive documentation [59] are available.

Mantis splits most of the workflow into sub-tasks and subsequently parallelizes them by continuously releasing tasks to workers from a global queue (via Python's multiprocessing module). During each main task of the annotation workflow, workers are recruited (the number of workers depends on the available hardware and work required), these will then execute all the queue tasks. When a worker has finished its job, it will execute another task from the queue, until there are no more tasks to execute. If the queue is well balanced, minimal idle time (time spent waiting for workers to get a new task) can be achieved. Load balancing is achieved by splitting the sample and reference data into chunks. During setup, large reference data sources (more than 5000 HMM profiles) are split into smaller chunks, this enables parallelization and ensures each annotation sub-task takes approximately the same time. Samples are equally split into chunks (sample chunk size is dynamically calculated). If the sample has 200,000 or fewer sequences, sequences are distributed by their length among the different chunks, so that each chunk has approximately the same number of residues. If the sample has more than 200,000 sequences, then sequences are distributed to each chunk independently of their length (this alternative method is an efficiency safeguard). This two-fold splitting achieves quasi-optimal load balancing. With the sample and reference data in chunks, posterior workflow steps can be parallelized wherever applicable.

Input and output

MANTIS accepts protein sequence FASTA files as input. If the sample has been previously taxonomically classified, the user can add this information when running Mantis. For example, if annotating an *Escherichia coli* sample, the user could add *-od* followed by the NCBI ID or the organism name:

```
$ python mantis run_mantis -t sample.faa -od 562
```

Mantis outputs, for each sample, three tab-separated files [75], each corresponding to a different step in Mantis' workflow: (i) a raw output *output_annotation.tsv* (generated during **Figure 1.Intra-HMM hits processing**), with all the hits, their e-value, and coordinates; (ii) *integrated_annotation.tsv* (generated during **Figure 1.Metadata integration**), with the same information as *output_annotation.tsv*, but also with hits metadata (e.g., KEGG orthology IDs (KO), enzyme commission (EC) numbers, free-text functional description, etc); and (iii) the main output file *consensus_annotation.tsv* (generated during **Figure 1.Consenus generation**), with each query protein ID and their respective consensus annotation from the different reference data sources (e.g., Pfam). These files provide contextualized output in a format that is both human and machine-readable. A *Mantis.out* file is also provided per sample, serving as a log file for each execution step.

Reference data and customization

Mantis, by default, uses multiple high-quality reference HMM sources – Pfam [55], eggNOG [56], NPFM [57], KOfam [54], and TIGRfam [58] (these default HMMs can be partially or entirely removed). To find more meaningful homologs through taxa-specific annotation, Mantis uses TSHMMs, originally compiled by eggNOG and NPFM. eggNOG TSHMMs were compiled by downloading all the TSHMMs at `http://eggnog5.embl.de/download/latest/per_tax_level/`, their respective metadata originates from the metadata available in the previous link as well as the metadata within the eggNOG-mapper SQL database. NPFM TSHMMs were compiled by downloading all the NPFM HMMs at `https://ftp.ncbi.nlm.nih.gov/hmm/current/` and assigning each HMM into their respective TSHMM. A general NPFM HMM was created by pooling all non-assigned HMM profiles and the TSHMMS from the following NCBI IDs: 2157 (*Archaea*), 2 (*Bacteria*), 2759 (*Eukaryota*), 10239 (*Viruses*), 28384 (Others), and 12908 (Unclassified). These IDs correspond to NCBI's top level taxonomy rank IDs. A general eggNOG HMM was created by pooling together the TSHMMs from the same aforementioned NCBI taxon IDs. The user can customize which eggNOG TSHMMs are downloaded by Mantis by adding the line *nog_tax = NCBI_ID*1, *NCBI_ID*2 to the config file. Custom HMM sources can also be added by the user, metadata integration of these is also possible (an example is available in Mantis' repository [76]). Since some sources are more specific than others, the user may also customize the weight given to each source during consensus generation [77]. HMM profiles often only possess an ID respective to the database they were downloaded from, which may not directly provide any discernible information. Mantis, when necessary, ensures that the hits from these HMMs are linked to their respective metadata. For future reference, while an HMM is an individual profile, Mantis compiles all related HMM profiles into a single file making it indexable by HMMER. Thus when a certain HMM source is mentioned, it refers to the collection of related HMM profiles.

Taxa-specific annotation

Taxa-specific annotation (TSA) uses the TSHMMs and unspecific HMM made available by eggNOG and NPFM. TSA, however, works differently from the annotation method of the other reference data. When given taxonomy information (either a taxa name or NCBI ID) the organism's taxonomic lineage is computed (e.g., for *Escherichia coli* the lineage would be `2 - 1224 - 1236 - 91347 - 543 - 561 - 562`). TSA starts by searching for homologs in the most resolved TSHMM (in this case for taxa 562, if it exists). All valid homologs (respecting the e-value threshold) are extracted for each query sequence, and unannotated sequences are compiled into an intermediate FASTA file. A new homology search round starts with the sequences in the current intermediate FASTA, but now in the TSHMM one level above (in this case the TSHMM 561). This cycle repeats until all query sequences have valid homologs or until there are no more TSHMMs to search for. If there are still sequences to annotate, then these homologs are searched for in the general eggNOG and NPFM HMMs. If no taxonomy information is given, the homology search starts with the general NPFM and eggNOG HMMs. Non-taxa specific HMMs (i.e., Pfam, KOfam, and TIGRfams) are always used, regardless of the sample's taxonomy.

Multiple hits per protein

HMMER outputs a *domtblout* file [24], where each line corresponds to a hit/match between the reference data and the query

protein sequence. The e-value threshold within the HMMER command limits the amount of hits to be analyzed in the posterior processing steps. Each hit, among other information, contains the coordinates where the query sequences matched with the reference HMM profiles and the respective confidence score (e-value) (**Figure 2.A and .B**). Mantis uses HMMER's independent e-value when using the DFS and heuristic algorithms, whereas it uses the full sequence e-value when using the BPO algorithm (since only the best hit is extracted per protein sequence). For simplicity purposes, both are simply referred to as e-value throughout this paper. The annotation of a protein sequence with multiple hits is a nontrivial problem, thus requiring the implementation of a method for the processing of hits. We designed a method that generates and evaluates all possible combinations of hits by applying the DFS algorithm [78]. This algorithm allows the traversal of a tree-structured search space (i.e., each node is a hit), whilst pruning solutions that do not respect predefined constraints (i.e., overlapping hit residues coordinates), backtracking from leaf to root until the possible solution space is exhausted. Our method generates all the possible combination hits with the following method: (i) Get one hit from the collection of hits and define it as the combination root hit; (ii) Check which other hits overlap up to 10% (default value) [31] with previous hits and select one to add to our current combination of hits; (iii) Repeat step (ii) until no more hits can be added; (iv) Repeat steps (i-iii) so that we loop over all the other hits and all possible combinations are generated. We used Cython [79] to speed up the DFS implementation. Cython is an optimising static compiler for the Python programming language, allowing the compiler to generate C code from Cython code, in this case, functioning as a wrapper for the DFS algorithm. The total number of possible combinations is $2^N - X - 1$, where N is the number of hits the protein sequence has, X the number of impossible combinations (combinations with overlapping hits), and *1* the empty combination. Due to exponential scaling, this method is not always computationally feasible (e.g., the query sequence is very large and has many small-sized hits). In such a scenario, the DFS algorithm may exceed the system's recursion limit or be unable to find a solution in optimal time (60 seconds by default, but customizable). Should this happen, Mantis employs the previously described heuristic algorithm, which scales linearly (a warning is written in the *Mantis.out* log).

After generating all the possible combinations, each combination is evaluated according to several parameters:

- $query_{length}$ - number of residues in the query sequence.
- hit_{length} - number of residues in the hit.
- $combo_{length}$ - number of hits in the respective combination.
- *Total coverage* (**TC**) – number of non-redundant residues in all the combination's hits divided by $query_{length}$. A high TC implies the combination covers a large percentage of the protein sequence.
- *Average hit coverage* (**HC**) – sum of the coverage of each hit ($\frac{hit_{length}}{query_{length}}$). This sum is then averaged by dividing by $combo_{length}$. A high HC implies the hits in the combination are large, thus benefiting combinations with a low amount of large hits rather than combinations with a high amount of small hits.
- *Combination e-value* (**CE**) – the e-value of each hit is scaled twice, once to reduce the range between different e-values (log10) and the second to understand how each hit e-value compares to the best/lowest hit e-value found for a particular sequence (minmax scaling). The scaled e-values are then summed and divided by $combo_{length}$.

The ***combination score*** is defined by the following equation:

$$TC \times HC \times CE \tag{1}$$

The combination with the highest ***combination score*** is then selected, where the available choices will ultimately depend on the algorithm used (**Figure 2.C**). Our intra-HMMs hit processing implementation thus applies a two-fold quality control, initially by limiting the amount of hits in HMMER's *domtblout* (i.e., e-value threshold) and secondly by hierarchically ordering and selecting the most significant combination of hits.

Using multiple reference data sources

An unannotated protein sequence may match with zero, one, or multiple reference HMM profiles, from one or more data sources. When a protein sequence has multiple hits from different data sources, it is important to identify functionally similar annotations so that no information is lost (i.e., functional descriptions or IDs that may be in one reference data source but not in another). By linking the metadata respective to the HMM profiles to the now annotated protein sequence, we can identify functionally similar annotations and integrate multiple reference data sources into one final consensus annotation. In this manner, functionally similar annotations are merged, and any complementary information they provide can then be used in downstream analysis (e.g., annotation 1 has a Pfam and KO ID, annotation 2 has an EC number and the same KO ID, merging these will result in a final annotation with more information).

For the integration of functional annotations from multiple data sources, a two-fold approach was used: (i) *Consensus between IDs*; and (ii) *Consensus between the free-text functional description*. The latter is used as a backup, since IDs cross-linking is not universally available. Each reference data source includes metadata relevant to the HMM profiles herein; this metadata may include multiple intra and/or inter database IDs as well as free-text functional descriptions. IDs are extracted either through source-specific metadata parsing and regular expressions. Free-text functional descriptions are extracted by source-specific metadata parsing. With this information it is then possible to identify annotations that are functionally similar/consistent, and may thus be complementary to each other. The *consensus between IDs* is calculated by identifying intersections between the functional annotations of different reference data sources (e.g., both annotations have the same Pfam ID). IDs within the free-text functional descriptions are extracted (with regular expressions) and also used here. If no consensus between IDs is found, then we proceed with a consensus calculation between functional descriptions (further described in the supplemental PDF).

Inter-HMMs hit processing starts by pooling together all hits from the different reference data sources and generating all possible combinations of hits (**Figure 7.A**). The same method used in intra-HMM hit processing is applied, where the DFS algorithm is used by default (again using the heuristic algorithm as a backup), but the BPO and heuristic algorithms can also be used. We then check the metadata consistency (either through IDs or free-text functional descriptions) of each hit against the current sequence's other hits. With this information, a metadata consistency graph is generated (**Figure 7.B**). With the metadata consistency graph and all possible combinations of hits, we can then calculate the ***consensus combination score*** using equation 2. This requires calculating of the ***combination score***, using equation 1. This score is then multiplied by an additional score, comprised of the following parameters:

- *Average hit consistency* (**HCN**) – number of hits (among all hits) with metadata directly consistent (i.e., nodes directly connected in the metadata consistency graph) to the hits in the current combination. Consistency checks are restricted to other reference data sources besides the hit own's reference source (e.g., if a hit is from Pfam, we would only check hits that are not from Pfam). This number, plus the number of hits in the combination, is divided by the total number of hits for the respective query sequence (e.g., if a combination has two hits, with these having metadata consistent with three other hits, and if there are ten hits in total, **HCN** would equal to $\frac{2+3}{10} = 0.5$). This is an important parameter since it entails independent sources are describing the same function.
- *Reference HMM weight* (**HMMW**) – average weight of all the reference data sources within the combination. This is calculated by adding all hits' HMM weights and dividing this sum by the number of hits in the combination (e.g., if a hit comes from Pfam, that has a weight of 1, and another from eggNOG, that has a weight of 0.8, **HMMW** would equal to $\frac{0.9+0.8}{2} = 0.85$). The default weight for each default reference data source has been set according to the authors' perception of the reference quality – creation method, curation level, and annotation completeness (eggNOG – 0.8, Pfam – 0.9, NPFM and KOfam – 0.7, and TIGRfam – 0.5). This weight is customizable, the default weight for custom reference data is 0.7 (which can also be customized).
- *Metadata quality* (**MQ**) – average metadata quality of each hit in the combination. If a hit has no annotation data (IDs or description) it is given a score of 0.25, 0.5 if only the description, 0.75 if only the IDs, 1 if IDs and description. All hit's metadata quality score is summed and divided by the number of hits in the combination.

Note that hit metadata consistency (through IDs or descriptions) requires a minimum of 70% residues overlap (default but can be changed). Using the previously calculated ***combination score***, we then calculate the ***consensus combination score*** using the following equation:

$$Combination_{score} \times \frac{HCN + HMMW + MQ}{3} \quad (2)$$

The combination with the highest ***consensus combination score*** is selected and expanded by concatenating additional metadata from other consistent hits (**Figure 7.C**). In this step, consistent hits can be either directly or indirectly connected in the metadata consistency graph (a minimum of 70% residues overlap is still required). This expanded combination is then merged into the final query sequence consensus annotation (**Figure 7.D**). Redundant (i.e., repeated identifiers or functional descriptions) or poor quality information (e.g., "hypothetical protein") is removed from the consensus annotation.

Establishing a test environment

For annotation quality benchmarking, we evaluate each annotation produced by Mantis and check whether it agrees (database IDs intersection) with the respective reference annotation, creating a confusion matrix. We created two main types of test samples, the first consisting exclusively of curated UniProt [80] protein entries (and the respective annotations) which were then split by date of creation (2010-2020, 2015-2020, 2018-2020, 2020). The second type consisting of organism-specific UniProt protein entries, with a mix of curated and automatically generated annotations. Each sequence's reference annotation consists of the UniProt protein

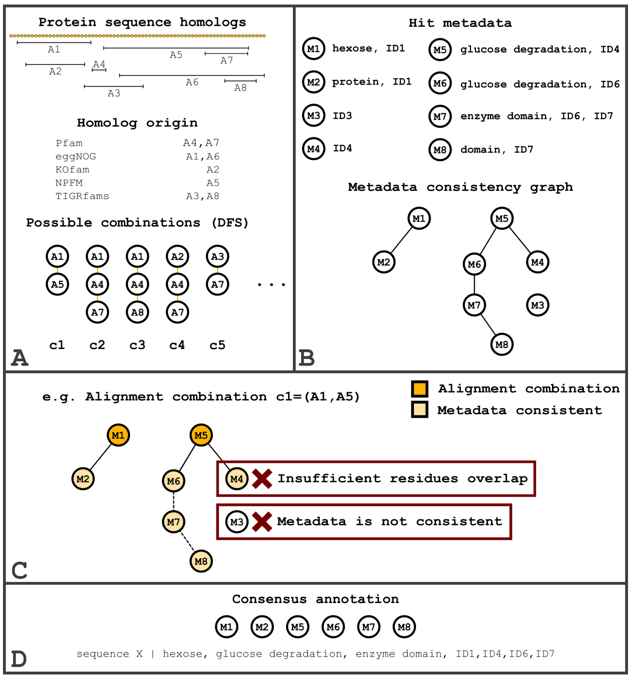

Figure 7. Inter-HMMs hit processing steps. Inter-HMMs hits processing starts by pooling all hits [A1,AN] together (regardless of the reference data source), and generating all the possible (non-overlapping coordinates) combinations [c1,cN] (A). A metadata consistency graph (B) is also built by connecting all nodes [M1,MN] that have intersecting IDs or highly similar descriptions (e.g., A1's metadata M1 is consistent with A2's metadata M2 – shared ID1, and A5's metadata M5 is consistent with A6's metadata M6 – similar description 'glucose degradation'). With this metadata consistency graph, the hit consistency HCN score of each combination is calculated. For c1, for example, a sub-graph containing M1, M5 and all directly connected nodes (only M2 and M6, but not M4, since it has insufficient residues overlap – A4) would be created. The number of nodes in this sub-graph would then be divided by the total number of nodes in the original graph, therefore c1 would have an HCN of $\frac{2+2}{8} = 0.5$. The remaining parameters would then be calculated and the best combination, according to equation 2, would be selected. Finally, if, for example, the best combination is c1, then this combination is expanded by merging all nodes directly or indirectly connected to M1 and M5 in the metadata consistency graph (C) and with sufficient residues overlap (i.e., M2, M6, M7, M8). The expanded combination is then merged into the final consensus annotation (D).

function annotations. Each sequence reference annotation and the respective PFA tool's annotation is composed of a set of identifiers (if available: enzyme ECs, Gene ontology (GO) IDs, eggNOG IDs, KEGG orthology IDs, Pfam IDs, and TIGRfam IDs) and functional descriptions. During the benchmark process, each sequence's reference annotation (e.g., "glucose degradation ID1") is compared against the PFA tool (i.e., Mantis, eggNOG-mapper, and Prokka) annotation (e.g., "degrades glucose ID1"). This comparison entails checking whether any of the database IDs present in the reference annotation (i.e., ID1) are also present in the PFA tool annotation (i.e., ID1); if they are, we consider this annotation to be the same. This has some significant limitations: (i) the functional description is the same but the corresponding set of identifiers is not; and (ii) when annotating multiple regions of the protein (which is the case when using Mantis' DFS and heuristic algorithms), it is possible that only one of the annotated regions has IDs that intersect with the respective sequence reference annotation. Unfortunately, due to the different resolutions of the reference HMMs, it is not always possible to understand whether an annotation refers to a specific domain or a partial whole-sequence hit. While a domain-centric benchmark would be feasible for Pfam, the same is not true for the remaining reference HMMs with broader resolutions (e.g., TIGRFams provides general functional annotations). However, as we have previously shown, even when using the BPO algorithm, Mantis has shown to output almost equally high F1 scores. Despite these limitations, since whole-sequence reference annotations contain comprehensive cross-linking with other databases, it provides clear benefits: (i) it fits better for the wide-ranging scopes of the reference data sources, and (ii) allows for a more fair benchmark of the different PFA tools that may use different reference data sources (and thus output annotations with different database IDs). This method then allows for the construction of a confusion matrix, where each pairwise whole sequence annotation comparison (PFA tool/reference annotation) corresponds to a single class. **True-Positives** (TP) occur when the PFA tool generated annotation and the reference annotation share one or more database IDs (e.g., Pfam ID), **False-Positives** (FP) when no database IDs are shared. **False-Negatives** (FN) when the PFA tool does not annotate a protein sequence, but a reference annotation is available, and **True-Negatives** (TN) when the PFA tool does not annotate a protein sequence, and no reference annotation is available. The functional text descriptions are not taken into account during the benchmark, therefore if an annotation has no IDs, we simply consider there is no annotation. Protein sequences annotated with the descriptions "unknown function", "uncharacterized protein", "hypothetical protein" or with Pfam's "domain-unknown-function"/DUF IDs are not taken into account during benchmarking (for reference and PFA tools annotations). In addition, it is also possible that the reference or PFA tool do not have an annotation for a certain sequence. In any of the these three scenarios, if the PFA tool manages to annotate the sequence, this case is classified as potentially new annotation (PNA). Since no ground-truth exists in these scenarios, PNAs are excluded from the confusion matrix classes (not used during any performance metrics) and are only used to calculate the annotation coverage. PNAs can potentially provide novel insight into protein sequences without any previous annotation. Since, by default, most sequences used during benchmarking will have an annotation, TNs, ergo any metrics using TNs (e.g., specificity), are irrelevant.

Annotation coverage is defined here as the number of annotations produced by the PFA tool divided by the total number of protein sequences in a sample $Total_{seqs}$. $Total_{seqs}$ includes sequences with and without a reference annotation (since not all sequences have a reference annotation), the total number of the PFA tool annotations includes TPs, FPs, and PNAs. Annotation coverage is calculated with $\frac{TP+FP+PNA}{Total_{seqs}}$. Numerous metrics can be calculated from the various confusion matrix categories, we considered precision and recall/sensitivity to be among the most important. **Precision** is defined as $\frac{TP}{TP+FP}$ and corresponds to the number of correctly annotated protein sequences, out of all the protein sequences the PFA tool managed to annotate. **Recall** is defined as $\frac{TP}{TP+FN}$ and corresponds to the number of correctly annotated protein sequences, out of all the protein sequences that we know the function of (i.e., protein sequences that have a reference annotation). Both are equally important, a tool with low precision will incorrectly annotate protein sequences, whereas a tool with low recall will not produce sufficient annotations. A way to converge both scores into one is to use the **F1 score**, which is defined as $2 \times \frac{Precision \times Recall}{Precision+Recall}$. Unless otherwise stated, values shown in this paper are shown as absolute values ranging from 0 to 1.

Finally, we benchmarked Mantis against two other PFA tools - eggNOG-mapper and Prokka. For homology search, Mantis uses HMMER [24], for eggNOG-mapper we used the Diamond-based [23] search (as suggested by the authors), and Prokka uses BLAST and HMMER.

All tests ran on an HPC with Dell C6320, 2 * Intel Xeon E5-2680 v4 @ 2.4 GHz [81], each core had 4GB of RAM. Unless specified, all tests ran with 25 cores and 100GB RAM (actual Mantis minimum hardware requirements are much lower). In addition, the same methodology and nomenclature apply to any other benchmarked tools described in this paper. Mantis used HMMER v3.2.1. The local version of eggNOG-mapper used was v2.0.6 with database v5.0.1 found at `https://github.com/eggnogdb/eggnog-mapper/commit/41ec3566ab00fd437f905dfde592c553632a9eae`. The local version of Prokka used was v1.14.6 found at `https://github.com/tseemann/prokka/releases/tag/v1.14.6`.

For details on execution commands please see the supplemental PDF.

Sample selection

As an initial testing dataset we started by downloading all the curated Uni-Prot [80] (i.e., Swiss-Prot) protein entries created after 2010 (until 2020/04/14), along with their respective sequences, annotations, and annotations scores. We then split these entries by date, 2010-2020, 2015-2020,2018-2020, and 2020 only. For genomic sample benchmarking we selected organisms widely used in microbial community standards. The respective genomes, proteomes, and reference annotations were then downloaded from Uniprot on 2020/05/26 (supplemental **Table 5**). These samples were also used for comparing Mantis to eggNOG-mapper and Prokka.

Testing different e-value thresholds

Different e-value thresholds were tested: $1e^{-3}$, $1e^{-6}$,$1e^{-9}$,$1e^{-12}$,$1e^{-15}$,$1e^{-18}$,$1e^{-21}$,$1e^{-24}$,$1e^{-27}$,$1e^{-30}$, and a dynamic threshold. The dynamic threshold was set according to the query sequence length, which was previously shown to provide better results with BLAST [34]. For the dynamic threshold, for sequences with less than 150 amino acids, the e-value threshold was set to $1e^{-10}$, if above 150 and below 250, $1e^{-\frac{sequence_{length}}{10}}$, and if above 250, $1e^{-25}$. The UniProt 2010-2020 sample was then annotated with all the different e-value thresholds, and each output was compared to the reference annotations.

Testing hit processing algorithms

In order to understand whether the different hit processing algorithms resulted in statistically significant differences in F1 scores, we created 5000 randomized synthetic samples with 5000 sequences each, which were randomly selected from the 2010-2020 UniProt sample. Per algorithm, we compared the Mantis annotations of each subset to the reference annotations (to allow for pairwise comparison of each algorithm, the same subsets were used in all algorithms). This resulted in a list of confusion matrices (5000 per algorithm), from which we calculated the F1 score. We applied the Wilcoxon signed-rank test, with the H_0: no differences in F1 score between the tested algorithms. As a non-parametric test, this test makes no assumptions on the distribution of the data. A pairwise comparison was done between DFS and the other algorithms: (i) DFS and heuristic, and (ii) DFS and BPO.

Availability of source code and requirements

- Project name: Mantis
- Project home page: `https://github.com/PedroMTQ/mantis`
- Operating system: Linux
- Programming language: Python
- Other requirements: Python 3+, HMMER 3+, and several Python packages (please see the provided environment for a full list)
- License: MIT license at `https://github.com/PedroMTQ/mantis/blob/master/LICENSE`
- RRID: SCR_021001
- Biotools ID: mantis_pfa

Availability of supporting data and materials

The data and code supporting the results of this article are available at `https://git-r3lab.uni.lu/pedro.queiros/mantis_supplements`. The supplemental pdf "supplements.pdf" contains: (i) discussion on how the e-value threshold may change Mantis' output, (ii) execution commands, and (iii) information on how the similarity analysis was performed. The *supplements.xlsx* file contains all tables referenced in this article. The first sheet *ToC* contains the table of contents.

Declarations

List of abbreviations

BPO - best prediction only
CE - combination e-value
DFS - depth first search
EC - enzyme commission
FP - false-positives
FN - false-negatives
GFS - glacier-fed stream sediment
GO - gene ontology
HC - average hit coverage
HCN - hit consistency
HMM - hidden Markov Models
HMMW - average reference HMMs weight
HPC - high-performance computing
ID - database identifier
KO - KEGG orthology
MQ - metadata quality
NLP - natural language processing
NPFM - NCBI protein family models
PFA - protein function annotation
PNA - potentially new annotation
RAM - random access memory
TC - total coverage
TN - true-negatives
TP - true-positives
TSA - taxa-specific annotation
TSHMM - taxa-specific HMM

Competing Interests

The authors declare that they have no competing interests.

Funding

Supported by the Luxembourg National Research Fund PRIDE17/11823097.

Author's Contributions

Author contributions according to the contributor roles taxonomy CRediT was as follows: Conceptualization: P.Q. and P.M.; Data curation: P.Q.; Formal Analysis: P.Q.; Funding acquisition: P.W. and P.M.; Investigation: P.Q.; Methodology: P.Q. and P.M.; Project administration: P.Q. and P.M.; Resources: P.Q.; Software: P.Q.; Supervision: P.M. and P.W.; Validation: P.Q. (lead), F.D., and O.H.; Visualization: P.Q.; Writing – original draft: P.Q. (lead), and P.M.; Writing – review & editing: P.Q., P.M., F.D. , O.H., and P.W.. All authors proof-read and approved of the content in this research paper.

Acknowledgements

The experiments presented in this paper were carried out using the HPC facilities of the University of Luxembourg [81]. P.W. acknowledges the European Research Council (ERC-CoG 863664). We would like to thank Tomila Litvishko for proofreading this research paper. We would like to acknowledge all the creators of the reference data and software used by Mantis, building upon the complementary knowledge of others truly moves the field forward.

References

1. Segata N, Boernigen D, Tickle TL, Morgan XC, Garrett WS, Huttenhower C. Computational meta'omics for microbial community studies. Molecular Systems Biology 2013;9.
2. Muller E, Glaab E, May P, Vlassis N, Wilmes P. Condensing the omics fog of microbial communities. Trends in microbiology 2013 06;21.
3. Whisstock JC, Lesk AM. Prediction of protein function from protein sequence and structure. Quarterly Reviews of Biophysics 2003;36(3):307–340.
4. Arias C, Weisburd B, Stern-Ginossar N, Mercier A, Madrid AS, Bellare P, et al. KSHV 2.0: A Comprehensive Annotation of the Kaposi's Sarcoma-Associated Herpesvirus Genome Using Next-Generation Sequencing Reveals Novel Genomic and Functional Features. PLOS Pathogens 2014;10(1):e1003847.
5. Chapel A, Kieffer-Jaquinod S, Sagné C, Verdon Q, Ivaldi C, Mellal M, et al. An Extended Proteome Map of the Lysosomal Membrane Reveals Novel Potential Transporters. Molecular & Cellular Proteomics 2013;12(6):1572–1588.
6. Iorizzo M, Senalik DA, Grzebelus D, Bowman M, Cavagnaro PF, Matvienko M, et al. De novo assembly and

characterization of the carrot transcriptome reveals novel genes, new markers, and genetic diversity. BMC Genomics 2011;12(1):389.

7. Heintz-Buschart A, May P, Laczny CC, Lebrun LA, Bellora C, Krishna A, et al. Integrated multi-omics of the human gut microbiome in a case study of familial type 1 diabetes. Nature Microbiology 2016;2(1):1–13. Number: 1 Publisher: Nature Publishing Group.
8. Mason OU, Scott NM, Gonzalez A, Robbins-Pianka A, Bælum J, Kimbrel J, et al. Metagenomics reveals sediment microbial community response to Deepwater Horizon oil spill. The ISME Journal 2014;8(7):1464–1475.
9. Pasolli E, Asnicar F, Manara S, Zolfo M, Karcher N, Armanini F, et al. Extensive Unexplored Human Microbiome Diversity Revealed by Over 150,000 Genomes from Metagenomes Spanning Age, Geography, and Lifestyle. Cell 2019;176(3):649–662.e20.
10. Sureyya Rifaioglu A, Doğan T, Jesus Martin M, Cetin-Atalay R, Atalay V. DEEPred: Automated Protein Function Prediction with Multi-task Feed-forward Deep Neural Networks. Scientific Reports 2019;9(1):7344.
11. Vazquez A, Flammini A, Maritan A, Vespignani A. Global protein function prediction from protein-protein interaction networks. Nature Biotechnology 2003;21(6):697–700.
12. Borgwardt KM, Ong CS, Schönauer S, Vishwanathan SVN, Smola AJ, Kriegel HP. Protein function prediction via graph kernels. Bioinformatics 2005;21:i47–i56.
13. Steinegger M, Meier M, Mirdita M, Vöhringer H, Haunsberger SJ, Söding J. HH-suite3 for fast remote homology detection and deep protein annotation. BMC Bioinformatics 2019;20(1):473.
14. Seemann T. Prokka: rapid prokaryotic genome annotation. Bioinformatics 2014;30(14):2068–2069.
15. Huerta-Cepas J, Forslund K, Coelho LP, Szklarczyk D, Jensen LJ, von Mering C, et al. Fast Genome-Wide Functional Annotation through Orthology Assignment by eggNOG-Mapper. Molecular Biology and Evolution 2017;34(8):2115–2122.
16. Aziz RK, Bartels D, Best AA, DeJongh M, Disz T, Edwards RA, et al. The RAST Server: Rapid Annotations using Subsystems Technology. BMC Genomics 2008;9(1):75.
17. Ryu JY, Kim HU, Lee SY. Deep learning enables high-quality and high-throughput prediction of enzyme commission numbers. Proceedings of the National Academy of Sciences 2019;116(28):13996–14001.
18. Zhao B, Hu S, Li X, Zhang F, Tian Q, Ni W. An efficient method for protein function annotation based on multilayer protein networks. Human Genomics 2016;10.
19. Szklarczyk D, Gable AL, Lyon D, Junge A, Wyder S, Huerta-Cepas J, et al. STRING v11: protein-protein association networks with increased coverage, supporting functional discovery in genome-wide experimental datasets. Nucleic Acids Research 2019;47:D607–D613.
20. Deng L, Zhong G, Liu C, Luo J, Liu H. MADOKA: an ultra-fast approach for large-scale protein structure similarity searching. BMC Bioinformatics 2019;20(19):662.
21. Overbeek R, Begley T, Butler RM, Choudhuri JV, Chuang HY, Cohoon M, et al. The subsystems approach to genome annotation and its use in the project to annotate 1000 genomes. Nucleic Acids Research 2005;33(17):5691–5702.
22. Altschul SF, Gish W, Miller W, Myers EW, Lipman DJ. Basic local alignment search tool. Journal of Molecular Biology 1990;215(3):403–410.
23. Buchfink B, Xie C, Huson DH. Fast and sensitive protein alignment using DIAMOND. Nature Methods 2015;12(1):59–60.
24. Roberts Eddy S, HMMER; 2020.
25. Jones P, Binns D, Chang HY, Fraser M, Li W, McAnulla C, et al. InterProScan 5: genome-scale protein function classification. Bioinformatics 2014;30(9):1236–1240.
26. Lohse M, Nagel A, Herter T, May P, Schroda M, Zrenner R, et al. Mercator: a fast and simple web server for genome scale functional annotation of plant sequence data. Plant, Cell & Environment 2014;37(5):1250–1258.
27. Wu S, Zhu Z, Fu L, Niu B, Li W. WebMGA: a customizable web server for fast metagenomic sequence analysis. BMC genomics 2011;12.
28. Mitchell AL, Almeida A, Beracochea M, Boland M, Burgin J, Cochrane G, et al. MGnify: the microbiome analysis resource in 2020. Nucleic Acids Research 2020;48:D570–D578.
29. Keegan KP, Glass EM, Meyer F. MG-RAST, a Metagenomics Service for Analysis of Microbial Community Structure and Function. Methods in Molecular Biology (Clifton, NJ) 2016;1399:207–233.
30. Pfeiffer F, Oesterhelt D. A Manual Curation Strategy to Improve Genome Annotation: Application to a Set of Haloarchael Genomes. Life 2015;5(2):1427–1444.
31. Yeats C, Redfern OC, Orengo C. A fast and automated solution for accurately resolving protein domain architectures. Bioinformatics 2010;26(6):745–751.
32. Ekman D, Bjorklund AK, Frey-Skott J, Elofsson A. Multi-domain Proteins in the Three Kingdoms of Life: Orphan Domains and Other Unassigned Regions. Journal of Molecular Biology 2005;348(1):231–243.
33. Lees JG, Lee D, Studer RA, Dawson NL, Sillitoe I, Das S, et al. Gene3D: Multi-domain annotations for protein sequence and comparative genome analysis. Nucleic Acids Research 2014;42:D240–D245.
34. Treiber ML, Taft DH, Korf I, Mills DA, Lemay DG. Pre- and post-sequencing recommendations for functional annotation of human fecal metagenomes. BMC Bioinformatics 2020;21(1):74.
35. Schnoes AM, Brown SD, Dodevski I, Babbitt PC. Annotation Error in Public Databases: Misannotation of Molecular Function in Enzyme Superfamilies. PLoS Computational Biology 2009;5(12).
36. Friedberg I. Automated protein function prediction—the genomic challenge. Briefings in Bioinformatics 2006;7(3):225–242.
37. Araujo FA, Barh D, Silva A, Guimarães L, Ramos RTJ. GO FEAT: a rapid web-based functional annotation tool for genomic and transcriptomic data. Scientific Reports 2018;8(1):1794.
38. Klimke W, O'Donovan C, White O, Brister JR, Clark K, Fedorov B, et al. Solving the Problem: Genome Annotation Standards before the Data Deluge. Standards in Genomic Sciences 2011;5(1):168–193.
39. Standardizing data. Nature Cell Biology 2008;10(10):1123–1124.
40. Gaikwad SV, Chaugule A, Patil P. Text mining methods and techniques. International Journal of Computer Applications 2014;85(17).
41. Wang S, Ma J, Yu MK, Zheng F, Huang EW, Han J, et al. Annotating gene sets by mining large literature collections with protein networks. Pacific Symposium on Biocomputing Pacific Symposium on Biocomputing 2018;23:602–613.
42. Pesquita C, Faria D, Falcão AO, Lord P, Couto FM. Semantic similarity in biomedical ontologies. PLoS computational biology 2009;5(7):e1000443.
43. Zeng Z, Shi H, Wu Y, Hong Z. Survey of Natural Language Processing Techniques in Bioinformatics. Computational and Mathematical Methods in Medicine 2015;2015:674296.
44. Slater LT, Bradlow W, Ball S, Hoehndorf R, Gkoutos GV. Improved characterisation of clinical text through ontology-based vocabulary expansion. bioRxiv 2020;p.

2020.07.10.197541.
45. Huang CC, Lu Z. Community challenges in biomedical text mining over 10 years: success, failure and the future. Briefings in Bioinformatics 2016;17(1):132–144.
46. Benabderrahmane S, Smail-Tabbone M, Poch O, Napoli A, Devignes MD. IntelliGO: a new vector-based semantic similarity measure including annotation origin. BMC Bioinformatics 2010;11:588.
47. Peng J, Uygun S, Kim T, Wang Y, Rhee SY, Chen J. Measuring semantic similarities by combining gene ontology annotations and gene co-function networks. BMC bioinformatics 2015;16.
48. Liu M, Thomas PD. GO functional similarity clustering depends on similarity measure, clustering method, and annotation completeness. BMC bioinformatics 2019;20(1):155.
49. Daraselia N, Yuryev A, Egorov S, Mazo I, Ispolatov I. Automatic extraction of gene ontology annotation and its correlation with clusters in protein networks. BMC bioinformatics 2007;8:243.
50. Ehsani R, Drabløs F. TopoICSim: a new semantic similarity measure based on gene ontology. BMC bioinformatics 2016;17(1):296.
51. Kramer M, Dutkowski J, Yu M, Bafna V, Ideker T. Inferring gene ontologies from pairwise similarity data. Bioinformatics (Oxford, England) 2014;30(12):i34–42.
52. Promponas VJ, Iliopoulos I, Ouzounis CA. Annotation inconsistencies beyond sequence similarity-based function prediction – phylogeny and genome structure. Standards in Genomic Sciences 2015;10.
53. Ellens KW, Christian N, Singh C, Satagopam VP, May P, Linster CL. Confronting the catalytic dark matter encoded by sequenced genomes. Nucleic Acids Research 2017;45(20):11495–11514.
54. Aramaki T, Blanc-Mathieu R, Endo H, Ohkubo K, Kanehisa M, Goto S, et al. KofamKOALA: KEGG Ortholog assignment based on profile HMM and adaptive score threshold. Bioinformatics 2020;36(7):2251–2252.
55. El-Gebali S, Mistry J, Bateman A, Eddy SR, Luciani A, Potter SC, et al. The Pfam protein families database in 2019. Nucleic Acids Research 2019;47:D427–D432.
56. Huerta-Cepas J, Szklarczyk D, Heller D, Hernández-Plaza A, Forslund SK, Cook H, et al. eggNOG 5.0: a hierarchical, functionally and phylogenetically annotated orthology resource based on 5090 organisms and 2502 viruses. Nucleic Acids Research 2019;47:D309–D314.
57. Lu S, Wang J, Chitsaz F, Derbyshire MK, Geer RC, Gonzales NR, et al. CDD/SPARCLE: the conserved domain database in 2020. Nucleic acids research 2020;48(D1):D265–D268.
58. Haft DH, Selengut JD, Richter RA, Harkins D, Basu MK, Beck E. TIGRFAMs and Genome Properties in 2013. Nucleic Acids Research 2013;41:D387–D395.
59. Queirós P, Mantis - Wiki. GitHub; 2020. https://github.com/PedroMTQ/mantis/wiki.
60. Hyatt D, Chen GL, LoCascio PF, Land ML, Larimer FW, Hauser LJ. Prodigal: prokaryotic gene recognition and translation initiation site identification. BMC Bioinformatics 2010;11:119.
61. Delogu F, fdelogu/SEM1b-Multiomics; 2019.
62. Kunath BJ, Delogu F, Naas AE, Arntzen M, Eijsink VGH, Henrissat B, et al. From proteins to polysaccharides: lifestyle and genetic evolution of Coprothermobacter proteolyticus. The ISME Journal 2019;13(3):603–617.
63. Busi SB, Pramateftaki P, Brandani J, Fodelianakis S, Peter H, Halder R, et al. Optimised biomolecular extraction for metagenomic analysis of microbial biofilms from high-mountain streams. PeerJ 2020 Oct;8:e9973. https://doi.org/10.7717/peerj.9973.
64. Sunagawa S, Coelho LP, Chaffron S, Kultima JR, Labadie K, Salazar G, et al. Structure and function of the global ocean microbiome. Science 2015;348(6237). https://science.sciencemag.org/content/348/6237/1261359.
65. Sigrist CJA, de Castro E, Cerutti L, Cuche BA, Hulo N, Bridge A, et al. New and continuing developments at PROSITE. Nucleic Acids Research 2013;41:D344–347.
66. Mooney MA, Nigg JT, McWeeney SK, Wilmot B. Functional and Genomic Context in Pathway Analysis of GWAS Data. Trends in genetics : TIG 2014;30(9):390–400.
67. Mavromatis K, Chu K, Ivanova N, Hooper SD, Markowitz VM, Kyrpides NC. Gene Context Analysis in the Integrated Microbial Genomes (IMG) Data Management System. PLOS ONE 2009;4(11):e7979.
68. Hannigan GD, Prihoda D, Palicka A, Soukup J, Klempir O, Rampula L, et al. A deep learning genome-mining strategy for biosynthetic gene cluster prediction. Nucleic Acids Research 2019 08;47(18):e110–e110.
69. Parks DH, Chuvochina M, Waite DW, Rinke C, Skarshewski A, Chaumeil PA, et al. A standardized bacterial taxonomy based on genome phylogeny substantially revises the tree of life. Nature Biotechnology 2018;36(10):996–1004.
70. Parks DH, Chuvochina M, Chaumeil PA, Rinke C, Mussig AJ, Hugenholtz P. A complete domain-to-species taxonomy for Bacteria and Archaea. Nature Biotechnology 2020;38(9):1079–1086.
71. Buell R, Deutschbauer A, Adin D, Ronning C, Breaking the Bottleneck of Genomes: Understanding Gene Function Across Taxa; 2018.
72. Sunagawa S, Coelho LP, Chaffron S, Kultima JR, Labadie K, Salazar G, et al. Structure and function of the global ocean microbiome. Science 2015;348(6237). Publisher: American Association for the Advancement of Science Section: Research Article.
73. Mangul S, Mosqueiro T, Abdill RJ, Duong D, Mitchell K, Sarwal V, et al. Challenges and recommendations to improve the installability and archival stability of omics computational tools. PLOS Biology 2019 06;17(6):1–16. https://doi.org/10.1371/journal.pbio.3000333.
74. Queirós P, Mantis - configuration file. GitHub; 2020. https://github.com/PedroMTQ/mantis/blob/master/MANTIS.config.
75. Queirós P, Mantis - Output files. GitHub; 2020. https://github.com/PedroMTQ/mantis/wiki/Output.
76. Queirós P, Mantis - Custom HMMs. GitHub; 2020. https://github.com/PedroMTQ/mantis/wiki/Configuration#custom-hmms.
77. Queirós P, Mantis - Custom HMMs weights. GitHub; 2020. https://github.com/PedroMTQ/mantis/wiki/Configuration#setting-hmms-weight.
78. Kaur N, Garg D. Analysis of the Depth First Search Algorithms. Data mining and knowledge engineering 2012;4:37–41.
79. Behnel S, Bradshaw R, Citro C, Dalcin L, Seljebotn DS, Smith K. Cython: The Best of Both Worlds. Computing in Science Engineering 2011;13(2):31–39.
80. UniProt: a worldwide hub of protein knowledge. Nucleic Acids Research 2019;47:D506–D515.
81. Varrette S, Bouvry P, Cartiaux H, Georgatos F. Management of an Academic HPC Cluster: The UL Experience 2014;https://hpc.uni.lu.

Supplemental tables

Click here to access/download
Supplementary Material
supplements.xlsx

Supplemental PDF

Click here to access/download
Supplementary Material
supplemental.pdf

for reviewers: Version comparison

Click here to access/download

Supplementary Material

version_comparison.pdf

for reviewers: Manuscript PDF with lines

Click here to access/download
Supplementary Material
manuscript_lines.pdf

reviewer response

Click here to access/download
Supplementary Material
reviewer_response.pdf

Author information

Click here to access/download
Supplementary Material
author_information.txt

Cover letter

Click here to access/download;Personal Cover;cover_letter.pdf

UNIVERSITÉ DU LUXEMBOURG

LUXEMBOURG CENTRE FOR SYSTEMS BIOMEDICINE, UNIVERSITY OF LUXEMBOURG

E-MAIL: pdqueiros@gmail.com

Dr. Scott Edmunds, PhD
Editor-in-Chief
GigaScience Journal

22nd March, 2021

Dear Dr. Edmunds,

We would like to thank the editor for giving us the opportunity to revise and resubmit our manuscript entitled "**Mantis: flexible and consensus-driven genome annotation**". We would also like to thank the editor and reviewers for the very in-depth review.

In response to the suggestions provided and concerns raised, we have extensively revised our manuscript. In particular, we have addressed the main issues raised by reviewers: (i) methodology of our consensus-driven approach, (ii) method used for setting the text similarity threshold, and (iii) benchmarking methodology. The aforementioned and all other points brought up by the reviewers have been compiled and answered in a separate PDF file. For your convenience, we have also attached a comparison between the old and new manuscripts, as well as a PDF version of our manuscript with lines.

Please do not hesitate to contact me if any further information is required by either the Editorial Board or the reviewers.

On behalf of the authors,

Pedro Queirós
