# Supplementary material for: Mantis: flexible and consensus-driven genome annotation
Source: Gigascience. 2021 Jun 2;10(6):giab042. doi: 10.1093/gigascience/giab042 (PMC8170692; doi:10.1093/gigascience/giab042)
Supplement: giab042_GIGA-D-20-00320_Revision_4 [file giab042_giga-d-20-00320_revision_4.pdf]

GigaScience

Mantis: flexible and consensus-driven genome annotation

--Manuscript Draft--

**Manuscript Number:**	GIGA-D-20-00320R4
**Full Title:**	Mantis: flexible and consensus-driven genome annotation
**Article Type:**	Technical Note
**Funding Information:**	Fonds National de la Recherche Luxembourg (PRIDE17/11823097) — Dr Paul Wilmes
**Abstract:**	Background<br><br>The past decades have seen a rapid development of the (meta-)omics fields, producing an unprecedented amount of high-resolution and high-fidelity data. Through the use of these datasets we can infer the role of previously functionally unannotated proteins from single organisms and consortia. In this context, protein function annotation can be described as the identification of regions of interest (i.e., domains) in protein sequences and the assignment of biological functions. Despite the existence of numerous tools, some challenges remain, specifically in terms of speed, flexibility, and reproducibility. In the era of big data analysis, it is also increasingly important to cease limiting our findings to a single reference, coalescing knowledge from different data sources, and thus overcoming some limitations in overly relying on computationally generated data from single sources.<br>Results<br><br>We implemented a protein annotation tool - Mantis, which uses database identifiers intersection and text mining to integrate knowledge from multiple reference data sources into a single consensus-driven output. Mantis is flexible, allowing for the customization of reference data and execution parameters, and is reproducible across different research goals and user environments. We implemented a depth-first search algorithm for domain-specific annotation, which significantly improved annotation performance compared to sequence-wide annotation. The parallelized implementation of Mantis results in short runtimes while also outputting high coverage and high-quality protein function annotations.<br><br><br>Conclusions<br>Mantis is a protein function annotation tool that produces high-quality consensus-driven protein annotations. It is easy to set up, customize, and use, scaling from single genomes to large metagenomes.<br><br>Mantis is available under the MIT license available at https://github.com/PedroMTQ/mantis.
**Corresponding Author:**	Pedro Queirós<br>University of Luxembourg<br>Esch-sur-Alzette, LUXEMBOURG
**Corresponding Author Secondary Information:**	
**Corresponding Author's Institution:**	University of Luxembourg
**Corresponding Author's Secondary Institution:**	
**First Author:**	Pedro Queirós
**First Author Secondary Information:**	
**Order of Authors:**	Pedro Queirós
	Francesco Delogu

Powered by Editorial Manager® and ProduXion Manager® from Aries Systems Corporation

	Oskar Hickl
	Patrick May
	Paul Wilmes
**Order of Authors Secondary Information:**	
**Response to Reviewers:**	Dear Editor,<br><br>I've fixed the issue with the doi as requested.<br><br>Regards,<br>Pedro
**Additional Information:**	
**Question**	**Response**
Are you submitting this manuscript to a special series or article collection?	No
**Experimental design and statistics**<br><br>Full details of the experimental design and statistical methods used should be given in the Methods section, as detailed in our Minimum Standards Reporting Checklist. Information essential to interpreting the data presented should be made available in the figure legends.<br><br>Have you included all the information requested in your manuscript?	Yes
**Resources**<br><br>A description of all resources used, including antibodies, cell lines, animals and software tools, with enough information to allow them to be uniquely identified, should be included in the Methods section. Authors are strongly encouraged to cite Research Resource Identifiers (RRIDs) for antibodies, model organisms and tools, where possible.<br><br>Have you included the information requested as detailed in our Minimum Standards Reporting Checklist?	Yes
**Availability of data and materials**	Yes

All datasets and code on which the conclusions of the paper rely must be either included in your submission or deposited in publicly available repositories (where available and ethically appropriate), referencing such data using a unique identifier in the references and in the “Availability of Data and Materials” section of your manuscript.<br><br>Have you have met the above requirement as detailed in our Minimum Standards Reporting Checklist?	

Manuscript Click here to access/download;Manuscript;main.tex

Placeholder for OUP logo oup.pdf

Placeholder for journal logo gigascience-logo.pdf

GigaScience, 2017, 1–14

doi: xx.xxxx/xxxx
Manuscript in Preparation
Paper

PAPER

Mantis: flexible and consensus-driven genome annotation

Pedro Queirós,[1,*], Francesco Delogu,[1], Oskar Hickl,[2], Patrick May,[2,†] and Paul Wilmes,[1,‡]

[1]Systems Ecology, Luxembourg Centre for Systems Biomedicine, University of Luxembourg and [2]Bioinformatics Core, Luxembourg Centre for Systems Biomedicine, University of Luxembourg

[*]pedro.queiros@uni.lu
[†]patrick.may@uni.lu
[‡]paul.wilmes@uni.lu

Abstract

Background The past decades have seen a rapid development of the (meta-)omics fields, producing an unprecedented amount of high-resolution and high-fidelity data. Through the use of these datasets we can infer the role of previously functionally unannotated proteins from single organisms and consortia. In this context, protein function annotation can be described as the identification of regions of interest (i.e., domains) in protein sequences and the assignment of biological functions. Despite the existence of numerous tools, some challenges remain, specifically in terms of speed, flexibility, and reproducibility. In the era of big data analysis, it is also increasingly important to cease limiting our findings to a single reference, coalescing knowledge from different data sources, and thus overcoming some limitations in overly relying on computationally generated data from single sources.
Results We implemented a protein annotation tool - Mantis, which uses database identifiers intersection and text mining to integrate knowledge from multiple reference data sources into a single consensus-driven output. Mantis is flexible, allowing for the customization of reference data and execution parameters, and is reproducible across different research goals and user environments. We implemented a depth-first search algorithm for domain-specific annotation, which significantly improved annotation performance compared to sequence-wide annotation. The parallelized implementation of Mantis results in short runtimes while also outputting high coverage and high-quality protein function annotations.
Conclusions Mantis is a protein function annotation tool that produces high-quality consensus-driven protein annotations. It is easy to set up, customize, and use, scaling from single genomes to large metagenomes. Mantis is available under the MIT license available at **https://github.com/PedroMTQ/mantis**.

Key words: bioinformatics; consensus; homology; HMM; protein function annotation;

Background

On a cellular scale, life is, in essence, the activity and the interaction of a plethora of different molecules, among which proteins are responsible for the vast majority of processes. A primary task in understanding how biology works is to resolve its actors properly (e.g., the proteins) and place them into context. The past decades have seen the development of the (meta-)omics fields, unlocking an unprecedented amount of data and deepening our understanding in several fields of biology [1, 2]. Alongside the evolution of the technologies and the increase in data volume, the identification of proteins transitioned from purely experimental techniques (e.g., chemical essays and spectroscopy) toward the computational-based sequence analysis thanks to the discovery of the relationship between

Compiled on: May 13, 2021.
Draft manuscript prepared by the author.

conservation of proteins' functions and sequences [3]. Therefore, the current challenges are to make use of the vast number of protein sequences and annotations available and to link new protein sequences to the previously established knowledge. High-throughput methods, such as next-generation sequencing, are able to produce a large amount of data which then needs to be analysed and interpreted. One of the ways to make sense of this data is through protein function annotation (PFA), which is, in the context of this paper, the identification of regions of interest (i.e., domains) in a sequence and assignment of biological function(s) to these regions. This strategy has proven effective in the study of single organisms as well as consortia [4, 5, 6, 7, 8, 9]. Function prediction is based on reference data, i.e., transferring the function from protein X to the unknown protein Y if they are highly similar [3]. Different approaches may be used, the most common being the comparison of an unknown protein sequence to reference data composed of well-studied and functionally annotated proteins (homology-based methods) [10, 11, 12, 13, 14, 15, 16]. Other methods may infer function through the use of machine learning [10, 17], protein networks [18, 19], protein structure [20], or genomics context-based techniques [21], but these will not be covered in this paper. For sequence alignment, BLAST [22] or Diamond [23] are commonly used, whereas, for hidden Markov models (HMM) profiles, HMMER [24] is most widely used. In PFA, these tools are often integrated into larger pipelines to provide enhanced output interpretability, workflow automation, and parallelization [14, 15, 16, 25]. Some PFA tools target specific taxa [26], others are designed with large-scale omics analysis in mind [27, 28, 29]; indeed, each PFA tool is designed to cater to its niche research topic. While experimental validation remains the gold standard, PFA, despite its many shortcomings [30], is an increasingly valuable strategy that aims to tackle the progressively more difficult task of making sense of the large quantities of data being continuously generated.

The most common method of processing candidate annotations (i.e., sequences or HMM profiles that are highly similar to the query sequence) is done by capturing only the most significant candidate ("best prediction only", hereinafter called the **BPO** algorithm). This PFA approach works well for single-domain proteins, but multi-domain proteins may have multiple putative predictions [31, 32, 33], whose location in the sequence may or may not overlap. This selection criterion may potentially lead to missing annotations and is therefore not suitable in complex PFA scenarios. To tackle this problem, domain-specific PFA is necessary. A simple approach, previously discussed in Yeats et al.[31], would be to order the predictions by their significance and iteratively add the most significant one, as long as it does not overlap with the already added predictions (henceforth referred to as the **heuristic** algorithm). Due to the biased selection of the first prediction, this algorithm does not guarantee an optimal solution (e.g., a protein sequence may have multiple similarly significant predictions). It has been previously shown that incorporating prediction significance and length may produce better results [34]. We implemented a Depth-First Search (**DFS**) algorithm that improves on the previous approaches.

The selection of reference HMMs is also critical, as PFA will ultimately be based on the available reference data. Whilst using unspecific HMMs to annotate a taxonomically classified sample may result in a fair amount of true-positives (correct annotations), depending on the confidence threshold used, it may also increase the rate of false-positives (over-annotation, due to a less strict confidence threshold) or false-negatives (under-annotation, due to a more strict confidence threshold) [35]. Using taxa-specific HMMs (TSHMM) rather than unspecific HMMs should, in principle, provide better annotations on a taxonomically classified sample, a feature that is already integrated into some PFA tools such as eggNOG-mapper [15] and RAST [16]. In essence, TSHMMs-based annotation limits the available search space, which may have positive and negative consequences. Since the search space is more specific, the annotations produced should be of higher quality; however, this higher specificity of the TSHMM could also lead to under-annotation (incomplete reference TSHMMs) or mis-annotations (low-quality reference TSHMM) [36]. This underlines the necessity to use specific (e.g., TSHMMs) and unspecific HMMs in a complementary manner. In this regard, the use of multiple sources of reference data remains a challenging aspect of PFA, and, with multiple high-quality reference data sources available, it is increasingly important to coalesce knowledge from different sources. While some PFA tools allow for the use of multiple reference data sources, either as a separate [25] or a unified [15, 37] database, it is still challenging to integrate multiple data sources dynamically.

When using reference data from multiple high-quality sources, the most common and straightforward approach is to consider the output from each reference data source independently (e.g., [25]). However, by doing so, we overlook that many sources can overlap and/or complement each other. Commonly this is compensated via manual curation, which is feasible only for a limited number of annotations. An automated approach would be to assume only the most significant annotation source for any given sequence and disregard other sources; this may result in vast losses of potentially valid and complementary information (e.g., database identifiers). As this is not desirable, the challenge is both in deciding which source(s) provide the best annotation as well as identifying complementary annotations. In the current context, complementary annotations can be defined as functional annotations that are functionally similar but originate from difference data sources; as such, while functionally similar, different data sources are likely to contain information that is absent in other data sources and vice versa. This unique functional information (i.e., database identifiers or functional descriptions) may prove essential in downstream data analysis. A straightforward approach to verify if functional annotations are functionally similar is to check whether they share a database identifier (ID), for example:

i. Function: "Responsible for glucose degradation"; IDs: K00844, EC:2.7.1.1, **PF03727**
ii. Function: "Responsible for glucose degradation"; IDs: P52789, **PF03727**, IPR022673

We can observe that the annotations (i) and (ii) share the database ID **PF03727**, thus it can be concluded that these annotations are functionally similar. If we were only to select the first annotation, we would ignore potentially useful information (IDs P52789 and IPR022673). However, it may be the case that no IDs are shared between the different annotations, for example:

i. Function: **"Responsible for glucose degradation"**; IDs: K00844, EC:2.7.1.1
ii. Function: **"Responsible for glucose degradation"**; IDs: P52789, IPR022673

We can observe that even though the annotations (i) and (ii) no longer share an ID, they still have the same function "**Responsible for glucose degradation**". Humans can quickly surmise that these annotations are the same as they share the same function description. Should the descriptions be identical or very similar, a machine could achieve the same conclusion with relative ease. However, in our experience, these free-text functional descriptions are often moderately or heavily dissim-

ilar [38, 39], with only a few keywords allowing us to ascertain they are indeed the same. This then makes it more difficult to use multiple reference data sources. For example:

i. Function: "Responsible for **glucose degradation**"; IDs: K00844, EC:2.7.1.1
ii. Function: "Protein is an enzyme and it is responsible for the **breakdown** of **glucose**"; IDs: HXK2_HUMAN

In such a scenario, someone trained in a biology-related field can quickly identify the most important words ("degradation"/"breakdown" and "glucose") in both sentences and conclude both annotations point to the same biological function. The challenge is now to enable a machine, deprived of any intellect and intuition, to eliminate confounders (ubiquitous words, e.g., "the"), identify keywords and their potential synonyms, and reach the same conclusion. A possible strategy is to use text mining, which is the process of exploring and analysing large amounts of unstructured text data aided by software, identifying potential concepts, patterns, topics, keywords, and other attributes in the data [40]. Text mining has been previously used with biological data [41, 42, 43, 44, 45], and even more specifically with regards to gene ontologies [46, 47, 48, 49, 50, 51] and PFA [43]. However, to our knowledge, there is no tool for the dynamic generation of a consensus from multiple protein annotations. This paper solves the problem of scaling the integration of different annotation sources, integrating a compact and flexible text mining strategy. We implemented a two-fold approach to build a consensus annotation, first by checking for any intersecting annotation IDs and second by evaluating how similar the free-text functional descriptions are. This approach attempts to address three very relevant issues with PFA [35, 36, 52, 53]: over-annotation, under-annotation, and redundancy. Another challenge in PFA is the lack of flexibility of some tools, as these are often intrinsically connected to their in-house generated reference data, and therefore hard to customize. In contrast, we developed a tool that, while offering high-quality unspecific and specific HMMs, is independent of its reference data, thus being customizable and allowing dynamic integration of new data sources.

We hereby present Mantis, a Python-based PFA tool that overcomes the previously presented issues, producing high-quality annotations with the integration of multiple domains and multiple reference data sources. Mantis automatically downloads and compiles several high-quality reference data sources and efficiently uses the available hardware through parallelized execution. Mantis is independent of any of the default reference data, resulting in a versatile and reproducible tool that overcomes the challenge of high-throughput protein annotation coming from the many genome and metagenome sequencing projects.

Mantis

Mantis is available at `https://github.com/PedroMTQ/mantis`, and its workflow (see **Figure 1**) consists of six main steps: (i) sample pre-processing, (ii) HMM profile-based homology search, (iii) intra-HMM hits processing, (iv) metadata integration, (v) inter-HMMs hits processing, and (vi) consensus generation. For future reference, an instance when an HMM matches with a protein sequence is referred to as a "hit". The workflow starts with sample pre-processing, in which the sample(s) is/are split into chunks. This is followed by homology search, where query sequences are searched against the available reference data using HMMER. During intra-HMM hits processing the DFS algorithm is used to generate and select the best combination of

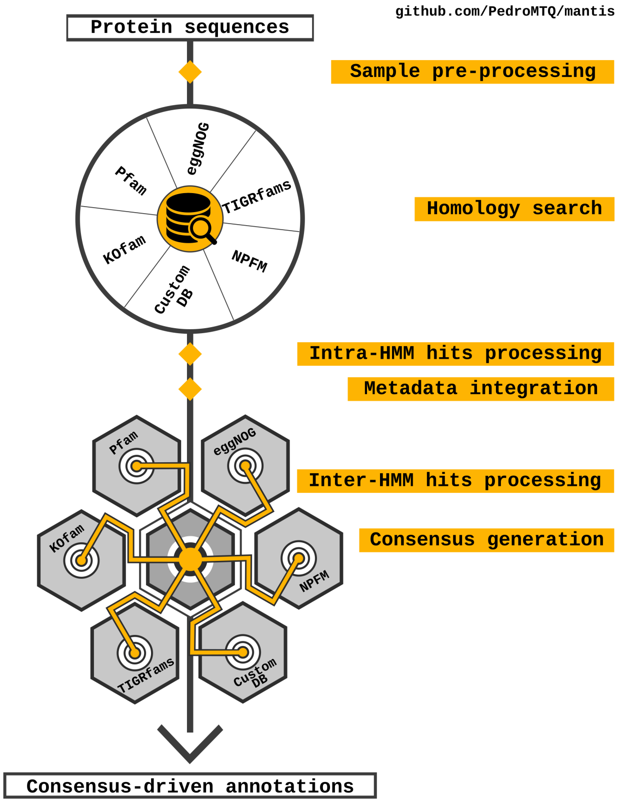

Figure 1. Overview of the Mantis workflow. KOfam [54], Pfam [55], eggNOG [56], NCBI protein family models (NPFM) [57], and TIGRfams [58] are the reference HMMs currently used in Mantis. CustomDB can be any HMM library provided by the user.

hits per HMM source; **Figure 2** shows how different algorithms may lead to a different selection of hits. Metadata integration adds the metadata (functional description and IDs) to the respective hits. During inter-HMMs hits processing, the DFS algorithm is used to generate all the combinations of hits from all HMM sources (in this step all hits are pooled together). Finally, consensus generation ensures the best combination of hits among all hits from the multiple reference data sources is selected. This combination is expanded by adding additional hits with consistent metadata (intersecting identifiers or similar functional descriptions). Please refer to the **Methods** section for a detailed description of all these steps. We provide default execution parameters, however, the user is free to fully customize Mantis, not only the parameters but also the reference databases used. Mantis requires a FASTA formatted protein sequence file as input, where the user can also provide the organism's taxon which will allow for taxa-specific annotation. Reference databases are downloaded automatically. The MANTIS.config file allows for configuration of the reference data, its respective weights and enables the compilation of specific eggNOG TSHMMs. For more details, see the documentation at [59]. Due to issues with Python's multiprocessing in MacOS, and the fact that HMMER is not available on Windows, Mantis is only available on Linux-based systems.

Analysis

To analyse and validate the performance of Mantis, we performed several in-silico experiments. We annotated a reference dataset containing curated protein entries from UniProt to set default parameters and evaluate the impact of different Mantis' features: (i) impact of the e-value threshold; (ii) im-

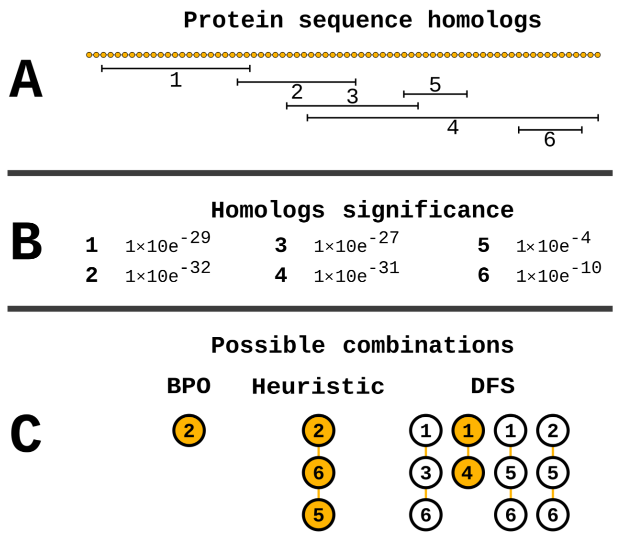

Figure 2. Homolog selection for the three hit processing algorithms in Mantis. The selection of the hit(s) depends on the underlying algorithm. In the case of the portrayed protein with six hits (A) (which are overlapping to various degree) that have varying significance values (B) the three algorithms would behave as follows: (i) BPO would select only the most significant hit (#2); (ii) the heuristic algorithm initially selects the most significant hit (#2) which then restricts (due to overlapping residues) the hits available for selection (hits #1, #3, and #4 can no longer be selected), leading to the selection of the next most significant hit (#6), and finally the selection of hit #5; (iii) the DFS algorithm generates all possible combinations of hits, which are then scored according to the e-value, hit coverage and total combination coverage (for more details, please see "**Multiple hits per protein**". According to these parameters, the most likely combinations of hits would be hits #1 and #4.

pact of the hit processing algorithm; (iii) how each reference data source contributed to the final output and (iv) impact of the consensus generation on annotation quality. Furthermore, we annotated several sequenced organisms, with and without TSHMMs, thus evaluating the impact of using taxa-resolved reference data. Finally, we compared Mantis against eggNOG-mapper [15] and Prokka [14]. A description of the samples used for this benchmark is available in "**Sample selection**". Prokka was only used for the annotation of prokaryotic data (i.e., all except for *Saccharomyces cerevisiae* and *Cryptococcus neoformans*). In order to compare the performance between the different tests, we calculated a confusion matrix for each test. For future reference, a **True-Positive** (TP) occurs when a functional annotation (predicted from a PFA tool) shares one or more database IDs with the respective reference annotation (e.g., Pfam ID); a **False-Positive** (FP) when no database IDs are shared; a **False-Negative** (FN) when the PFA tool does not annotate a protein sequence but a reference annotation is available; and a **True-Negative** (TN) when the PFA tool does not annotate a protein sequence and no reference annotation is available. **Precision** is defined as $\frac{TP}{TP+FP}$, **Recall** as $\frac{TP}{TP+FN}$, and **F1 score**(harmonic mean of precision and recall) as $2 \times \frac{Precision \times Recall}{Precision+Recall}$. The F1 score is used as a performance metric. Further details on the benchmark are available in "**Establishing a test environment**".

Initial quality control

Function assignment e-value threshold

It is known that the e-value threshold directly affects annotation quality, however, no gold-standard threshold exists [34]. Depending on the reference data source's size, quality, and specificity, we may use more or less stringent thresholds. It is therefore essential to test annotation quality with different thresholds. As such, we tested different static e-value thresholds and a dynamic threshold, which has been described in "**Testing different e-value thresholds**". As can be seen in the supplemental **Table 1**, precision was similar across the range of e-value thresholds tested, with recall/sensitivity decreasing with lower e-value thresholds. Unexpectedly, unlike recall, precision was not directly correlated with the e-value threshold; indeed a maximum precision of 0.747 was obtained for the e-value threshold $1e^{-6}$, with precision slightly decreasing with more stringent e-value thresholds. A maximum F1 score of 0.827 was observed for the e-value threshold $1e^{-3}$, as such, we chose this value as the default e-value threshold for Mantis.

Impact of hit processing algorithms

To understand whether the different hit processing algorithms resulted in statistically significant differences in F1 scores, we created synthetic samples and performed pairwise comparisons between the DFS and the other algorithms: (i) DFS and heuristic, and (ii) DFS and BPO. We rejected the H_0: "no differences in F1 score between the tested algorithms" in both comparisons since p-value < 0.01. The DFS algorithm resulted in a greater F1 score (mean = 0.827) than the heuristic (mean = 0.826) and BPO (mean = 0.816) algorithms. Further details on results can be found in the supplemental **Table 2**, and further details on the testing method can be found in "**Testing hit processing algorithms**".

Impact of sample selection

Testing exclusively against well-annotated organisms is a recurring issue with protein annotation benchmarking, resulting in the re-annotation of sequences already present in the reference data used, leading to a biased annotation quality evaluation. To avoid this bias, we downloaded all the curated UniProt (i.e., Swiss-Prot) protein entries (as of 2020/04/14) and selected entries by their creation date such that we have four samples that contain protein entries created in different date ranges (2010-2020, 2015-2020, 2018-2020, and 2020). Samples with more recent protein entries are increasingly more likely to lack any proteins used to generate Mantis' reference data, which increases the likelihood that potential annotations are due to true sequence homology (and not to circular re-annotations). We annotated these samples using three different hit processing algorithms (DFS, heuristic, and BPO), determining the impact of each on the F1 score.

As seen in **Figure 3**, the F1 score decreased as the sample was restricted to more recent data. As seen in the supplemental **Table 3**, when comparing the hit processing algorithms, we found that the DFS algorithm consistently outperformed the other algorithms, with an average F1 score 0.021 and 0.003 higher than the BPO and heuristic algorithms, respectively. In addition, the F1 score difference between the multiple hits algorithms (DFS and heuristic) and the single hit algorithm (BPO) increased as the entries in a sample were restricted to more recent years.

Contribution of the different reference data sources

We analysed each reference data source's contribution to the output annotation for the UniProt 2010-2020 sample. By checking the column "*HMM_files*" in the *consensus_annotation.tsv* file, we found that Pfam was present in 24.4% of the sequence annotations, KOfam in 62.37%, eggNOG in 76.52%, NPFM in 13.91%, and TIGRfam in 12.96%. Note that, since multiple reference data sources may be present in one sequence (due to the consensus generation and hit processing algorithms), the sum of the previous values is above 100%.

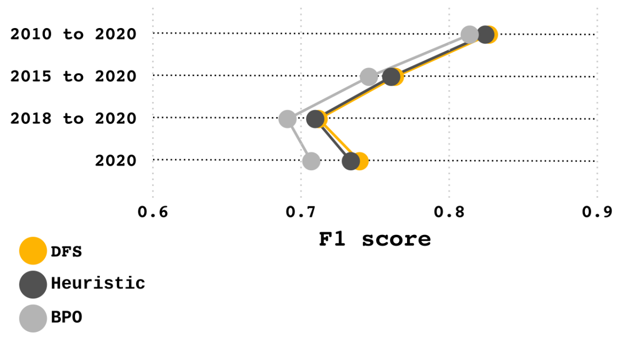

Figure 3. Annotation F1 score per hit processing algorithm and sample. Overall, the DFS and heuristic algorithms achieve similar results, outperforming the BPO algorithm.

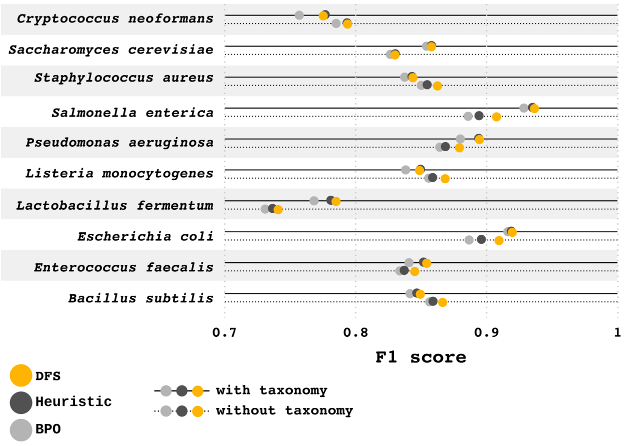

Figure 4. F1 score per hit processing algorithm and organism, with and without using taxonomy information. F1 score was higher for well-studied organisms, TSHMMs also tend to perform better with these organisms.

Impact of consensus generation

During consensus generation, two methods are used for checking the consistency of the hits metadata: IDs intersection and text mining. We analysed the contribution of both methods for the annotation of the UniProt 2010-2020 sample, and found that roughly 35.1% of the consistency checks were due to the text mining approach, and the remaining were due to IDs intersection.

We also tested the impact of text mining on annotation performance: to do so, we annotated the Uniprot 2010-2020 sample but restricted the consensus generation in different manners and with different algorithms. Six different test conditions were created: (i) DFS with default consensus generation, (ii) DFS with consensus generation restricted to IDs (i.e., IDs intersection but no text mining), (iii) DFS without consensus generation (i.e., neither IDs intersection nor text mining), (iv) BPO with default consensus generation, (v) BPO with consensus generation restricted to IDs, and (vi) BPO without consensus generation. We also annotated the same sample using eggNOG-mapper - condition (vii). Prokka was not used here since the current sample contains non-prokaryotic data. The F1 scores were as follows: (i) 0.827, (ii) 0.790, (iii) 0.774, (iv) 0.814, (v) 0.779, and (vi) 0.763, and (vii) 0.703. Further details can be found in supplemental **Table 4**.

Hit processing approximation

During hit processing, two algorithms may be used, the DFS, and, as a backup (if the DFS algorithm's runtime exceeds 60 seconds), the heuristic. We calculated how many times the heuristic algorithm was used as a backup during the hit processing of the 2010-2020 UniProt sample. We found that for the intra-HMM hit processing, the heuristic algorithm was used in roughly 7.2% of the sequences, and for the inter-HMMs hit processing in 0.5% of the sequences.

Quality control with sequenced organisms

As a secondary quality control, to assess the impact on F1 score when using taxa-resolved reference data, we annotated several sequenced organisms (for more details, see the supplemental **Table 5**) with and without TSHMMs. We also evaluated the impact of the different hit processing algorithms on these samples. As seen in **Figure 4**, well-studied organisms (e.g., *Saccharomyces cerevisiae*) had better annotations, especially when applying TSHMMs, unlike poorly described organisms. The average F1 score gain with TSHMMs was 0.006. With TSHMMs, the DFS algorithm had, on average, 0.001 and 0.010 higher F1 scores than the heuristic and BPO algorithms, respectively. Without TSHMMs, the DFS algorithm had, on average, 0.008 and 0.013 higher F1 scores than the heuristic and BPO algorithms, respectively. Further details can be found in the supplemental **Table 6**.

Comparison between Mantis and other PFA tools

The sequenced organisms enumerated in the supplemental **Table 5** were annotated with Mantis, eggNOG-mapper, and Prokka (for the latter non-prokaryote organisms were excluded). To evaluate the added value of using the very comprehensive eggNOG reference data source, we also assessed Mantis' F1 score using different reference data. In total, six different tests were performed for each organism: (i) Mantis with default data sources and with taxonomy information; (ii) Mantis with default data sources except for eggNOG's data and with taxonomy information; (iii) Mantis with default data sources but without taxonomy information; (iv) eggNOG-mapper without tax scope option; (v) eggNOG-mapper with tax scope option; (vi) Prokka with default data sources and default execution.

On average, (i) had a F1 score and annotation coverage of 0.857 and 96.56%, respectively, (ii) 0.832 and 89.82%, (iii) 0.850 and 96.14%, (iv) 0.734 and 88.45%, (v) 0.725 and 88.02%, and (vi) 0.507 and 62.38%. As seen in **Figure 5**, Mantis outperformed the other PFA tools in all tests (with one exception in the organism *Saccharomyces cerevisiae*, where eggNOG-mapper without taxonomy had an F1 score of 0.841 and Mantis without taxonomy had an F1 score of 0.830). The average Mantis F1 score with default execution and TSHMMs was 0.131 higher than eggNOG-mapper (with tax scope) and 0.360 higher than Prokka. Mantis' setting without the eggNOG reference data had an average F1 score 0.107 higher than eggNOG-mapper (both tools with taxonomy information) and an average F1 score 0.025 lower than Mantis' with the eggNOG reference data. Further details are available in the supplemental **Table 7**.

Annotating metagenomes

To our knowledge, there are no manually curated metagenome annotations, therefore annotation validation was not performed, instead we only calculated the annotation coverage. We selected four samples from different environments and predicted the protein coding genes with Prodigal v2.6.3 [60]. The annotated samples were:

- Biogas highly efficient cellulose-degrading consortium

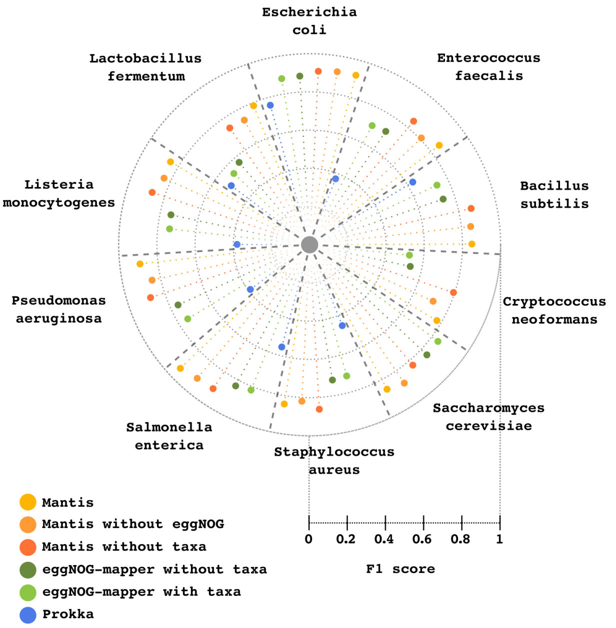

Figure 5. Annotation F1 score of Mantis, eggNOG-mapper, and Prokka using different reference data. Each slice represents an organism and contains the F1 score obtained between the different conditions.

(SEM1b) [61, 62] with 39411 sequences;
- Glacier-fed stream sediment (GFS) [63] with 270341 sequences (phenol-chloroform extraction batch number 37);
- Marine [64] with 605043 sequences (ERR1726751);
- Human gut microbiome (MuSt [7]) with 692061 sequences (M05-01-V1).

The performance of Mantis varied per metagenome sample; it annotated 213539, 162133, 33016, and 559792 sequences in the samples GFS, marine, SEM1b, and MuSt, respectively. The respective annotation coverage was as follows: 78.99%, 26.80%, 83.77%, and 80.89%. We repeated the same test for eggNOG-mapper and Prokka (in the case of Prokka by annotating the original nucleotide sequences), the coverage for the samples GFS, marine, SEM1b, and MuSt, was, respectively, 77.52% and 10.87%, 16.21% and 1.01%, 81.95% and 32.32%, and 78.72% and 20.37%.

Computational efficiency

We ran Mantis against samples with a different number of sequences and a different number of available CPUs. We performed this test for the DFS and heuristic algorithm only. As expected, we found that the heuristic algorithm was faster than the DFS algorithm. The heuristic algorithm was, on average, 1.42 times faster than the DFS algorithm. As expected, runtimes were inversely correlated to the number of CPUs and sequences. Further details can be found in the supplemental **Table 8**.

We also aimed at allowing Mantis to be run on personal computers, which requires removing the eggNOG dataset. However, as we have previously shown in **Comparison between Mantis and other PFA tools**, this does not cause a high impact on F1 score. We annotated the previously enumerated sequenced organisms (supplemental **Table 5**) on a Dell XPS 13-9370 with Ubuntu 20.04.1 LTS 64 bit, 16GB RAM, 512 GB SSD, and an 8 core Intel Core i7-8550U CPU. The average runtime for prokaryotes and eukaryotes was 28 and 93 minutes, respectively. Further details are available in the supplemental **Table 9**.

Discussion

We herein presented Mantis, an open-access PFA tool that produces high-quality annotations and is easily installed and integrated into other bioinformatic workflows. Mantis uses a well-established homology-based method and produces high-quality consensus-driven annotations by relying on the synergy between multiple reference data sources and improved hit processing algorithms.

Mantis addresses some major challenges in PFA, such as flexibility, speed, the integration of multiple reference data sources, and use of domain-specific annotations. It also addresses under-annotation through the use of multiple reference data sources, which implicitly leads to a wider search space. Additionally, redundancy, which is a drawback inherent to consensus-driven annotation, is ameliorated by removing duplicate database IDs and/or identical descriptions. We have attempted to avoid over-annotation through the generation of a consensus-driven annotation, which identifies and merges annotations that are consistent (i.e., similar function) with each other (e.g., if three out of five independent sources point towards the same function and two others point towards other, unrelated functions, then these three annotations are more likely to be valid), and eliminating the remaining inconsistent annotations.

We have shown that a stricter/lower e-value threshold did not necessarily lead to a higher F1 score. As expected, a lower threshold restricted the amount of hits, lowering the recall. However, we also found that more stringent e-value thresholds may result in a lower precision; this behaviour is connected to Mantis's consensus generation and hit combinations scoring. A thorough explanation is available in the supplemental PDF.

Well-curated and commonly used resources were chosen as the default reference data sources for Mantis, containing both unspecific and specific reference data (e.g., taxa-specific). As we have shown, no single reference data source accounted for most annotations, each offering both unique and overlapping insight into protein function, thus confirming their synergy and partial redundancy. These are integrated through a consensus-driven approach, which Mantis uses as an additional quality control step, and a means to automatically incorporate a broader variety of IDs. The intersection of IDs was, as expected, the main contributor towards this integration (since most databases provide cross-linking), however, we found that the text mining approach still contributed considerably (35.12% for the UniProt 2010-2020 sample), which clearly highlights the need to use such a method.

We additionally evaluated the impact of not using text mining during consensus generation and removing the consensus generation altogether on the DFS and BPO algorithms. The benchmark using the BPO algorithm without consensus generation represented the baseline approach towards the integration of multiple reference data sources (merely selecting the most significant hit during inter and intra-HMMs hit processing). In contrast, the benchmark using the DFS algorithm with the consensus generation depicted the accumulation of all the features introduced by Mantis. Overall, we found a difference of 0.064 in F1 scores, which suggests the additive effect of Mantis's various data integration methods. Mantis, in respect to this specific benchmark, also obtained a F1 score higher than eggNOG-mapper in all conditions, which suggests the importance of using multiple reference data sources.

We have implemented two algorithms for domain-specific homologs search (DFS and heuristic as backup), and have not only shown that these algorithms perform better when annotating previously described protein sequences, but that their impact on the F1 score increased when annotating previously uncharacterized protein sequences (e.g., average F1 score gain

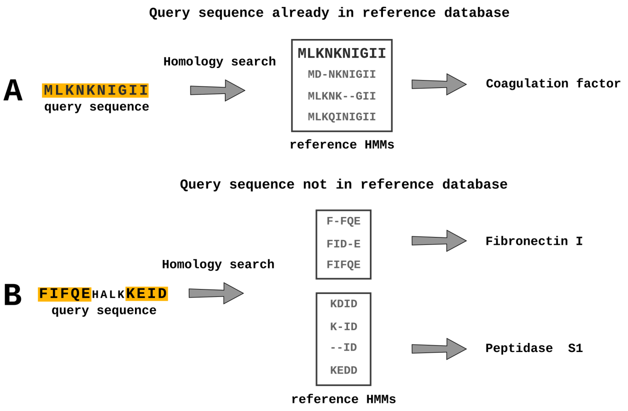

Figure 6. The impact of the reference data completeness on protein function annotation. A. the functional prediction is facilitated by the query sequence being previously identified and included in the reference HMMs. **B.** if the query sequence has not been previously annotated, multiple regions in the protein may match with different reference HMMs.

with DFS and BPO algorithms in the UniProt 2010-2020 and 2020 samples was 0.013 and 0.033, respectively). We hypothesize that for the latter, a homology search is not capable of finding whole-sequence homologs, finding, however, multiple domains that partially constitute the protein sequence. As such, we argue that by increasing the resolution (sequence homology to domain homology) of homology-based reference data, domain-specific algorithms may become increasingly valuable. We think this would be especially important when annotating protein sequences without well-described homologs but that contain previously characterized conserved protein domains. In **Figure 6.A**, we can observe that the current query sequence is already used to generate the HMM profiles in the reference data, matching with the HMM profile containing it. Such a scenario is common when annotating well-described organisms (e.g., *Escherichia coli*). However, as is often the case when annotating non-model organisms and metagenomes, the query sequence is absent from the reference data (**Figure 6.B**), thus partially matching with several HMMs (which may correspond to multiple domains, depending on the resolution of the reference data). Unlike the BPO algorithm, the heuristic and DFS algorithms are able to incorporate multiple homologs. While these may not be enough to determine a protein's biological function, they still provide a better biological context than a single functional annotation.

Further improvements in annotation quality may also require the use of motif-based and/or genomic context-based (e.g., operon context information, co-expression, and subsystems) methods such as those described by Sigrist et al.[65], Mooney et al. [66], Mavromatis et al.[67], Overbeek et al.[21], and Hannigan et al.[68]. Nevertheless, the significantly higher F1 score seen when comparing the DFS and BPO algorithms highlights the need to adopt better hit processing methods, especially for non-model organisms. With samples ranging from thousands to millions of protein sequences, sub-optimal hit processing algorithms may cascade into unnoticeable pitfalls in downstream data analysis (e.g., accumulation of incomplete or low-quality genome annotation, which may lead to false biological interpretations). While we have shown that the DFS algorithm outperforms the heuristic algorithm, both achieve a very similar F1 score when applied to non-synthetic samples; since the heuristic algorithm is much more time efficient (as seen in supplemental **Table 8**), a user may confidently set it as primary algorithm.

The use of TSHMMs resulted in a 0.006 higher F1 score, however, this improvement (as seen in **Figure 4**) was not consistent across all the annotated organisms (as expected, a similar trend was also seen with eggNOG-mapper). We believe this is due to a poorer quality of the TSHMMs for some organisms, which is a consequence of the issues with the current taxonomy classification system [69, 70] and lack of knowledge regarding highly resolved taxa [71]. Model organisms such as *Escherichia coli* and *Saccharomyces cerevisiae* clearly benefited from TSHMMs, both since the reference data already contains data specific to these organisms and that functions of proteins within model organisms are better experimentally described. Conversely, non-model organisms are often only computationally annotated by association, contributing to a weaker reference annotation (which can be observed by the higher rate of potentially new annotations in these organisms, as seen in the supplemental **Table 6**). Nonetheless, while experimental evidence remains the gold standard, it is unfeasible to ignore the need for computational methods to infer function. While steps in this direction have been taken [56, 16], taxa-resolved PFA remains a challenge.

We benchmarked Mantis against two other PFA tools - eggNOG-mapper and Prokka, and have shown that Mantis achieves a higher F1 score (0.131 higher than eggNOG-mapper and 0.350 higher than Prokka). Although Mantis' default execution heavily relies on the eggNOG reference data, we have also shown that even without it, it is possible to achieve an almost similar F1 score. This attests to the quality of the various reference data used, showcasing as well the possibility of running Mantis on a personal computer (something that would be impossible with eggNOG's prohibitive size).

We also evaluated the annotation coverage of Mantis and the other PFA tools when annotating metagenomes. Mantis had the highest annotation coverage among the tested PFA tools, but eggNOG-mapper was close behind. All PFA tools had a low annotation coverage for the marine sample. We believe this may be due to a lack of reference HMMs for this specific environment. This metagenomic sample has data from varying ocean depths, with many novel sequences from viruses, prokaryotes, and picoeukaryotes [72].

Finally, as shown in **Accessibility and Scaling**, a conda environment and automated reference data download are provided. In addition, Mantis accepts several formats as input (i.e., protein FASTA file, TSV file with paths, directories, or compressed archives), outputting easy to parse TSV files. We believe these features address some of the reproducibility challenges the bioinformatics community still faces [73].

As discussed, there is still room for improvement in the hit processing algorithm DFS (since it does not provide large F1 score gains over the heuristic algorithm). In the future, Mantis could also include genomic context-based annotation methods. Despite the previously discussed challenges, we have clearly shown that Mantis is a flexible tool while also producing annotations with high precision and recall.

Conclusion

By making use of the synergistic nature of differently sourced high-quality reference data, Mantis produces reliable homology-based annotations. By allowing for total customization of these reference data, Mantis is also flexible, easily integrated and adapted towards various research goals. In conclusion, we have shown that Mantis addresses a number of the current PFA challenges, resulting in a highly competitive PFA tool.

Methods

Accessibility and Scaling

Mantis automatically sets up its reference data by downloading HMMs from different sources, and, when necessary, reformatting the data to a standardized format and downloading any relevant metadata. Reference data can be customized via a config file. It also dynamically configures its execution depending on the resources available. A conda environment and extensive documentation [59] are available.

Mantis splits most of the workflow into sub-tasks and subsequently parallelizes them by continuously releasing tasks to workers from a global queue (via Python's multiprocessing module). During each main task of the annotation workflow, workers are recruited (the number of workers depends on the available hardware and work required), these will then execute all the queue tasks. When a worker has finished its job, it will execute another task from the queue, until there are no more tasks to execute. If the queue is well balanced, minimal idle time (time spent waiting for workers to get a new task) can be achieved. Load balancing is achieved by splitting the sample and reference data into chunks. During setup, large reference data sources (more than 5000 HMM profiles) are split into smaller chunks, this enables parallelization and ensures each annotation sub-task takes approximately the same time. Samples are equally split into chunks (sample chunk size is dynamically calculated). If the sample has 200,000 or fewer sequences, sequences are distributed by their length among the different chunks, so that each chunk has approximately the same number of residues. If the sample has more than 200,000 sequences, then sequences are distributed to each chunk independently of their length (this alternative method is an efficiency safeguard). This two-fold splitting achieves quasi-optimal load balancing. With the sample and reference data in chunks, posterior workflow steps can be parallelized wherever applicable. To note that Mantis uses HMMER's hmmsearch for homology search, which outputs an e-value scaled to the sample/chunk size. Since Mantis splits the samples into chunks, during hit processing, the e-value is scaled to the original sample size.

Input and output

MANTIS accepts protein sequence FASTA files as input. If the sample has been previously taxonomically classified, the user can add this information when running Mantis. For example, if annotating an *Escherichia coli* sample, the user could add *–od* followed by the NCBI ID or the organism name:

```
$ python mantis run_mantis -t sample.faa -od 562
```

Mantis outputs, for each sample, three tab-separated files, each corresponding to a different step in Mantis' workflow: (i) a raw output *output_annotation.tsv* (generated during **Figure 1.Intra-HMM hits processing**), with all the hits, their e-value, and coordinates; (ii) *integrated_annotation.tsv* (generated during **Figure 1.Metadata integration**), with the same information as *output_annotation.tsv*, but also with hits metadata (e.g., KEGG orthology IDs (KO), enzyme commission (EC) numbers, free-text functional description, etc); and (iii) the main output file *consensus_annotation.tsv* (generated during **Figure 1.Consenus generation**), with each query protein ID and their respective consensus annotation from the different reference data sources (e.g., Pfam). These files provide contextualized output in a format that is both human and machine-readable. A *Mantis.out* file is also provided per sample, serving as a log file for each execution step.

Reference data and customization

Mantis, by default, uses multiple high-quality reference HMM sources – Pfam [55], eggNOG [56], NPFM [57], KOfam [54], and TIGRfam [58] (these default HMMs can be partially or entirely removed). To find more meaningful homologs through taxa-specific annotation, Mantis uses TSHMMs, originally compiled by eggNOG and NPFM. eggNOG TSHMMs were compiled by downloading all the TSHMMs at `http://eggnog5.embl.de/download/latest/per_tax_level/`, their respective metadata originates from the metadata available in the previous link as well as the metadata within the eggNOG-mapper SQL database. NPFM TSHMMs were compiled by downloading all the NPFM HMMs at `https://ftp.ncbi.nlm.nih.gov/hmm/current/` and assigning each HMM into their respective TSHMM. A general NPFM HMM was created by pooling all non-assigned HMM profiles and the TSHMMS from the following NCBI IDs: 2157 (*Archaea*), 2 (*Bacteria*), 2759 (*Eukaryota*), 10239 (*Viruses*), 28384 (Others), and 12908 (Unclassified). These IDs correspond to NCBI's top level taxonomy rank IDs. A general eggNOG HMM was created by pooling together the TSHMMs from the same aforementioned NCBI taxon IDs. The user can customize which eggNOG TSHMMs are downloaded by Mantis by adding the line *nog_tax = NCBI_ID*1, *NCBI_ID*2 to the config file. Custom HMM sources can also be added by the user, metadata integration of these is also possible (an example is available in Mantis' repository). Since some sources are more specific than others, the user may also customize the weight given to each source during consensus generation. HMM profiles often only possess an ID respective to the database they were downloaded from, which may not directly provide any discernible information. Mantis, when necessary, ensures that the hits from these HMMs are linked to their respective metadata. For future reference, while an HMM is an individual profile, Mantis compiles all related HMM profiles into a single file making it indexable by HMMER. Thus when a certain HMM source is mentioned, it refers to the collection of related HMM profiles.

Taxa-specific annotation

Taxa-specific annotation (TSA) uses the TSHMMs and unspecific HMM made available by eggNOG and NPFM. TSA, however, works differently from the annotation method of the other reference data. When given taxonomy information (either a taxa name or NCBI ID) the organism's taxonomic lineage is computed (e.g., for *Escherichia coli* the lineage would be `2 - 1224 - 1236 - 91347 - 543 - 561 - 562`). TSA starts by searching for homologs in the most resolved TSHMM (in this case for taxa 562, if it exists). All valid homologs (respecting the e-value threshold) are extracted for each query sequence, and unannotated sequences are compiled into an intermediate FASTA file. A new homology search round starts with the sequences in the current intermediate FASTA, but now in the TSHMM one level above (in this case the TSHMM 561). This cycle repeats until all query sequences have valid homologs or until there are no more TSHMMs to search for. If there are still sequences to annotate, then these homologs are searched for in the general eggNOG and NPFM HMMs. If no taxonomy information is given, the homology search starts with the general NPFM and eggNOG HMMs. Non-taxa specific HMMs (i.e., Pfam, KOfam, and TIGRfams) are always used, regardless of the sample's taxonomy.

Multiple hits per protein

HMMER outputs a *domtblout* file [24], where each line corresponds to a hit/match between the reference data and the query

protein sequence. The e-value threshold within the HMMER command limits the amount of hits to be analyzed in the posterior processing steps. Each hit, among other information, contains the coordinates where the query sequences matched with the reference HMM profiles and the respective confidence score (e-value) (**Figure 2.A and .B**). Mantis uses HMMER's independent e-value when using the DFS and heuristic algorithms, whereas it uses the full sequence e-value when using the BPO algorithm (since only the best hit is extracted per protein sequence). For simplicity purposes, both are simply referred to as e-value throughout this paper. The annotation of a protein sequence with multiple hits is a nontrivial problem, thus requiring the implementation of a method for the processing of hits. We designed a method that generates and evaluates all possible combinations of hits by applying the DFS algorithm [74]. This algorithm allows the traversal of a tree-structured search space (i.e., each node is a hit), whilst pruning solutions that do not respect predefined constraints (i.e., overlapping hit residues coordinates), backtracking from leaf to root until the possible solution space is exhausted. Our method generates all the possible combination hits with the following method: (i) Get one hit from the collection of hits and define it as the combination root hit; (ii) Check which other hits overlap up to 10% (default value) [31] with previous hits and select one to add to our current combination of hits; (iii) Repeat step (ii) until no more hits can be added; (iv) Repeat steps (i-iii) so that we loop over all the other hits and all possible combinations are generated. We used Cython [75] to speed up the DFS implementation. Cython is an optimising static compiler for the Python programming language, allowing the compiler to generate C code from Cython code, in this case, functioning as a wrapper for the DFS algorithm. The total number of possible combinations is $2^N - X - 1$, where N is the number of hits the protein sequence has, X the number of impossible combinations (combinations with overlapping hits), and *1* the empty combination. Due to exponential scaling, this method is not always computationally feasible (e.g., the query sequence is very large and has many small-sized hits). In such a scenario, the DFS algorithm may exceed the system's recursion limit or be unable to find a solution in optimal time (60 seconds by default, but customizable). Should this happen, Mantis employs the previously described heuristic algorithm, which scales linearly (a warning is written in the *Mantis.out* log).

After generating all the possible combinations, each combination is evaluated according to several parameters:

- $query_{length}$ - number of residues in the query sequence.
- hit_{length} - number of residues in the hit.
- $combo_{length}$ - number of hits in the respective combination.
- *Total coverage* (**TC**) - number of non-redundant residues in all the combination's hits divided by $query_{length}$. A high TC implies the combination covers a large percentage of the protein sequence.
- *Average hit coverage* (**HC**) - sum of the coverage of each hit ($\frac{hit_{length}}{query_{length}}$). This sum is then averaged by dividing by $combo_{length}$. A high HC implies the hits in the combination are large, thus benefiting combinations with a low amount of large hits rather than combinations with a high amount of small hits.
- *Combination e-value* (**CE**) - the e-value of each hit is scaled twice, once to reduce the range between different e-values (log10) and the second to understand how each hit e-value compares to the best/lowest hit e-value found for a particular sequence (minmax scaling). The scaled e-values are then summed and divided by $combo_{length}$.

The ***combination score*** is defined by the following equation:

$$TC \times HC \times CE \quad (1)$$

The combination with the highest ***combination score*** is then selected, where the available choices will ultimately depend on the algorithm used (**Figure 2.C**). Our intra-HMMs hit processing implementation thus applies a two-fold quality control, initially by limiting the amount of hits in HMMER's *domtblout* (i.e., e-value threshold) and secondly by hierarchically ordering and selecting the most significant combination of hits.

Using multiple reference data sources

An unannotated protein sequence may match with zero, one, or multiple reference HMM profiles, from one or more data sources. When a protein sequence has multiple hits from different data sources, it is important to identify functionally similar annotations so that no information is lost (i.e., functional descriptions or IDs that may be in one reference data source but not in another). By linking the metadata respective to the HMM profiles to the now annotated protein sequence, we can identify functionally similar annotations and integrate multiple reference data sources into one final consensus annotation. In this manner, functionally similar annotations are merged, and any complementary information they provide can then be used in downstream analysis (e.g., annotation 1 has a Pfam and KO ID, annotation 2 has an EC number and the same KO ID, merging these will result in a final annotation with more information).

For the integration of functional annotations from multiple data sources, a two-fold approach was used: (i) *Consensus between IDs*; and (ii) *Consensus between the free-text functional description*. The latter is used as a backup, since IDs cross-linking is not universally available. Each reference data source includes metadata relevant to the HMM profiles herein; this metadata may include multiple intra and/or inter database IDs as well as free-text functional descriptions. IDs are extracted either through source-specific metadata parsing and regular expressions. Free-text functional descriptions are extracted by source-specific metadata parsing. With this information it is then possible to identify annotations that are functionally similar/consistent, and may thus be complementary to each other. The *consensus between IDs* is calculated by identifying intersections between the functional annotations of different reference data sources (e.g., both annotations have the same Pfam ID). IDs within the free-text functional descriptions are extracted (with regular expressions) and also used here. If no consensus between IDs is found, then we proceed with a consensus calculation between functional descriptions (further described in the supplemental PDF).

Inter-HMMs hit processing starts by pooling together all hits from the different reference data sources and generating all possible combinations of hits (**Figure 7.A**). The same method used in intra-HMM hit processing is applied, where the DFS algorithm is used by default (again using the heuristic algorithm as a backup), but the BPO and heuristic algorithms can also be used. We then check the metadata consistency (either through IDs or free-text functional descriptions) of each hit against the current sequence's other hits. With this information, a metadata consistency graph is generated (**Figure 7.B**). With the metadata consistency graph and all possible combinations of hits, we can then calculate the ***consensus combination score*** using equation 2. This requires calculating of the ***combination score***, using equation 1. This score is then multiplied by an additional score, comprised of the following parameters:

- *Average hit consistency* (**HCN**) - number of hits (among all hits) with metadata directly consistent (i.e., nodes directly connected in the metadata consistency graph) to the hits in the current combination. Consistency checks are restricted to other reference data sources besides the hit own's reference source (e.g., if a hit is from Pfam, we would only check hits that are not from Pfam). This number, plus the number of hits in the combination, is divided by the total number of hits for the respective query sequence (e.g., if a combination has two hits, with these having metadata consistent with three other hits, and if there are ten hits in total, **HCN** would equal to $\frac{2+3}{10} = 0.5$). This is an important parameter since it entails independent sources are describing the same function.
- *Reference HMM weight* (**HMMW**) - average weight of all the reference data sources within the combination. This is calculated by adding all hits' HMM weights and dividing this sum by the number of hits in the combination (e.g., if a hit comes from Pfam, that has a weight of 1, and another from eggNOG, that has a weight of 0.8, **HMMW** would equal to $\frac{1+0.8}{2} = 0.9$). The default weight for each default reference data source has been set according to the authors' perception of the reference quality - creation method, curation level, and annotation completeness (eggNOG - 0.8, Pfam - 0.9, NPFM and KOfam - 0.7, and TIGRfam - 0.5). This weight is customizable, the default weight for custom reference data is 0.7 (which can also be customized).
- *Metadata quality* (**MQ**) - average metadata quality of each hit in the combination. If a hit has no annotation data (IDs or description) it is given a score of 0.25, 0.5 if only the description, 0.75 if only the IDs, 1 if IDs and description. All hit's metadata quality score is summed and divided by the number of hits in the combination.

Note that hit metadata consistency (through IDs or descriptions) requires a minimum of 70% residues overlap (default but can be changed). Using the previously calculated ***combination score***, we then calculate the ***consensus combination score*** using the following equation:

$$Combination_{score} \times \frac{HCN + HMMW + MQ}{3} \qquad (2)$$

The combination with the highest ***consensus combination score*** is selected and expanded by concatenating additional metadata from other consistent hits (**Figure 7.C**). In this step, consistent hits can be either directly or indirectly connected in the metadata consistency graph (a minimum of 70% residues overlap is still required). This expanded combination is then merged into the final query sequence consensus annotation (**Figure 7.D**). Redundant (i.e., repeated identifiers or functional descriptions) or poor quality information (e.g., "hypothetical protein") is removed from the consensus annotation.

Sample selection

As an initial testing dataset we started by downloading all the curated Uni-Prot [76] (i.e., Swiss-Prot) protein entries created after 2010 (until 2020/04/14), along with their respective sequences, annotations, and annotations scores. We then split these entries by date, 2010-2020, 2015-2020,2018-2020, and 2020 only. For genomic sample benchmarking we selected organisms widely used in microbial community standards. The respective genomes, proteomes, and reference annotations were then downloaded from Uniprot on 2020/05/26 (supplemental **Table 5**). These samples were also used for comparing Mantis to eggNOG-mapper and Prokka.

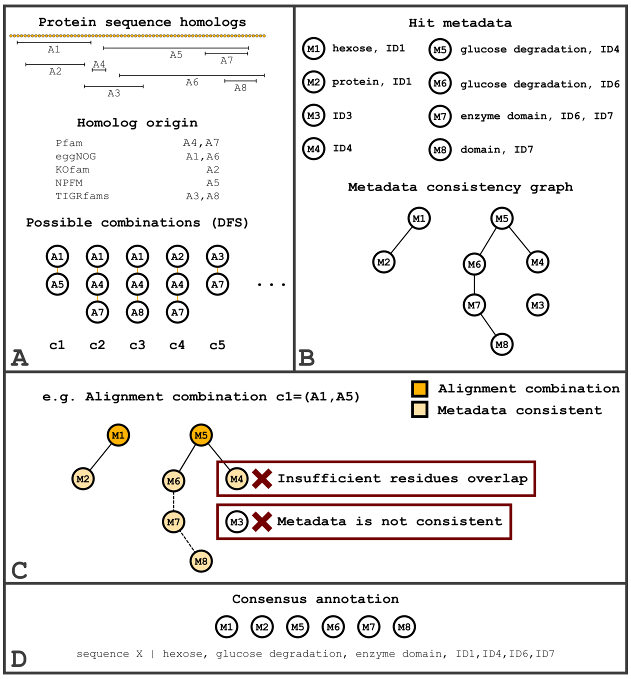

Figure 7. Inter-HMMs hit processing steps. Inter-HMMs hits processing starts by pooling all hits [A1,AN] together (regardless of the reference data source), and generating all the possible (non-overlapping coordinates) combinations [c1,cN] (A). A metadata consistency graph (B) is also built by connecting all nodes [M1,MN] that have intersecting IDs or highly similar descriptions (e.g., A1's metadata M1 is consistent with A2's metadata M2 - shared ID1, and A5's metadata M5 is consistent with A6's metadata M6 - similar description 'glucose degradation'). With this metadata consistency graph, the hit consistency HCN score of each combination is calculated. For c1, for example, a sub-graph containing M1, M5 and all directly connected nodes (only M2 and M6, but not M4, since it has insufficient residues overlap - A4) would be created. The number of nodes in this sub-graph would then be divided by the total number of nodes in the original graph, therefore c1 would have an HCN of $\frac{2+2}{8} = 0.5$. The remaining parameters would then be calculated and the best combination, according to equation 2, would be selected. Finally, if, for example, the best combination is c1, then this combination is expanded by merging all nodes directly or indirectly connected to M1 and M5 in the metadata consistency graph (C) and with sufficient residues overlap (i.e., M2, M6, M7, M8). The expanded combination is then merged into the final consensus annotation (D).

Establishing a test environment

For annotation quality benchmarking, we evaluate each annotation produced by Mantis and check whether it agrees (database IDs intersection) with the respective reference annotation, creating a confusion matrix. We created two main types of test samples, the first consisting exclusively of curated UniProt [76] protein entries (and the respective annotations) which were then split by date of creation (2010-2020, 2015-2020, 2018-2020, 2020). The second type consisting of organism-specific UniProt protein entries, with a mix of curated and automatically generated annotations. Each sequence's reference annotation consists of the UniProt protein function annotations. Each sequence reference annotation and the respective PFA tool's annotation is composed of a set of identifiers (if available: enzyme ECs, Gene ontology (GO) IDs, eggNOG IDs, KEGG orthology IDs, Pfam IDs, and TIGRfam IDs) and functional descriptions. During the benchmark process, each sequence's reference annotation (e.g., "glucose degradation ID1") is compared against the PFA tool (i.e., Mantis, eggNOG-mapper, and Prokka) annotation (e.g., "degrades glucose ID1"). This comparison entails checking whether any of the database IDs present in the reference annotation (i.e., ID1) are also present in the PFA tool annotation (i.e., ID1); if they are, we consider this annotation to be the same. This has some significant limitations: (i) the functional description is the same but the corresponding set of identifiers is not; and (ii) when annotating multiple regions of the protein (which is the case when using Mantis' DFS and heuristic algorithms), it is possible that only one of the annotated regions has IDs that intersect with the respective sequence reference annotation. Unfortunately, due to the different resolutions of the reference HMMs, it is not always possible to understand whether an annotation refers to a specific domain or a partial whole-sequence hit. While a domain-centric benchmark would be feasible for Pfam, the same is not true for the remaining reference HMMs with broader resolutions (e.g., TIGRFams provides general functional annotations). However, as we have previously shown, even when using the BPO algorithm, Mantis has shown to output almost equally high F1 scores. Despite these limitations, since whole-sequence reference annotations contain comprehensive cross-linking with other databases, it provides clear benefits: (i) it fits better for the wide-ranging scopes of the reference data sources, and (ii) allows for a more fair benchmark of the different PFA tools that may use different reference data sources (and thus output annotations with different database IDs). This method then allows for the construction of a confusion matrix, where each pairwise whole sequence annotation comparison (PFA tool/reference annotation) corresponds to a single class. **True-Positives** (TP) occur when the PFA tool generated annotation and the reference annotation share one or more database IDs (e.g., Pfam ID), **False-Positives** (FP) when no database IDs are shared. **False-Negatives** (FN) when the PFA tool does not annotate a protein sequence, but a reference annotation is available, and **True-Negatives** (TN) when the PFA tool does not annotate a protein sequence, and no reference annotation is available. The functional text descriptions are not taken into account during the benchmark, therefore if an annotation has no IDs, we simply consider there is no annotation. Protein sequences annotated with the descriptions "unknown function", "uncharacterized protein", "hypothetical protein" or with Pfam's "domain-unknown-function"/DUF IDs are not taken into account during benchmarking (for reference and PFA tools annotations). In addition, it is also possible that the reference or PFA tool do not have an annotation for a certain sequence. In any of the these three scenarios, if the PFA tool manages to annotate the sequence, this case is classified as potentially new annotation (PNA). Since no ground-truth exists in these scenarios, PNAs are excluded from the confusion matrix classes (not used during any performance metrics) and are only used to calculate the annotation coverage. PNAs can potentially provide novel insight into protein sequences without any previous annotation. Since, by default, most sequences used during benchmarking will have an annotation, TNs, ergo any metrics using TNs (e.g., specificity), are irrelevant.

Annotation coverage is defined here as the number of annotations produced by the PFA tool divided by the total number of protein sequences in a sample $Total_{seqs}$. $Total_{seqs}$ includes sequences with and without a reference annotation (since not all sequences have a reference annotation), the total number of the PFA tool annotations includes TPs, FPs, and PNAs. Annotation coverage is calculated with $\frac{TP+FP+PNA}{Total_{seqs}}$. Numerous metrics can be calculated from the various confusion matrix categories, we considered precision and recall/sensitivity to be among the most important. **Precision** is defined as $\frac{TP}{TP+FP}$ and corresponds to the number of correctly annotated protein sequences, out of all the protein sequences the PFA tool managed to annotate. **Recall** is defined as $\frac{TP}{TP+FN}$ and corresponds to the number of correctly annotated protein sequences, out of all the protein sequences that we know the function of (i.e., protein sequences that have a reference annotation). Both are equally important, a tool with low precision will incorrectly annotate protein sequences, whereas a tool with low recall will not produce sufficient annotations. A way to converge both scores into one is to use the **F1 score**, which is defined as $2 \times \frac{Precision \times Recall}{Precision+Recall}$. Unless otherwise stated, values shown in this paper are shown as absolute values ranging from 0 to 1.

Finally, we benchmarked Mantis against two other PFA tools - eggNOG-mapper and Prokka. For homology search, Mantis uses HMMER [24], for eggNOG-mapper we used the Diamond-based [23] search (as suggested by the authors), and Prokka uses BLAST and HMMER.

All tests ran on an HPC with Dell C6320, 2 * Intel Xeon E5-2680 v4 @ 2.4 GHz [77], each core had 4GB of RAM. Unless specified, all tests ran with 25 cores and 100GB RAM (actual Mantis minimum hardware requirements are much lower). In addition, the same methodology and nomenclature apply to any other benchmarked tools described in this paper. Mantis used HMMER v3.2.1. The local version of eggNOG-mapper used was v2.0.6 with database v5.0.1 found at `https://github.com/eggnogdb/eggnog-mapper/commit/41ec3566ab00fd437f905dfde592c553632a9eae`. The local version of Prokka used was v1.14.6 found at `https://github.com/tseemann/prokka/releases/tag/v1.14.6`.

For details on execution commands please see the supplemental PDF.

Testing different e-value thresholds

Different e-value thresholds were tested: $1e^{-3}$, $1e^{-6}$,$1e^{-9}$,$1e^{-12}$,$1e^{-15}$,$1e^{-18}$,$1e^{-21}$,$1e^{-24}$,$1e^{-27}$,$1e^{-30}$, and a dynamic threshold. The dynamic threshold was set according to the query sequence length, which was previously shown to provide better results with BLAST [34]. For the dynamic threshold, for sequences with less than 150 amino acids, the e-value threshold was set to $1e^{-10}$, if above 150 and below 250, $1e^{-\frac{sequence_{length}}{10}}$, and if above 250, $1e^{-25}$. The UniProt 2010-2020 sample was then annotated with all the different e-value thresholds, and each output was compared to the reference annotations.

Testing hit processing algorithms

In order to understand whether the different hit processing algorithms resulted in statistically significant differences in F1 scores, we created 5000 randomized synthetic samples with 5000 sequences each, which were randomly selected from the 2010-2020 UniProt sample. Per algorithm, we compared the Mantis annotations of each subset to the reference annotations (to allow for pairwise comparison of each algorithm, the same subsets were used in all algorithms). This resulted in a list of confusion matrices (5000 per algorithm), from which we calculated the F1 score. We applied the Wilcoxon signed-rank test, with the H_0: no differences in F1 score between the tested algorithms. As a non-parametric test, this test makes no assumptions on the distribution of the data. A pairwise comparison was done between DFS and the other algorithms: (i) DFS and heuristic, and (ii) DFS and BPO.

Availability of source code and requirements

- Project name: Mantis
- Project home page: `https://github.com/PedroMTQ/mantis`
- Operating system: Linux
- Programming language: Python
- Other requirements: Python 3+, HMMER 3+, and several Python packages (please see the provided environment for a full list)
- License: MIT license at `https://github.com/PedroMTQ/mantis/blob/master/LICENSE`
- RRID: SCR_021001
- Biotools ID: mantis_pfa

Availability of supporting data and materials

The data and code supporting the results of this article are available at `https://git-r3lab.uni.lu/pedro.queiros/mantis_supplements`. The supplemental pdf "supplements.pdf" contains: (i) discussion on how the e-value threshold may change Mantis' output, (ii) execution commands, and (iii) information on how the similarity analysis was performed. The *supplements.xlsx* file contains all tables referenced in this article. The first sheet *ToC* contains the table of contents. An archival copy of the code and supporting data is available via the GigaScience repository, GigaDB [78].

Declarations

List of abbreviations

BPO - best prediction only
CE - combination e-value
DFS - depth first search
EC - enzyme commission
FP - false-positives
FN - false-negatives
GFS - glacier-fed stream sediment
GO - gene ontology
HC - average hit coverage
HCN - hit consistency
HMM - hidden Markov Models
HMMW - average reference HMMs weight
HPC - high-performance computing
ID - database identifier
KO - KEGG orthology
MQ - metadata quality
NLP - natural language processing
NPFM - NCBI protein family models
PFA - protein function annotation
PNA - potentially new annotation
RAM - random access memory
TC - total coverage
TN - true-negatives
TP - true-positives
TSA - taxa-specific annotation
TSHMM - taxa-specific HMM

Competing Interests

The authors declare that they have no competing interests.

Funding

Supported by the Luxembourg National Research Fund PRIDE17/11823097.

Author's Contributions

Author contributions according to the contributor roles taxonomy CRediT was as follows: Conceptualization: P.Q. and P.M.; Data curation: P.Q.; Formal Analysis: P.Q.; Funding acquisition: P.W. and P.M.; Investigation: P.Q.; Methodology: P.Q. and P.M.; Project administration: P.Q. and P.M.; Resources: P.Q.; Software: P.Q.; Supervision: P.M. and P.W.; Validation: P.Q. (lead), F.D., and O.H.; Visualization: P.Q.; Writing – original draft: P.Q. (lead), and P.M.; Writing – review & editing: P.Q., P.M., F.D. , O.H., and P.W.. All authors proof-read and approved of the content in this research paper.

Acknowledgements

The experiments presented in this paper were carried out using the HPC facilities of the University of Luxembourg [77]. P.W. acknowledges the European Research Council (ERC-CoG 863664). We would like to thank Tomila Litvishko for proofreading this research paper. We would like to acknowledge all the creators of the reference data and software used by Mantis, building upon the complementary knowledge of others truly moves the field forward.

References

1. Segata N, Boernigen D, Tickle TL, Morgan XC, Garrett WS, Huttenhower C. Computational meta'omics for microbial community studies. Molecular Systems Biology 2013;9.
2. Muller E, Glaab E, May P, Vlassis N, Wilmes P. Condensing the omics fog of microbial communities. Trends in microbiology 2013 06;21.
3. Whisstock JC, Lesk AM. Prediction of protein function from protein sequence and structure. Quarterly Reviews of Biophysics 2003;36(3):307–340.
4. Arias C, Weisburd B, Stern-Ginossar N, Mercier A, Madrid AS, Bellare P, et al. KSHV 2.0: A Comprehensive Annotation of the Kaposi's Sarcoma-Associated Herpesvirus Genome Using Next-Generation Sequencing Reveals Novel Genomic and Functional Features. PLOS Pathogens 2014;10(1):e1003847.
5. Chapel A, Kieffer-Jaquinod S, Sagné C, Verdon Q, Ivaldi C, Mellal M, et al. An Extended Proteome Map of the Lysosomal Membrane Reveals Novel Potential Transporters. Molecular & Cellular Proteomics 2013;12(6):1572–1588.

6. Iorizzo M, Senalik DA, Grzebelus D, Bowman M, Cavagnaro PF, Matvienko M, et al. De novo assembly and characterization of the carrot transcriptome reveals novel genes, new markers, and genetic diversity. BMC Genomics 2011;12(1):389.
7. Heintz-Buschart A, May P, Laczny CC, Lebrun LA, Bellora C, Krishna A, et al. Integrated multi-omics of the human gut microbiome in a case study of familial type 1 diabetes. Nature Microbiology 2016;2(1):1–13. Number: 1 Publisher: Nature Publishing Group.
8. Mason OU, Scott NM, Gonzalez A, Robbins-Pianka A, Bælum J, Kimbrel J, et al. Metagenomics reveals sediment microbial community response to Deepwater Horizon oil spill. The ISME Journal 2014;8(7):1464–1475.
9. Pasolli E, Asnicar F, Manara S, Zolfo M, Karcher N, Armanini F, et al. Extensive Unexplored Human Microbiome Diversity Revealed by Over 150,000 Genomes from Metagenomes Spanning Age, Geography, and Lifestyle. Cell 2019;176(3):649–662.e20.
10. Sureyya Rifaioglu A, Doğan T, Jesus Martin M, Cetin-Atalay R, Atalay V. DEEPred: Automated Protein Function Prediction with Multi-task Feed-forward Deep Neural Networks. Scientific Reports 2019;9(1):7344.
11. Vazquez A, Flammini A, Maritan A, Vespignani A. Global protein function prediction from protein-protein interaction networks. Nature Biotechnology 2003;21(6):697–700.
12. Borgwardt KM, Ong CS, Schönauer S, Vishwanathan SVN, Smola AJ, Kriegel HP. Protein function prediction via graph kernels. Bioinformatics 2005;21:i47–i56.
13. Steinegger M, Meier M, Mirdita M, Vöhringer H, Haunsberger SJ, Söding J. HH-suite3 for fast remote homology detection and deep protein annotation. BMC Bioinformatics 2019;20(1):473.
14. Seemann T. Prokka: rapid prokaryotic genome annotation. Bioinformatics 2014;30(14):2068–2069.
15. Huerta-Cepas J, Forslund K, Coelho LP, Szklarczyk D, Jensen LJ, von Mering C, et al. Fast Genome-Wide Functional Annotation through Orthology Assignment by eggNOG-Mapper. Molecular Biology and Evolution 2017;34(8):2115–2122.
16. Aziz RK, Bartels D, Best AA, DeJongh M, Disz T, Edwards RA, et al. The RAST Server: Rapid Annotations using Subsystems Technology. BMC Genomics 2008;9(1):75.
17. Ryu JY, Kim HU, Lee SY. Deep learning enables high-quality and high-throughput prediction of enzyme commission numbers. Proceedings of the National Academy of Sciences 2019;116(28):13996–14001.
18. Zhao B, Hu S, Li X, Zhang F, Tian Q, Ni W. An efficient method for protein function annotation based on multilayer protein networks. Human Genomics 2016;10.
19. Szklarczyk D, Gable AL, Lyon D, Junge A, Wyder S, Huerta-Cepas J, et al. STRING v11: protein-protein association networks with increased coverage, supporting functional discovery in genome-wide experimental datasets. Nucleic Acids Research 2019;47:D607–D613.
20. Deng L, Zhong G, Liu C, Luo J, Liu H. MADOKA: an ultra-fast approach for large-scale protein structure similarity searching. BMC Bioinformatics 2019;20(19):662.
21. Overbeek R, Begley T, Butler RM, Choudhuri JV, Chuang HY, Cohoon M, et al. The subsystems approach to genome annotation and its use in the project to annotate 1000 genomes. Nucleic Acids Research 2005;33(17):5691–5702.
22. Altschul SF, Gish W, Miller W, Myers EW, Lipman DJ. Basic local alignment search tool. Journal of Molecular Biology 1990;215(3):403–410.
23. Buchfink B, Xie C, Huson DH. Fast and sensitive protein alignment using DIAMOND. Nature Methods 2015;12(1):59–60.
24. Roberts Eddy S, HMMER; 2020.
25. Jones P, Binns D, Chang HY, Fraser M, Li W, McAnulla C, et al. InterProScan 5: genome-scale protein function classification. Bioinformatics 2014;30(9):1236–1240.
26. Lohse M, Nagel A, Herter T, May P, Schroda M, Zrenner R, et al. Mercator: a fast and simple web server for genome scale functional annotation of plant sequence data. Plant, Cell & Environment 2014;37(5):1250–1258.
27. Wu S, Zhu Z, Fu L, Niu B, Li W. WebMGA: a customizable web server for fast metagenomic sequence analysis. BMC genomics 2011;12.
28. Mitchell AL, Almeida A, Beracochea M, Boland M, Burgin J, Cochrane G, et al. MGnify: the microbiome analysis resource in 2020. Nucleic Acids Research 2020;48:D570–D578.
29. Keegan KP, Glass EM, Meyer F. MG-RAST, a Metagenomics Service for Analysis of Microbial Community Structure and Function. Methods in Molecular Biology (Clifton, NJ) 2016;1399:207–233.
30. Pfeiffer F, Oesterhelt D. A Manual Curation Strategy to Improve Genome Annotation: Application to a Set of Haloarchael Genomes. Life 2015;5(2):1427–1444.
31. Yeats C, Redfern OC, Orengo C. A fast and automated solution for accurately resolving protein domain architectures. Bioinformatics 2010;26(6):745–751.
32. Ekman D, Bjorklund AK, Frey-Skott J, Elofsson A. Multi-domain Proteins in the Three Kingdoms of Life: Orphan Domains and Other Unassigned Regions. Journal of Molecular Biology 2005;348(1):231–243.
33. Lees JG, Lee D, Studer RA, Dawson NL, Sillitoe I, Das S, et al. Gene3D: Multi-domain annotations for protein sequence and comparative genome analysis. Nucleic Acids Research 2014;42:D240–D245.
34. Treiber ML, Taft DH, Korf I, Mills DA, Lemay DG. Pre- and post-sequencing recommendations for functional annotation of human fecal metagenomes. BMC Bioinformatics 2020;21(1):74.
35. Schnoes AM, Brown SD, Dodevski I, Babbitt PC. Annotation Error in Public Databases: Misannotation of Molecular Function in Enzyme Superfamilies. PLoS Computational Biology 2009;5(12).
36. Friedberg I. Automated protein function prediction—the genomic challenge. Briefings in Bioinformatics 2006;7(3):225–242.
37. Araujo FA, Barh D, Silva A, Guimarães L, Ramos RTJ. GO FEAT: a rapid web-based functional annotation tool for genomic and transcriptomic data. Scientific Reports 2018;8(1):1794.
38. Klimke W, O'Donovan C, White O, Brister JR, Clark K, Fedorov B, et al. Solving the Problem: Genome Annotation Standards before the Data Deluge. Standards in Genomic Sciences 2011;5(1):168–193.
39. Standardizing data. Nature Cell Biology 2008;10(10):1123–1124.
40. Gaikwad SV, Chaugule A, Patil P. Text mining methods and techniques. International Journal of Computer Applications 2014;85(17).
41. Wang S, Ma J, Yu MK, Zheng F, Huang EW, Han J, et al. Annotating gene sets by mining large literature collections with protein networks. Pacific Symposium on Biocomputing Pacific Symposium on Biocomputing 2018;23:602–613.
42. Pesquita C, Faria D, Falcão AO, Lord P, Couto FM. Semantic similarity in biomedical ontologies. PLoS computational biology 2009;5(7):e1000443.
43. Zeng Z, Shi H, Wu Y, Hong Z. Survey of Natural Language Processing Techniques in Bioinformatics. Computational and Mathematical Methods in Medicine 2015;2015:674296.
44. Slater LT, Bradlow W, Ball S, Hoehndorf R, Gkoutos

GV. Improved characterisation of clinical text through ontology-based vocabulary expansion. bioRxiv 2020;p. 2020.07.10.197541.
45. Huang CC, Lu Z. Community challenges in biomedical text mining over 10 years: success, failure and the future. Briefings in Bioinformatics 2016;17(1):132–144.
46. Benabderrahmane S, Smail-Tabbone M, Poch O, Napoli A, Devignes MD. IntelliGO: a new vector-based semantic similarity measure including annotation origin. BMC Bioinformatics 2010;11:588.
47. Peng J, Uygun S, Kim T, Wang Y, Rhee SY, Chen J. Measuring semantic similarities by combining gene ontology annotations and gene co-function networks. BMC bioinformatics 2015;16.
48. Liu M, Thomas PD. GO functional similarity clustering depends on similarity measure, clustering method, and annotation completeness. BMC bioinformatics 2019;20(1):155.
49. Daraselia N, Yuryev A, Egorov S, Mazo I, Ispolatov I. Automatic extraction of gene ontology annotation and its correlation with clusters in protein networks. BMC bioinformatics 2007;8:243.
50. Ehsani R, Drabløs F. TopoICSim: a new semantic similarity measure based on gene ontology. BMC bioinformatics 2016;17(1):296.
51. Kramer M, Dutkowski J, Yu M, Bafna V, Ideker T. Inferring gene ontologies from pairwise similarity data. Bioinformatics (Oxford, England) 2014;30(12):i34–42.
52. Promponas VJ, Iliopoulos I, Ouzounis CA. Annotation inconsistencies beyond sequence similarity-based function prediction – phylogeny and genome structure. Standards in Genomic Sciences 2015;10.
53. Ellens KW, Christian N, Singh C, Satagopam VP, May P, Linster CL. Confronting the catalytic dark matter encoded by sequenced genomes. Nucleic Acids Research 2017;45(20):11495–11514.
54. Aramaki T, Blanc-Mathieu R, Endo H, Ohkubo K, Kanehisa M, Goto S, et al. KofamKOALA: KEGG Ortholog assignment based on profile HMM and adaptive score threshold. Bioinformatics 2020;36(7):2251–2252.
55. El-Gebali S, Mistry J, Bateman A, Eddy SR, Luciani A, Potter SC, et al. The Pfam protein families database in 2019. Nucleic Acids Research 2019;47:D427–D432.
56. Huerta-Cepas J, Szklarczyk D, Heller D, Hernández-Plaza A, Forslund SK, Cook H, et al. eggNOG 5.0: a hierarchical, functionally and phylogenetically annotated orthology resource based on 5090 organisms and 2502 viruses. Nucleic Acids Research 2019;47:D309–D314.
57. Lu S, Wang J, Chitsaz F, Derbyshire MK, Geer RC, Gonzales NR, et al. CDD/SPARCLE: the conserved domain database in 2020. Nucleic acids research 2020;48(D1):D265–D268.
58. Haft DH, Selengut JD, Richter RA, Harkins D, Basu MK, Beck E. TIGRFAMs and Genome Properties in 2013. Nucleic Acids Research 2013;41:D387–D395.
59. Queirós P, Mantis - Wiki. GitHub; 2020. https://github.com/PedroMTQ/mantis/wiki.
60. Hyatt D, Chen GL, LoCascio PF, Land ML, Larimer FW, Hauser LJ. Prodigal: prokaryotic gene recognition and translation initiation site identification. BMC Bioinformatics 2010;11:119.
61. Delogu F, fdelogu/SEM1b-Multiomics; 2019.
62. Kunath BJ, Delogu F, Naas AE, Arntzen M, Eijsink VGH, Henrissat B, et al. From proteins to polysaccharides: lifestyle and genetic evolution of Coprothermobacter proteolyticus. The ISME Journal 2019;13(3):603–617.
63. Busi SB, Pramateftaki P, Brandani J, Fodelianakis S, Peter H, Halder R, et al. Optimised biomolecular extraction for metagenomic analysis of microbial biofilms from high-mountain streams. PeerJ 2020 Oct;8:e9973. https://doi.org/10.7717/peerj.9973.
64. Sunagawa S, Coelho LP, Chaffron S, Kultima JR, Labadie K, Salazar G, et al. Structure and function of the global ocean microbiome. Science 2015;348(6237). https://science.sciencemag.org/content/348/6237/1261359.
65. Sigrist CJA, de Castro E, Cerutti L, Cuche BA, Hulo N, Bridge A, et al. New and continuing developments at PROSITE. Nucleic Acids Research 2013;41:D344–347.
66. Mooney MA, Nigg JT, McWeeney SK, Wilmot B. Functional and Genomic Context in Pathway Analysis of GWAS Data. Trends in genetics : TIG 2014;30(9):390–400.
67. Mavromatis K, Chu K, Ivanova N, Hooper SD, Markowitz VM, Kyrpides NC. Gene Context Analysis in the Integrated Microbial Genomes (IMG) Data Management System. PLOS ONE 2009;4(11):e7979.
68. Hannigan GD, Prihoda D, Palicka A, Soukup J, Klempir O, Rampula L, et al. A deep learning genome-mining strategy for biosynthetic gene cluster prediction. Nucleic Acids Research 2019 08;47(18):e110–e110.
69. Parks DH, Chuvochina M, Waite DW, Rinke C, Skarshewski A, Chaumeil PA, et al. A standardized bacterial taxonomy based on genome phylogeny substantially revises the tree of life. Nature Biotechnology 2018;36(10):996–1004.
70. Parks DH, Chuvochina M, Chaumeil PA, Rinke C, Mussig AJ, Hugenholtz P. A complete domain-to-species taxonomy for Bacteria and Archaea. Nature Biotechnology 2020;38(9):1079–1086.
71. Buell R, Deutschbauer A, Adin D, Ronning C, Breaking the Bottleneck of Genomes: Understanding Gene Function Across Taxa; 2018.
72. Sunagawa S, Coelho LP, Chaffron S, Kultima JR, Labadie K, Salazar G, et al. Structure and function of the global ocean microbiome. Science 2015;348(6237). Publisher: American Association for the Advancement of Science Section: Research Article.
73. Mangul S, Mosqueiro T, Abdill RJ, Duong D, Mitchell K, Sarwal V, et al. Challenges and recommendations to improve the installability and archival stability of omics computational tools. PLOS Biology 2019 06;17(6):1–16. https://doi.org/10.1371/journal.pbio.3000333.
74. Kaur N, Garg D. Analysis of the Depth First Search Algorithms. Data mining and knowledge engineering 2012;4:37–41.
75. Behnel S, Bradshaw R, Citro C, Dalcin L, Seljebotn DS, Smith K. Cython: The Best of Both Worlds. Computing in Science Engineering 2011;13(2):31–39.
76. UniProt: a worldwide hub of protein knowledge. Nucleic Acids Research 2019;47:D506–D515.
77. Varrette S, Bouvry P, Cartiaux H, Georgatos F. Management of an Academic HPC Cluster: The UL Experience 2014;https://hpc.uni.lu.
78. Queirós P, Delogu F, Hickl O, May P, Wilmes P, Supporting data for "Mantis: flexible and consensus-driven genome annotation". GigaScience Database; 2021. http://dx.doi.org/10.5524/100903.

Supplemental tables

Click here to access/download
Supplementary Material
supplements.xlsx

Supplemental PDF

Click here to access/download
Supplementary Material
supplemental.pdf

Author information

Click here to access/download
Supplementary Material
author_information.txt
