# Supplementary material for: Mantis: flexible and consensus-driven genome annotation
Source: Gigascience. 2021 Jun 2;10(6):giab042. doi: 10.1093/gigascience/giab042 (PMC8170692; doi:10.1093/gigascience/giab042)
Supplement: giab042_Supplemental_Files [file giab042_supplemental_files.zip › supplemental.pdf]

Impact of the e-value threshold

As an initial quality control of Mantis, we tested different static e-value thresholds and a dynamic threshold to set a default HMMER e-value threshold within Mantis. Interestingly, we saw (supplemental **Table 1**) that a stricter/lower e-value threshold did not necessarily lead to a higher F1 score. By limiting the amount of hits output by HMMER, the e-value threshold will significantly affect the intermediate Mantis' processing steps and therefore its final output.

Naturally, a more stringent e-value threshold results in fewer HMMER hits; this in turn reduces the amount of annotations produced by Mantis, and, by extent, the amount of TPs in the benchmark's confusion matrix.

Unlike TPs, FPs do not necessarily decrease with a lower e-value threshold. For example, the confusion matrix for the sample Uniprot 2010-2020 annotated with an e-value threshold of $1e^{-6}$ and $1e^{-30}$ had 12360 and 12199 FPs, respectively. This goes according to the expectation that a more strict e-value threshold results in better HMMER hits. However, using an e-value threshold of $1e^{-21}$ resulted in 12497 FPs, which contradicts the expected trend (lower threshold would, in theory, equal to less FPs). This occurs because of Mantis' quality control during consensus generation, in particular due to the fact that Mantis attempts to find different reference data sources that point towards the same function. When a higher e-value threshold is used, more hits are available, and thus finding multiple hits that point towards the same function is "easier" than when the available solution space is more limited.

As an example, we selected a protein sequence that has the following reference functional annotation IDs:

```
go:0000160   go:0003677   go:0006355   kegg_ko:K07774   pfam:PF00072   pfam:PF00486
```

Note that while several variables are taken into account for hit combination scoring, for simplicity here we will only refer to the e-value. We then extracted the Mantis functional annotation for the same sequence in the Uniprot 2010-2020 sample, first when using an e-value of $1e^{-6}$ and secondly when using an e-value of $1e^{-21}$. The sample annotated with an e-value threshold of $1e^{-6}$ resulted in the following functional annotations (from the *integrated_annotation.tsv* file):

```
Database          HMM hit          e-value  |  Annotation
tigrfam_merged    TIGR01387        2.2e-50  |  tigrfam:TIGR01387
Pfam-A            Response_reg     6.5e-25  |  pfam:PF00072
Pfam-A            Trans_reg_C      5.7e-19  |  pfam:PF00486 description:Transcriptional
                                               regulatory protein, C terminal
NOGG_merged       2SE5K            2.3e-63  |  description:Transcriptional
                                               regulatory protein, C terminal
NCBIG_merged      cztR_silR_copR   2.2e-50  |  tigrfam:TIGR01387
kofam_merged      K02483           2.9e-69  |  cog:COG0745 go:0000156 kegg_ko:K02483
```

For this threshold, the *consensus_annotation.tsv* file contained the HMM hits $2SE5K$ and $Trans\_reg\_C$. The hit with the best e-value was $K02483$, however since $2SE5K$ and $Trans\_reg\_C$ shared the description "*Transcriptional regulatory protein, C terminal*", these two hits were chosen and merged as the consensus annotation. This consensus annotation shares the ID $PF00486$ with the reference annotation and is therefore marked as a TP.

The same sequence but now using using an e-value threshold of $1e^{-21}$:

```
Database          HMM hit          e-value  |  Annotation
tigrfam_merged    TIGR01387        2.2e-50  |  tigrfam:TIGR01387
Pfam-A            Response_reg     6.5e-25  |  pfam:PF00072
NOGG_merged       2SE5K            2.3e-63  |  description:Transcriptional
                                               regulatory protein, C terminal
NCBIG_merged      cztR_silR_copR   2.2e-50  |  tigrfam:TIGR01387
kofam_merged      K02483           2.9e-69  |  cog:COG0745 go:0000156 kegg_ko:K02483
```

In this case, the hit $Trans\_reg\_C$ is no longer available since it had an e-value of 5.7e-19, which is above the e-value threshold. Consequently, the *consensus_annotation.tsv* file contained instead the HMM hit $K02483$, since, among all available hits, it's the one with the lowest e-value. The consensus annotation now does not share an ID with the reference annotation and is therefore marked as a FP.

The e-value threshold can also have an impact on the choice of the best combination. Since the combination e-value is calculated by scaling with log10 and minmax, different e-value thresholds will result in different minmax values, and, by extent, different scores for the same combination of hits. For example, if we find 3 hits when using an e-value threshold of $1e^{-3}$:

- hit 1 with e-value 1e-5
- hit 2 with e-value 1e-15
- hit 3 with e-value 1e-10

By applying log10 and minmax scale to each hit we get:

- hit 1 with minmax log10 e-value of 0
- hit 2 with minmax log10 e-value of 1
- hit 3 with minmax log10 e-value of 0.5

Now with an e-value threshold of $1e^{-6}$:

- hit 2 with e-value 1e-15
- hit 3 with e-value 1e-10

By applying log10 and minmax scale to each hit we get:

- hit 2 with minmax log10 e-value of 1
- hit 3 with minmax log10 e-value of 0

In the first scenario, should hit 3 score well in the other ***combination score*** variables, it can still be picked above hit 2 (should hit 2 score poorly in the other ***combination score*** variables). In the second scenario, it is highly unlikely that hit 3 will be chosen since it is now has the worst e-value of all the hits.

While these are anecdotal examples, they depict why and how different e-evalue threshold may lead to unexpected results.

Execution commands

Mantis uses HMMER, which runs via the following command:

```
$ hmmsearch --domtblout output.domtblout
--domE e_value --notextw dataset.hmm sample.faa
```

When using the DFS or heuristic algorithm, Mantis uses HMMER's independent e-value as a confidence score, and, when using the BPO algorithm, it uses the full sequence e-value (see `http://eddylab.org/software/hmmer/Userguide.pdf` pp.71-72). The e-value threshold set in the hmmsearch will be the same for all algorithms, however, when running Mantis with the DFS or heuristic, the threshold variable will be *domE*, whereas for BPO, it will be *E*. The e-value type and threshold chosen reflect the fact that the DFS and heuristic algorithm may capture multiple hits per protein, whereas the BPO only captures one hit per sequence. Mantis was executed with the following command:

```
$ python mantis run_mantis -t sample.faa -od NCBI_ID
```

We ran Mantis with (with $-od$) and without taxonomy information (without $-od$). Mantis also ran without the eggNOG dataset by using a modified $MANTIS.config$ file where $nog\_hmm\_folder = NA$.
eggNOG-mapper was executed with the following command:

```
$ python emapper.py -i sample.faa -m diamond
```

We ran eggNOG-mapper with and without taxonomy information, when running it with taxonomy information we added *tax_scope*. Taxonomic lineage correspondeds to a list of NCBI IDs, from the most specific sample's NCBI ID to the least specific. For example, a *Escherichia coli* would run with:

```
$ python emapper.py -i sample.faa -m diamond
--tax_scope 561,1236,1224
```

Taxonomic lineages were calculated with $MANTIS\_Assembler.get\_organism\_lineage$ and then reversed (Mantis outputs least specific taxon $->$ most specific taxon, but eggNOG-mapper requires the opposite). We then removed taxon IDs that were not present in the eggNOG reference.
Prokka was executed with the following command:

```
$ prokka sample.fasta
```

When comparing Prokka to the reference annotation, we aligned (using Diamond) the protein sequences produced by Prokka to the respective reference protein sequences, getting, for each sequence, the best scoring alignment. For example, for *Eschrichia coli* this was done with the following lines of code:

```
$ diamond makedb --in ecoli_k12.faa -d ecoli_k12
$ diamond blastp -d ecoli_k12.dmnd -q prokka.faa
-o alignment_prokka.m8 --more-sensitive
```

On average 3.53% of the gene-called sequences did not align to the reference sequences.
Wilcoxon signed rank test were performed using the python package Scipy [12], with the function "scipy.stats.wilcoxon".

Consensus between free text functional descriptions

The *consensus between free text functional descriptions* is done by comparing the free-text descriptions from two sources and evaluating whether these are similar. This starts with parsing and pre-processing of the text within the functional descriptions. It is followed by the lexical classification of the different processed words, so that irrelevant words are not considered in the next steps. This classification is done via a tagger [7] independent of context. This tagger uses the Wordnet lexicon [13] to identify the most common lexical category of any given word. Wordnet is used as it provides one of the most comprehensive lexical databases in the English language. To adjust this lexicon to biological data, gene ontologies [2, 10] are parsed, processed, and added as nouns to the tagger. If there are still untagged words, these are contextually classified with a pre-trained Perceptron tagger [8, 1]. Finally, the words tagged as determiners, pronouns, particles, or conjunctions are removed. Each word is then given a weight so that less specific words (e.g. "protein") have a lower weight, and vice versa. We chose the metric Term Frequency-Inverse Document Frequency (*TF-IDF*) as it is a good metric for scoring how important a word is in a document (in the current context, documents are the functional descriptions) relative to the entire corpus (reference collection of functional descriptions), and has been successfully used in the past [3, 5, 9, 6]. We pre-calculated a frequency table of all the words from a collection of 561.911 reviewed proteins from Swiss-Prot [11] (as of 2020/04/14). With this frequency table, the IDF metric (corpus-wide/global weight of each word) can be calculated, such that words that appear in too many functional descriptions in the corpus are less important. TF is a local metric specific to the functional description being analysed, weighing words such that more frequent words in an functional description are given a higher weight. Finally, *TF-IDF* is calculated with the following equation:

$$\frac{NW}{TW} \times \frac{TC}{NC} \tag{1}$$

where NW is the amount of times a word appears in an functional description, TW the total amount of words in an functional description, TC the total amount of functional descriptions in the corpus (561.911), and NC the total amount of times a certain word appears in the corpus. The *TF-IDF* score is then locally scaled so that we can better understand which words are more relevant within the current functional description. After these steps we obtain a TF-IDF scaled vector for each functional description. Finally, for similarity analysis, we calculate the cosine distance [4] between all the functional description TF-IDF scaled vectors (which would come from different data sources), effectively measuring how similar these two vectors are. Should the words they contain and their importance within the functional description be similar, these are given a high similarity score. A similarity threshold was determined by empirically evaluating a set of 1000 pairwise descriptions. Documents above the threshold 0.9 were considered to be describing the same function.

To note that during consensus generation through descriptions, should the functional annotation contain GO IDs, Mantis adds the description of these GO IDs (from the go.obo file) to the set of descriptions of the functional annotation. For example, a functional annotation that contains the description "methyltransferase activity" may form a consensus with a functional annotation that contains the ID GO:0008168 since GO:0008168's description is "methyltransferase activity". To avoid non-specific GO IDs we do not use this method when the functional annotation contains more than 10 GO IDs.

References

[1] *A Good Part-of-Speech Tagger in about 200 Lines of Python.* 2013.

[2] Michael Ashburner et al. "Gene Ontology: tool for the unification of biology". In: *Nature genetics* 25.1 (2000), pp. 25–29. ISSN: 1061-4036. DOI: 10.1038/75556.

[3] Sidahmed Benabderrahmane et al. "IntelliGO: a new vector-based semantic similarity measure including annotation origin". In: *BMC Bioinformatics* 11 (2010), p. 588. ISSN: 1471-2105. DOI: 10.1186/1471-2105-11-588.

[4] Jiawei Han, Jian Pei, and Micheline Kamber. *Data mining: concepts and techniques*. Elsevier, 2011. ISBN: 0-12-381480-4.

[5] Miao Hao and Ke Fan. "A Method for Calculating the Similarity of TF - IDF Texts for Synonyms in Biomedical Domains". In: 2017 5th International Conference on Frontiers of Manufacturing Science and Measuring Technology (FMSMT 2017). Atlantis Press, 2017, pp. 578–583. ISBN: 978-94-6252-331-9. DOI: 10.2991/fmsmt-17.2017.117.

[6] Yue Huang, Mingxin Gan, and Rui Jiang. "Ontology-Based Genes Similarity Calculation with TF-IDF". In: vol. 7473. 2012, pp. 600–607. DOI: 10.1007/978-3-642-34062-8_78.

[7] Slav Petrov, Dipanjan Das, and Ryan McDonald. "A Universal Part-of-Speech Tagset". In: *arXiv:1104.2086 [cs]* (2011). (Visited on 06/25/2020).

[8] Frankie Roberston. *Averaged perceptron tagger*. 2016.

[9] Georgi Tancev. *Mining and Classifying Medical Documents*. Medium. Library Catalog: towardsdatascience.com. 2019.

[10] "The Gene Ontology Resource: 20 years and still GOing strong". In: *Nucleic Acids Research* 47 (D1 2019), pp. D330–D338. ISSN: 0305-1048. DOI: 10.1093/nar/gky1055.

[11] "UniProt: a worldwide hub of protein knowledge". In: *Nucleic Acids Research* 47 (D1 2019), pp. D506–D515. ISSN: 0305-1048. DOI: 10.1093/nar/gky1049.

[12] Pauli Virtanen et al. "SciPy 1.0: Fundamental Algorithms for Scientific Computing in Python". In: *Nature Methods* 17 (2020), pp. 261–272. DOI: 10.1038/s41592-019-0686-2.

[13] *WordNet — A Lexical Database for English*. 2010.
