# Supplementary material for: Mantis: flexible and consensus-driven genome annotation
Source: Gigascience. 2021 Jun 2;10(6):giab042. doi: 10.1093/gigascience/giab042 (PMC8170692; doi:10.1093/gigascience/giab042)
Supplement: giab042_Response_to_Reviewer_Comments_Original_Submission [file giab042_response_to_reviewer_comments_original_submission.pdf]

Author's Response To Reviewer Comments

Close

We have provided a PDF document entitled "reviewer_response.pdf" where we list all the editor and reviewer comments, alongside the respective response.
Regardless, we also provide the same documents below:

We would like to thank both the editor and the reviewers for the very in-depth review. Overall, we have tried to explain ou methods better in the revised version of the manuscript by providing more examples and figures. We have also repeated analyses due to the proposed (and some additional) changes in Mantis. To provide better explanations to our original stud have divided each review into bullet points to address each comment separately. Each comment is marked as "RXCY", X b reviewer number and Y the comment number. R0 corresponds to the GigaScience editor comments, R1 to the reviewer Ka Ross and R2 to the reviewer Carlos Cantalapiedra.
In response to the reviewer comments, we have made some substantial changes in the manuscript, benchmarking and implementation of Mantis. Major changes are listed below:
• We have added a new reference data source - NCBI protein family models, from this link https://ncbiinsights.ncbi.nlm.nih.gov/tag/protein-family-model/. These are referred to as NPFM in the manuscript, and, al eggNOG, are also used for taxa-specific annotation;
• The original comparison between the reference annotations and the annotations produced by the several tools used text similarity analysis and intersection of IDs. The new benchmark now only uses the intersection of IDs, as we believe it to b error-prone;
• The following new subsubsections have been added to the subsection "Initial quality control" in the section "Analysis": "I of hit processing algorithms", "Contribution of the different reference data sources", "Impact of consensus generation", an processing approximation";
• The following sections have been added: "Annotating metagenomes", "Computational efficiency" (moved from the supplements);
• The following figures have been added: "Figure 2. Homolog selection for the three hit processing algorithms in Mantis" a "Figure 7. Inter-HMMs hit processing steps". Please keep in mind figure numbers may have changed from the past manus version;
• The old supplemental PDF has been removed. The new supplemental PDF includes the following sections: "Impact of the threshold", "Execution commands", and "Consensus between free text functional descriptions".

Some sections have also been rewritten and other minor changes were made. For easier comparison, with our resubmissio include a PDF comparison of the old and revised manuscripts "version_comparison.pdf" and a version of the manuscript w "manuscript_lines.pdf".
Whenever applicable, we also include transcripts of the manuscript in our response to the reviewers.

R0C1:
register any new software application in the bio.tools and SciCrunch.org databases to receive RRID (Research Resource Identification Initiative ID) and biotoolsID identifiers, and include these in your manuscript.
Response:
Mantis has been submitted to both databases, the corresponding IDs have been added to the section "Availability of sourc and requirements".

Reviewer #1

R1C1:
presentation of the method and its testing needs clarification to enable understanding of exactly how the tool works and t properly evaluate its performance. Providing specific examples in a few key places would help a lot.
Response:
We thank the reviewer for this important point. We have expanded the "Multiple predictions per protein" section, explainin each score is calculated and how combinations of hits are generated. Please see the rewritten "Multiple predictions per pro section.

In addition, we have also expanded the section "Using multiple reference datasets" and added a new figure (7) for inter-H[illegible] processing, where we explain each step and give specific examples.

R1C2:
Figures 5 and 6 make it clear that Mantis can find matches to multiple non-overlapping regions of the protein (multiple do[illegible] matches). It seems plausible that sometimes Mantis would identify some but not all of the domains in a manually annotat[illegible] SwissProt entry or might identify some domains correctly but then also have one or more false positive domains. However[illegible] calculation of %TP and %FP appears to be done at the whole protein level--"The % TPs and % FPs are, respectively, defin[illegible] the number of TPs and FPs divided by the number of sequences in the sample." How does this calculation take into accour[illegible] multiple matches per protein? An example here would help.
Response:
We thank the reviewer for this very valid comment. Domain-specific benchmarking comes with a set of issues that we can[illegible] address since Mantis uses multiple reference sources. While we agree that this may result in a sub-optimal benchmarking[illegible] believe this compromise to be the only feasible benchmarking solution. However, please also see the new section "Impact[illegible] consensus generation" where we also tested the performance of Mantis with the BPO algorithm and without consensus ge[illegible] (just one prediction per sequence) and showed that even without multiple predictions and multiple references per predicti[illegible] BPO still achieves an F1 score of 0.7627. These results showcase the importance of having a broader pool of hits (due to t[illegible] of multiple references) when choosing the best predictions. As the reviewer mentioned, it is possible that Mantis may ann[illegible] more domains than it should. For example, the protein entry A8D8P8 (https://www.uniprot.org/uniprot/A8D8P8) contains[illegible] domains: Piwi (http://pfam.xfam.org/family/PF02171) and PAZ (https://pfam.xfam.org/family/PF02170). Mantis could theoretically annotate both these domains and an additional domain in a domain-centric benchmark. We could then consi[illegible] Mantis annotated 2 TPs and 1 FP. In the current whole-sequence-centric benchmark this will simply be considered as 1 TP[illegible] the domain-centric approach may seem more plausible, Mantis does not only use the domain-specific Pfam database, inst[illegible] relies on multiple reference sources, most of which have different scopes and wildly different resolutions. Some reference [illegible] focus on whole sequence annotation (i.e. eggNOG), others aim at providing the general functional scope of the protein (i.[illegible] TIGRFAM), and others domain-specific resolution (i.e. Pfam). Most of these references (besides Pfam) have little to no inf[illegible] on domains. Even if domain-specific information were available, we found that Swiss-Prot domain information mostly cros[illegible] with Pfam, which would invalidate the benchmark for sequences annotated with other reference sources (and other tools)[illegible] such different resources, domain-centric benchmarking becomes extremely complex (if feasible at all). On the other hand[illegible] Prot provides comprehensive cross-linking for whole-sequence annotations, which better fits the wide ranging scopes of th[illegible] reference sources used by Mantis. It also allows for a fairer benchmark against other tools. To conclude, while we do agre[illegible] the reviewer, for practical reasons, we have decided whole-sequence benchmarking to be the only option (even if a sub-o[illegible] one). We have also clarified this issue in the manuscript, please see the following transcript in page 11, lines 949-968, in [illegible] subsection "Methods.Establishing a test environment":
This has some significant limitations: (i) the functional description is the same but the corresponding set of identifiers is n[illegible] (ii) when annotating multiple regions of the protein (which is the case when using Mantis' DFS and heuristic algorithms), i[illegible] possible that only one of the annotated regions has IDs that intersect with the respective sequence reference annotation. Unfortunately, due to the different resolutions of the reference HMMs, it is not always possible to understand whether an annotation refers to a specific domain or a partial whole-sequence hit. While a domain-centric benchmark would be feasib[illegible] Pfam, the same is not true for the remaining reference HMMs with broader resolutions (e.g., TIGRFams provides general functional annotations). Despite these limitations, since whole-sequence reference annotations contain comprehensive cro[illegible] linking with other databases, it provides clear benefits: (i) it better fits the wide-ranging scopes of the reference data sour[illegible] and (ii) allows for a more fair benchmark of the different PFA tools that may use different reference data sources (and thu[illegible] annotations with different database IDs).

R1C3:
When creating the consensus annotation, Mantis evaluates whether the annotations from two different resources are simil[illegible] it is confusing that the authors use the word "complementary" to describe the situation where annotations from two resou[illegible] similar. "Consistent" or "confirmatory" might be better word choices. Second, it is unclear what the outcome would be if tw[illegible] resources find hits on two non-overlapping regions. These hits would obvious be very dissimilar in annotation. Would they[illegible] included in the consensus annotation?
Response:
We acknowledge the reviewer's comment. We used the term complementary since cross-linking can add new identifiers to[illegible] final annotation. Nevertheless, we changed the term "complementary" to "consistent" whenever applicable. To answer the[illegible] question, yes, both hits would be kept; an example is now available in the inter-HMMS figure 7.

R1C4:
In the "Establishing a test environment" sub-section of the Methods, it would be helpful to clarify what is meant by "refere[illegible] annotation". Where does it come from and what exactly does it consist of?
Response:
The reference annotations come from Uniprot, it has now been specified in the "Establishing a test environment" section w[illegible] exactly they contain and where they come from. Please see the following transcript in page 10, lines 927-942 in the subse[illegible]

”Methods.Establishing a test environment”:
For annotation quality benchmarking, we evaluate each annotation produced by Mantis and check whether it agrees (data
IDs intersection) with the respective reference annotation, creating a confusion matrix. We created two main types of test
samples, the first consisting exclusively of curated UniProt protein entries (and the respective annotations) which were the
by date of creation (2010-2020, 2015-2020, 2018-2020, 2020). The second type consisting of organism-specific UniProt p
entries, with a mix of curated and automatically generated annotations. Each sequence's reference annotation consists of
UniProt protein function annotations.
Each sequence reference annotation and the respective PFA tool's annotation is composed of a set of identifiers (if availab
enzyme ECs, Gene ontology (GO) IDs, eggNOG IDs, KEGG orthology IDs, Pfam IDs, and TIGRfam IDs) and functional
descriptions.

R1C5:
Also, this section says that Mantis annotations that don't match the reference are considered false positives, but it also sa
when Mantis outputs "valid annotation but the reference is either of poor quality or non-existent" that this is considered a
potential new annotation. How exactly is the distinction made between a false positive and potential new annotation?
Response:
A potentially new annotation (PNA) is a protein sequence that has been annotated with a protein function annotation tool
does not have a proper reference annotation (inexistent or of poor quality - e.g. “hypothethical protein”). We do not consi
these to be FPs because Uniprot also contains automatically generated annotations. For this reason, potentially new annot
mostly occur when we annotated the several organism samples, which may contain a large amount of automatically anno
sequences or un-annotated sequences. These PNAs are excluded from the confusion matrix but are included in the annota
coverage calculation. Please see the following transcript in page 11, lines 972-996 in the section ”Methods.Establishing a t
environment”:
True-Positives (TP) occur when the PFA tool generated annotation and the reference annotation share one or more databa
(e.g., Pfam ID), False-Positives (FP) when no database IDs are shared. False-Negatives (FN) when the PFA tool does not a
a protein sequence, but a reference annotation is available, and True-Negatives (TN) when the PFA tool does not annotate
protein sequence, and no reference annotation is available. The functional text descriptions are not taken into account dur
benchmark, therefore if an annotation has no identifiers, we simply consider there is no annotation. As such, it is possible
reference annotation exists, but the PFA tool manages to annotate the sequence; when this is the case this is classified as
potentially new annotation (PNA).
Protein sequences annotated with the descriptions “unknown function”, “uncharacterized protein”, ”hypothetical protein” o
Pfam’s "domain-unknown-function"/DUF IDs are not taken into account during benchmarking (for reference and PFA tools
annotations). In addition, it is also possible that the reference contains no annotation at all. Should the PFA annotate the
sequence, both these cases would correspond to PNAs.
Since no ground-truth exists in these scenarios, PNAs are excluded from the confusion matrix classes (not used during an
performance metrics) and are only used to calculate the annotation coverage. PNAs can potentially provide novel insight in
protein sequences without any previous annotation.

R1C6:
When calculating the similarity threshold (supplemental material), the authors analyzed a group of proteins where the ide
disagreed. Were these cases where the Mantis annotation of a protein sequence had no identifiers in common with the Sw
annotation of the same sequence? Or was the Mantis-annotated sequence compared with a different sequence in Swiss-Pr
had no identifiers in common? If the sequences were the same, then why did they have no identifiers in common? A speci
example would be helpful here.
Response:
As both reviewers pointed out (please see R2C23), the similarity threshold setting was unclear and was therefore remove
entirely from the supplements. In addition, the method used to calculate the similarity between functional descriptions ha
moved to the supplements, as we think it does not entirely fit the context of the manuscript. Please see the section “Cons
between free text functional descriptions” in the supplements.
The threshold benchmark was completely removed; instead, the similarity threshold was determined by empirically evalua
set of 1000 pairwise descriptions. Documents above the threshold of 0.9 (0-1) were considered to be describing the same
function.

R1C7:
How much does the text mining analysis of the free text descriptions contribute to the similarity score? Is the similarity do
by identifier matching or does the free text analysis contribute significantly?
Response:
We analysed the contribution of both methods for the UniProt sample (2010-2020, and found that the free text analysis
contributes roughly 35%.
Since one could argue that Mantis’ performance is simply due to the multiple domains algorithms and consensus generatio
also performed additional tests: (i) Mantis default execution with the DFS algorithm; (ii) Mantis without text analysis with
algorithm; (iii) Mantis without text analysis and identifiers intersection with DFS algorithm; (iv) Mantis default execution w

BPO algorithm; (v) Mantis without text analysis with the BPO algorithm; (vi) Mantis without text analysis and identifiers intersection with BPO algorithm.
Condition iii refers to a protein annotation methodology without consensus generation, but that uses multiple reference da[illegible] and outputs multiple annotations per sequence.
Condition vi refers to the baseline protein annotation methodology with the added benefit of using multiple reference data[illegible]
In essence, each test will check each feature of Mantis individually: inclusion of different domains - DFS/BPO; consensus generation exclusively with IDs intersection; and no consensus generation (without IDs intersection and without text analy[illegible]
We calculated the F1 score of each condition, (i) had a F1 score of 0.8268, (ii) 0.7902, (iii) 0.7740, (iv) 0.8138, (v) 0.778[illegible] (vi) 0.7627. We can see that all conditions had "close" F1 scores indicating that Mantis' superior performance is not cause[illegible] just blindly adding more information. We can also see the additive effect of the algorithm and the consensus generation st[illegible] then also checked these results against eggNOG-mapper where a F1 score of 0.7027 was obtained. Put together, these re[illegible] suggest that simply using multiple reference datasets (condition vi) already boosts the F1 score past eggNOG-mapper; ad[illegible] extra Mantis features (hit processing algorithms and consensus generation) boosts performance even further.
We have added this information to the manuscript in the new subsubsection "Impact of consensus generation" in the subs[illegible] "Initial quality control" in the section "Analysis", page 5, lines 346-368.
Please refer to supplemental table 4 for further details.

R1C8:
Although this information is in the Methods, it would be helpful to define briefly in the Results section how precision is bein[illegible] calculated. It would be useful to include the explanation of why precision is considered more important than recall in the R[illegible] as well.
Response:
While in general we do consider precision more important than recall (since recall tends to be high anyway), we now meas[illegible] performance with the F1 score (harmonic mean of precision and recall). We think this metric will overall be more informat[illegible] suggested, we have also added how each metric is being calculated in the Analysis introduction. Please see the following transcript in page 4, lines 261-275 in the section "Analysis":
In order to compare the performance between the different tests, we calculated a confusion matrix for each test. For futur[illegible] reference, a True-Positive (TP) occurs when a functional annotation (predicted from a PFA tool) shares one or more datab[illegible] with the respective reference annotation (e.g., Pfam ID); a False-Positive (FP) when no database IDs are shared; a False-Negative (FN) when the PFA tool does not annotate a protein sequence but a reference annotation is available; and a True Negative (TN) when the PFA tool does not annotate a protein sequence and no reference annotation is available. Precision[illegible] defined as TP/(TP+FP) , Recall as TP/(TP+FN), and F1 score(harmonic mean of precision and recall) as 2 × (Precision*Recall)/(Precision+Recall). The F1 score is used as a performance metric. Further details on the benchmark are available
in "Establishing a test environment"

R1C9:
It would be useful to move figure 5 earlier in the paper when the hit processing algorithms are first introduced.
Response:
Figure was edited to provide further details on the hit processing algorithms and was moved. It is now figure 2.

R1C10:
In the Background section, annotation iii could be removed from the first two examples of glucose degradation annotation[illegible] because it isn't used to make the authors' point.
Response:
We removed the annotation iii as the reviewer suggested.

Reviewer #2

R2C1:
how several steps of the Mantis annotation algorithm actual work (specifically those related with inter-HMM and consensus[illegible] integration),
Response:
We would like to thank the reviewer for the very in-depth review. We hope we have provided clarity in some of the more [illegible] steps. Please also see R1C1 in relation to this point.

R2C2:
First, the list of keywords maybe should be reviewed: function as a keyword it is too generic in my opinion. protein; functi[illegible]

annotation could be merged into protein function annotation. Also, I could not find an explanation of what NLP means. Ple
review also the other keywords included. The same for the list of abbreviations. For example, I could not find NLP there, b
found that BPO is also not included. Please, review all abbreviations both from the main text and from the list.
Response:
We thank the reviewer for this important point. We have reviewed the keywords and abbreviations as suggested. The key
were changed to "bioinformatics; consensus; homology; hmm; protein function annotation".

R2C3:
Please, review also the text for potential errors. For example, "whilst sing" --> "whilst using", or "herein after" --> "herein
am also not native English, so my review in this sense could be not the best, so maybe a professional language review cou
good to improve the manuscript. I leave this decision to the authors, editor and maybe some Ensligh-native speaker from
the reviewers.
Response:
We would like to thank the reviewer for this comment; indeed, while the authors and a native speaker had previously revi
the paper, some errors escaped our attention. Grammarly was also used for revision. We redoubled our efforts on this end
carefully reviewing the manuscript for typos/errors.

R2C4:
Regarding supplemental material, it has been a bit confusing for me. There are links throughout the text, but there are als
supplemental files at the end of the manuscript. Are all of them valid? Nomenclature of sections, tables, figures, etc from
different materials should be merged, and all supplemental material provided in a common way. This is what I had:
Throughout the text Supplemental Table 1 is a link to
https://htmlpreview.github.io/?https://raw.githubusercontent.com/wiki/PedroMTQ/mantis/Resources/tab_e_value.html
Supplemental Table 2 is a link to
https://htmlpreview.github.io/?https://raw.githubusercontent.com/wiki/PedroMTQ/mantis/Resources/tab_uniprot_algorith
Supplemental Table 3 is a link to
https://htmlpreview.github.io/?https://raw.githubusercontent.com/wiki/PedroMTQ/mantis/Resources/tab_genomes.html

At the end of the manuscript
Supplements.pdf
Defining a similarity threshold (Figure 1)
Annotating metagenomes
0.1 Benchmarking annotation efficiency (Figure 2, Table 1, Table 2)
Supplementary benchmarking against other PFA tools (Table 3, Table 4)
Supplemental tables (Table 5, Table 6, Table 7, Table 8)
References
Supplements.xlsx
Mantis vs emapper
DFS vs BPO vs heuristic
Comparisons with Prokka, DeepEC, emapper, RAST
Response:
Previous links were used as a way to make the manuscript more "interactive", and had the same data found in the supple
Thank you for the suggestion. We have now moved all the tables into a single excel file. All the tables have been numbere
table of contents has been included in the first spreadsheet.

R2C5:
Some comments about the abstract. What do the authors mean with "adaptable"? How is this different from "flexible"? Als
would say that "reproducible" is implicit of computational tools, in general terms. Why are the authors stressing this in the
abstract as a feature specifically of Mantis? Also, the authors say that "Mantis is fast, annotating an average genome in 25
minutes", which I would like better "an average bacterial genome", for instance.
Response:
We used the adjective "flexible" as a means to say that Mantis can be deeply customized, whereas "adaptable" would be i
regards to using different reference HMMs, so that it can be used in niche environments (for example). However, since the
be viewed as synonyms, we standardized nomenclature to only include "flexible". We agree that reproducibility should be
in every bioinformatics software, but unfortunately, it still is not. Therefore, we kept reproducibility as a feature, because
reproducibility remains a relevant topic in bioinformatics. In fact, dedicated tools were created to address this exact issue
Snakemake - https://doi.org/10.1093/bioinformatics/bts480), in addition, a paper from Mangul et al. (2019 -
https://doi.org/10.1371/journal.pbio.3000333) also highlights that reproducibility remains a concern. We removed the exe
time (since it depends on hardware and data at hand), opting only to mention that it is fast due to parallelization.

R2C6

And some comments about the Background section. I personally would remove the first 2 sentences, but this is completel
personal preference. Also, I am not sure to agree with the protein function annotation definition which is given both in the abstract and in Background: "protein function annotation(PFA), which is the identification of regions of interest (domains) sequence and assignment of biological function(s) to these regions."
Response:
We understand there is no universal definition for protein function annotation. From the many available definitions, we giv one that is most appropriate for the task for which we have designed Mantis. Therefore, we now clearly state that this def our understanding of the task and the challenge. We changed the paper accordingly to reflect that this is only how we, the authors, define the problem we aim to address with Mantis.
Please see the following transcript in page 2 lines 21-25 in the section "Background":
One of the ways to make sense of this data is through protein function annotation (PFA), which is, in the context of this pa the identification of regions of interest (i.e., domains) in a sequence and assignment of biological function(s) to these regi

R2C7:
Later, the authors say that: "We reviewed the implementation of three widely used PFA tools [13, 24, 14] and observed th processing of candidate annotations (i.e. sequences or HMM profiles which are highly similar to the query sequence) is dor capturing only the most significant candidate between the references ("best prediction only", herein after called BPO)". Th Prokka, eggNOG-mapper and InterProScan 5. And, "This classic PFA approach works well for single-domain proteins, but r domain proteins may have multiple putative predictions [30, 31, 32], whose location in the sequence may or may not ove

The previous 2 sentences, although are true to some extent, are in my opinion an oversimplification. For example, stating eggNOG-mapper picks the most significant candidate between the references is true, but only part of the story. eggNOG-r picks the most significant homolog as seed to retrieve orthologs from which annotations are transferred. Therefore, a protein can be annotated with multiple domains which may or may not overlap, which I believe i also for Prokka and InterProScan 5. Therefore, although I think that the authors have a valid point here, I believe that the should focus on the advantages of identification of multiple references, but not as a mandatory requirement for multi-dom annotation. Actually, the 3 "reviewed" tools are very different, and if they are mentioned specifically as reviewed, a more description of their approaches, weaknesses or features which could be improved would be expected.
Response:
From what we can tell, Prokka does return the best hit only, as seen in the commands below (from Prokka's GitHub - https://github.com/tseemann/prokka/blob/master/bin/prokka):
Line 69:

```
my $BLASTPCMD = "blastp -query - -db %d -evalue %e -qcov_hsp_perc %c -num_threads 1 -num_descriptions 1 -num_alignments 1 -seg no";
```

In addition, it only returns one hit per sequence as seen in the code below:
Line 1061:

```
for my $db (@database) {
# skip HMMs if --fast mode
if ($fast && $db->{FMT} !~ m/blast/i) {
msg("In --fast mode so skipping non-BLAST search against ".$db->{DB});
next;
}
# create a unqiue output name so we can save them in --debug mode
my $outname = "$prefix.".basename($db->{DB}).".tmp.$$";
# we write out all the CDS which haven't been annotated yet and then search them
my $faa_name = "$outdir/$outname.faa";
open my $faa, '>', $faa_name;

my %cds;
my $count=0;
for my $sid (@seq) {
for my $f (@{ $seq{$sid}{FEATURE} }) {
next unless $f->primary_tag eq 'CDS';
next if $f->has_tag('product');
$cds{++$count} = $f;
print $faa ">$count\n",
$f->seq->translate(-codontable_id=>$gcode, -complete=>1)->seq,"\n";
}
}
close $faa;
```

As the reviewer suggested, eggNOG-mapper is indeed somewhat more complex, and it is relevant not to oversimplify.
Since it is not in the context of the manuscript to extensively review other tools, we changed that particular section of the

manuscript. Please see the following transcript in page 2 lines 50-59 in the section “Background”:
The most common method of processing candidate annotations (i.e., sequences or HMM profiles that are highly similar to query sequence) is done by capturing only the most significant candidate (“best prediction only”, hereinafter called the BP algorithm). This PFA approach works well for single-domain proteins, but multi-domain proteins may have multiple putativ predictions, whose location in the sequence may or may not overlap. This selection criterion may potentially lead to missin annotations and is therefore not suitable in complex PFA scenarios.

R2C8:
Then, regarding consensus integration of annotations the authors say that "This approach addresses three very relevant is with PFA [34, 35, 51, 52]: over-annotation (through the use of overlapping but independent sources, thus obtaining a mo reliable final annotation); under-annotation (through the use of multiple reference sources, which implicitly leads to a wide search space); and elimination of redundancy (through the creation of a consensus-driven annotation)." I agree that this approach would deal with under-annotation and with elimination of redundancy, due to integration of annotations from dif sources. However, I fail to understand how this approach helps with over-annotation.
Response:
We separated redundancy and over-annotation as we think these are different issues. Redundancy is here simply the dupl of data (e.g. having the same identifier multiple times) which can be solved by making sure the annotation metadata (i.e. identifiers and text description) is not repeated. Over-annotation is more complex, and it pertains to the fact that if we us multiple reference datasets, we will most likely end up with annotations pointing to different functions. This is clearly a pro and so this is something we address with the consensus generation; we do so by analysing all the functional annotations f multiple independent reference datasets and checking which ones point towards the same function. If independent referen point towards the same function, we consider it more likely for this function to be the “real” function of the respective prot sequence. This is, as we see it, one of the main benefits of using multiple references.
However, we agree that this was not clear in the initial manuscript, therefore please see the following transcript with the n text in page 3 lines 178-189 in the section “Background”:
Under-annotation is addressed through the use of multiple reference data sources, which implicitly leads to a wider search Over-annotation is minimized through the generation of a consensus-driven annotation, which identifies and merges anno that are consistent (i.e., similar function) with each other (e.g., if three out of five independent sources point towards the function and two others point towards other, unrelated functions, then these three annotations are more likely to be valid) eliminating the remaining inconsistent annotations. Redundancy is eliminated by removing duplicate database IDs and/or extremely similar descriptions.

R2C9:
First, regarding reference data, the authors say that eggNOG OGs are from eggNOG 5. However, actually TSHMMs and annotations downloaded by Mantis correspond to eggNOG 5 data, but the unspecific HMMs are from eggNOG 4.5. Checkin lines of code:

```
./source/MANTIS_DB.py: eggnog_downloads_page_hmm =
'http://eggnogdb.embl.de/download/eggnog_4.5/data/NOG/NOG.hmm.tar.gz'
./source/MANTIS_DB.py: eggnog_downloads_page_annot =
'http://eggnogdb.embl.de/download/eggnog_4.5/data/NOG/NOG.annotations.tsv.gz'
./source/MANTIS_DB.py: eggnog_downloads_page = 'http://eggnog5.embl.de/download/latest/per_tax_level/'+str(taxon
./source/MANTIS_DB.py: url = 'http://eggnogdb.embl.de/download/emapperdb-5.0.0/eggnog.db.gz'
```

This is important, and it will impact both the use of the tool by users and the benchmarks presented in the paper. Note th eggNOG 4.5 and eggNOG 5 orthologous groups are not cross-linked, and therefore annotations for unspecific NOGs will be in Mantis output. For example, I annotated with Mantis a protein using unspecific NOGs as ENOG4111x9y. This is an OG fr eggNOG 4.5, as expected, and I got only the description as annotation ("uncharacterized protein yaho"), when I should be also annotation identifiers, GO terms for example. The equivalent OG in eggNOG 5 is ENOG502DN1T though. This can be comparing OGs from http://eggnog45.embl.de/ and http://eggnog5.embl.de/
Response:
Thank you for this comment, we initially used the data from eggNOG 4.5 as it contained a pre-compiled general HMM. As reviewer mentioned, this will have an impact on the produced annotations, for this reason the general eggNOG HMM is no compilation of all the non-redundant TSHMMs from the following NCBI IDs: 2157 (Archaea), 2 (Bacteria), 2759 (Eukaryota 10239 (Viruses), 28384 (Others), and 12908 (Unclassified). These IDs correspond to NCBI’s top level taxonomy rank IDs. have also added another set of TSHMMs from NCBI.
We have added this information to the manuscript, please see the following transcript in page 8 lines 704-711 in the subs “Methods.Reference data and customization”:

A general NPFM HMM was created by pooling all non-assigned HMM profiles and the TSHMMS from the following NCBI IDs (Archaea), 2 (Bacteria), 2759 (Eukaryota), 10239 (Viruses), 28384 (Others), and 12908 (Unclassified). These IDs corresp NCBI’s top level taxonomy rank IDs. A general eggNOG HMM was created by pooling together the TSHMMs from the

aforementioned taxon IDs.

R2C10:
Besides that, "TSHMMs metadata was extracted from the eggNOG SQL database" I would ask authors to specify that this i
eggNOG-mapper DB, not an eggNOG one, as can be seen in the link above.
Response:
This has now been rectified in the manuscript.

R2C11:
Also, the TSHMMs databases are huge as a whole. In my opinion, including in Mantis an option to download only TSHMMs
specific taxon specific taxa would be great and almost mandatory for the "flexibility"
Response:
Thank you for the suggestion, this feature was implemented and can now be used by providing a list of NCBI IDs or organ
names in the MANTIS.config file (with the line “nog_tax”). If an organism name is introduced, an automatic web search re
the respective NCBI ID. A lineage for each NCBI ID is then generated and all TSHMMs are downloaded and compiled. This
mentioned in the Mantis’ wiki.

R2C12:
i) sample pre-processing, where samples are split in chunks for parallelization. Also, input data should be proteins. Are CD
queries (fasta in nucleotide/DNA format) also accepted?
Response:
No, Mantis doesn’t do gene calling or translation, we opted for this approach to eliminate the need to integrate further thir
bioinformatic tools which often depend on the use-case and/or user preference.

R2C13:
"HMMER outputs a domtblout le [23], where each line corresponds to a hit/match between the reference dataset and the
unknown protein sequence. The e-value within the HMMER command limits the available solution space to be analyzed in
posterior processing steps." Which is the e-value used by Mantis then? The e-value from "the HMMER command" or the e-
from the "domtblout" file? Note that e-value in "domtblout" is the "e-value of the overall sequence/profile comparison (inc
all domains)", which outputs also other values, like "c-evalue" and "i-evalue".
(http://eddylab.org/software/hmmer/Userguide.pdf, pp70-71, see also pp 34-35). Therefore, if using the "e-value", either
hits from such domain should be included for intra-HMM integration, or preferably some hits should be discarded using the
evalue", for example. Also note that as the score used to compare combinations through DFS includes e-value, if several r
domains exist in a sequence maybe the i-evalue should be included in the score instead of the overall e-value several time
which would inflate the combination in which several domains are part of the sequence.
Response:
Regarding the sentence:
Which is the e-value used by Mantis then? The e-value from "the HMMER command" or the e-value from the "domtblout"
We are not sure what the reviewer means here, the e-value from the HMMER command is to set the threshold, the actual
that is extracted (and used by Mantis) is the one that is reported in the domtblout file.
As the reviewer noted the DFS and heuristic algorithms should use the independent e-value - column 12 (which they did a
whereas the BPO should use the full sequence e-value - column 6 (it didn’t before). The full sequence e-value is used by t
algorithm since we are interested in only the best prediction for a certain protein sequence.
We have also now specified in HMMER’s execution to use the “--domE” threshold when using the DFS and BPO algorithm,
the “-E” threshold when using the BPO algorithm.
We have now specified this in the paper, please see the following transcript from page 9, lines 758-763 in the subsection
hits per protein” in the section “Methods”:
Mantis uses HMMER's independent e-value when using the DFS and heuristic algorithms; whereas it uses the full sequence
value when using the BPO algorithm (since only the best hit is extracted per protein sequence). For simplicity purposes, b
simply referred to as e-value throughout this paper.
And from the supplements section “Execution commands”:
When using the DFS or heuristic algorithm, Mantis uses HMMER's independent e-value as a confidence score, and, when u
BPO algorithm, it uses the full sequence e-value (see http://eddylab.org/software/hmmer/Userguide.pdf pp.71-72). The e
threshold set in the hmmsearch will be the same for all algorithms, however, when running Mantis with the DFS or heurist
threshold variable will be -domE, whereas for BPO, it will be -E. The e-value type and threshold chosen reflect the fact tha
DFS and heuristic algorithm may capture multiple hits per protein, whereas the BPO only captures the one hit per sequenc

R2C14:
"should the DFS algorithm running time exceed 60 seconds, Mantis employs the previously described "heuristic" algorithm
which scales linearly and outputs an unique combination of hits." How many queries in average could be affected by this?
user warned somehow if some results are produced with the heuristic algorithm due to this DFS time limit?
Response:

Yes, the user is warned if the DFS algorithm exceeds the time limit, this is now specified in the manuscript. The user can a
customize the time limit and/or choose which algorithms to use (BPO, heuristic or DFS). We calculated how many times th
heuristic algorithm was used as a backup during the hit processing of the Swiss-Prot sample (2010-2020). We found that
intra-HMMs hit processing, the heuristic algorithm was used in roughly 7.2% of the sequences, and for the inter-HMMs hit
processing in 0.5% of the sequences. This information has been added to the manuscript in the section “Hit processing
approximation”.

R2C15:
Are "output_annotation.tsv" files a product of this 3rd step?
iv) metadata integration. Annotation description and identifiers are added to the respective hits. I guess this yields the
"integrated_annotation.tsv" output files.
Response:
The "output_annotation.tsv" is a byproduct of the intra-HMM hit processing step, which is specific to each reference. Howe
have all predictions in one file, we merge all hits from the different references into one file. The "integrated_annotation.tsv
fundamentally the "output_annotation.tsv" but with metadata. The "consensus_annotation.tsv" contains, for each referenc
consensus annotation from the "integrated_annotation.tsv", meaning that if, for example, a protein sequence has five hits
"integrated_annotation.tsv" file and three of those are consistent (either through identifiers or functional text description)
only those three are included in the "consensus_annotation.tsv", the remaining twp are rejected. Of course, the user may
interested in the rejected hits, which is why we opted to also provide the "integrated_annotation.tsv" file.
We have now specified in which step each output file is generated. Please see the following transcript in page 8 lines 675-
the subsection “Methods.Input and output”:
Mantis outputs, for each sample, three tab-separated files, each corresponding to a different step in Mantis' workflow: (i)
output output_annotation.tsv, with all the hits, their e-value, and coordinates; (ii) integrated_annotation.tsv, with the san
information as output_annotation.tsv, but also with hits metadata (e.g., KEGG orthology IDs (KO), enzyme commission (E
numbers, free-text functional description, etc); (iii) the main output file consensus_annotation.tsv, with each query protei
and their respective consensus annotation from the different reference data sources (e.g., Pfam). These files provide
contextualized output in a format that is both human and machine-readable.
A Mantis.out file is also provided per sample, serving as a log file for each execution step.

R2C16:
v) inter-HMM reference hits processing.
I have many doubts with this step. First, in "Mantis" section it is said that "During inter-HMM hits processing the DFS algo
again used to generate all the combinations of hits for all HMM sources." However, in "Methods - Using multiple reference
datasets" this is not confirmed not further explained.
Response:
We added a new figure (7) for inter-HMMs hit processing in the section “Using multiple reference datasets”, where we exp
each step and give specific examples. Succinctly, here we explain how the DFS algorithm is used to generate the combina
hits (similar to the inter-HMM hits processing but instead of generating reference-specific combinations we pool all hits an
generate combinations from that merged pool). We have extensively changed the “Multiple predictions per protein” subse
please refer to that section. We hope the explanation is now clear.

R2C17:
Also, if DFS is used here, how are the e-values from the different references used to compute the scores. Because e-value
depend on database size, in contrast with bit-scores.
Response:
Thank you for the remark regarding e-value, however we already took this into account when implementing Mantis. Quoti
33 of HMMER’s user guide (http://eddylab.org/software/hmmer/Userguide.pdf):
“The E-value is based on the sequence bit score, the second number.This is the log-odds score for the complete sequence
people like to see a bit score instead of an E-value, because the bit score doesn’t depend on the size of the sequence data
only on the profile and the target sequence. The E-value does depend on the size of the database you search: if you searc
database ten times larger,you get ten times the number of false positives”
And again quoting the same user guide, page 70:
“Well, first of all,they still wouldn’t be identical, because the target database size used for E-value calculations is different
(number of target sequences for hmmsearch, number of target profiles for hmmscan, and it’s good not to forget this.”
As implied by the quotes, when using hmmsearch, the reference database size does not matter, the e-value is only scaled
number of sequences in the target sample (e.g. the same sequence in a sample of 1 sequence will have double the e-valu
was instead present in a sample of 2 sequences, however the same sequence searched against a database with 10 or 500
will have the same e-value). Hmmscan is scaled to database size and so the e-value will now be independent of sample si
dependent on database size.
Since, during homologs search, Mantis uses hmmsearch and the same sample for different references, then the e-value ca
safely used. There are two exceptions to this: (i) when Mantis splits the sample into chunks, and (ii) when using TSHMMs
each taxon we only annotate the protein sequences which have not been annotated in the previous taxons). In both cases

values are adjusted to the original sample size by storing the original file sample size in a separate intermediate file.

R2C18:
Also, query coverage will be very different by the very nature of the HMM profiles of the different databases. For example, [illegible] is a database of domains, whereas eggNOG contains profiles which usually span a large section of the protein sequence.
Response:
This is true, which is why we use a composite combination score. Query coverage is taken into account, yes but so is the [illegible] In this manner (and since we scaled the e-value), small but highly significant hits are also taken into account when other [illegible] large but with poor significance. This is, of course, a compromise, but it does permit for a fairer use of both larger (i.e. eg[illegible] and smaller (i.e. Pfam) hmm profiles.
Also to note that since Pfam is made up of domains, if, for example, a protein sequence has 2 Pfam hits and 1 eggNOG hi[illegible] is likely that these 2 Pfam hits will be contained within the eggNOG hit. During consensus generation, should these share [illegible] identifiers or have similar functional descriptions, the Pfam hits would also be included (this is actually one of the benefits [illegible] consensus-driven annotation). While we do agree that this is not a perfect solution, to our knowledge, it is one of the mos[illegible] approaches when dynamically integrating references that are heterogeneous in composition and scale.

R2C19:
Moreover, it said later that "Since several groups of consensus annotations may be generated, we evaluate their quality a[illegible] select the best one, considering the following: percentage of the sequence covered by the hits in the consensus, the signi[illegible] of the hits (e-value) in the consensus, significance of the reference datasets (customizable), and the number of different reference datasets in the consensus."
"Since some sources are more specific than others, the user may also customize the weight given to each source during consensus generation [73]." (Methods - Reference data and customization)
Does this have to do with the DFS algorithm for inter-HMM integration? Also, how is the weighting of different references [illegible] the GitHub it is explained that it is a [0-1] value, but, is it just a priority value or is it included in the DFS scoring formula somehow? I fail to find an explanation about how this weighting is used at all.
Response:
The scoring variables for inter and intra-HMM hit processing are now fully explained in the manuscript, which includes the [illegible] values of each reference. The default weight values were defined according to the level of curation and specificity of the re[illegible] sources but can be changed by the user. These weights are only used during inter-HMMs hit processing since it is only the[illegible] we compare different reference sources. As the reviewer mentioned, yes it is only used during the DFS scoring formula (si[illegible] other algorithms will only generate one solution with one - BPO or multiple hits - heuristic).
Please see the following transcript in page 10 lines 892-904 in the subsection “Using multiple reference data sources” in t[illegible] section “Methods” for the changes in the inter-HMMs hit processing:
HMM reference weight (HMMW) - average weight of all the reference data sources within the combination. This is calculat[illegible] adding all hits' hmm weights and dividing this sum by the number of hits in the combination (e.g., if a hit comes from Pfa[illegible] has a weight of 1, and another from eggNOG, that has a weight of 0.8, HMMW would equal to (0.9+0.8)/{2 = 0.85). The [illegible] weight for each default reference data source has been set according to the authors' perception of the reference quality - [illegible] method, curation level, and annotation completeness (eggNOG - 0.8, Pfam - 0.9, NPFM - 0.7, and TIGRfam - 0.5). This w[illegible] customizable, the default weight for custom reference data is 0.7 (which can also be customized).

R2C20:
vi) Consensus generation
From Background section: "However, to our knowledge, there is no tool for the dynamic generation of a consensus from [illegible] protein annotations." "We implemented a two-fold approach to build a consensus annotation, first by checking for any int[illegible] annotation identifiers and second by evaluating how similar the free-text annotation descriptions are." From the "Mantis" [illegible] "all the combinations of hits are expanded and intersected (if possible), the best consensus combination of hits is then sel[illegible] for each query sequence."

So the tool is "checking" and/or "evaluating" intersections of identifiers and similarity of descriptions. But I fail to underst[illegible] is the desired or expected output after the check is done. According to the text, one would expect to obtain the intersecti[illegible] identifiers, which actually does not make sense at all, since many identifiers would be discarded just because they are not [illegible] one or the other reference. The same question regarding descriptions: several descriptions are compared in a pairwise ma[illegible] and those pairs with similarity are kept and those without any similar description are discarded?
Response:
We have rephrased that specific part of the section Mantis, as it was somewhat confusing (we erroneously used the word “intersected”). Please see the following transcript in page 3 lines 227-232 in the section “Mantis”:
Finally, consensus generation ensures the best combination of hits among all hits from the multiple reference data source[illegible] selected. This combination is expanded by adding additional hits with consistent metadata (intersecting identifiers or simil[illegible] functional descriptions).
To answer the reviewer questions, we aim at finding consistent information across multiple references, doing so by checki[illegible] shared identifiers or similar functional descriptions. We hypothesize that finding consistent information between independ[illegible]

generated sources will implicitly lead to a functional annotation that is more likely the ground-truth.
While some information is lost due to the incapacity to find consistent data across different references (either through iden
or descriptions), our data also suggests that extensive cross-linking is already available, as many databases already provi
cross-linking between multiple databases (e.g. KEGG, eggNOG, Uniprot, etc). The consensus thus leverages this feature o
databases. This cross-linking has not been leveraged only by us, but many other projects (e.g.
https://doi.org/10.1093/nar/gkv1117).

R2C21:
Again, in "Methods - using multiple reference datasets": "For the integration of multiple reference datasets, a two-fold tex
approach was used: 1. Consensus between identifiers; and 2. Consensus between the free-text annotation description." "
consensus between identifiers is calculated by identifying intersections between the different sources. Identifiers within th
text annotation descriptions are extracted and used here."

And also, "If no consensus between identifiers is found, then we proceed with a consensus calculation between annotation
descriptions." so I guess that similarity of descriptions is only evaluated when no intersection is found among all the anno
identifiers? If this is true, then multiple identical descriptions would be kept just because a single domain is common to bo
reference sources? My sincere apologies, but I am missing some key point for sure here. I would ask the authors to explai
more detail how this works.
Response:
The intersection of identifiers is a fast and reliable approach, however, when no intersection is found, we use the descripti
similarity to find consistent hits, which is slower and less reliable. We chose to use a simple intersection of IDs (and not
something like the Jaccard index) because some databases provide minimal cross-linking; in addition, if two functional
annotations for the same protein sequence share at least one ID, then it is likely they are functionally similar or at least re
(assuming independent references naturally converge to the same function).
In terms of the metadata (identifiers and text descriptions) that is kept, if two hits share an identifier (e.g. hit 1 "glucose
degradation; ID1, ID2" and hit 2 "glucose degradation; ID1, ID3") then the resulting consensus annotation would be the
combination of non-repeated metadata (e.g. "glucose degradation; ID1, ID2, ID3"). Similar functional descriptions, unless
the same, are kept regardless of their text similarity (e.g. "glucose degradation" and "degrades glucose"). We chose to do
two reasons: (i) eliminate the need to assess the similarity score for all functional descriptions (higher runtime), and (ii) a
removing functional descriptions erroneously identified as highly similar (which of course still happens since similarity ana
most statistical tools, is also prone to false positives).

R2C22:
Also, if I understood correctly, there can be several descriptions from a single source. For example, if the right combinatio
for a query are 2 different PFAM domains, with different descriptions. How is this handled? Are all descriptions compared i
pairwise manner intra and inter reference?
Response:
If two hits are from the same reference source, these are not compared. In addition, two hits need to have a coordinate o
of at least 70%, meaning that if a hit from Pfam is located in 3-10 and eggNOG in 8-20 and they have the same function,
their residues are not overlapping sufficiently, they are not merged in the consensus annotation. If another eggNOG hit (w
same function) now has coordinates in 5-12, then this hit and the Pfam hit are merged (since there is sufficient residues o
Please see the section "Using multiple reference datasets" for an explanation on this.

R2C23:
Regarding the similarity of descriptions, the authors say that "Alongside Mantis, We developed a standalone tool [58] that
the use of multiple reference datasets through the generation of a consensus annotation. As we have shown in the supple
"Defining a similarity threshold", this tool has high specificity, thus, in the context of Mantis, it allows for the correct ident
of similar free-text annotation descriptions."

I guess this "high specificity" and how the threshold is defined should be part of the main manuscript in the Methods secti
Even if this is left in the supplemental, it should be explained how this "high specificity" is achieved, and also how the defi
0.8 as threshold is not arbitrary, and it is not as good as 0.6, 0.7 or 0.9. Also they say (in supplemental) that: "In the cur
scenario, sensitivity is unimportant for two reasons:
1. the reference datasets from Mantis may contain data from Swiss-Prot (TPs inflation)
2. two annotations may completely agree in identifiers but, while describing the same function, be lexically different (FNs
inflation)"
which I fail to understand. They define "False-positives (FP) = similarity score is above the threshold and identifiers disag
which they say in the introduction that it is the goal of the consensus-description annotation. Thus, if you define these as
are contradicting the goal you define in the introduction. Also, "False-negatives (FN) = similarity score is below the thresh
identifiers agree", even when FNs are not going to be processed if those annotations with common identifiers are not proc
for consensus-description, it genuinely gives an idea of the FN rate you get, and thus the sensitivity of the method, which
relevant to assess the potential impact of the consensus-description method to expand the annotations from results witho

common identifiers.
Response:
Thank you for this comment. We expanded on this topic, please refer to R1C6.

R2C24:
Besides the above comments, I tried to understand how these v and vi steps work by looking at some examples.
Example 1: 5 descriptions from a query:
description:Response Regulator
description:Two-component systems (sub1role)
description:Signal transduction (mainrole)
description:Transcriptional regulator
description:two-component system, sensor histidine kinase ChiS
Shouldn't at least 2 of the descriptions be merged?
Response:
We assume the reviewer means the descriptions “Two-component systems (sub1role)” and “two-component system, sens
histidine kinase ChiS”, which share the sentence “two-component systems”. Despite possibly being redundant these two descriptions are quite dissimilar, through manual curation it is easy to identify them as redundant, however doing so automatically may also eliminate important descriptions. Please also see R2C21.

R2C25:
Example 2: 7 descriptions from a query
description:DNA metabolism (mainrole)
description:helicase
description:Restriction endonuclease
description:Type ISP C-terminal specificity domain
description:Restriction/modification (sub1role)
description:type iii restriction protein res subunit
description:Type III restriction enzyme, res subunit

Example 3: full annotation of a query
query_ID NOGG_merged;Pfam-A Plug;ENOG410XQM1 2 6 | pfam:PF07715
description:tonB-dependent Receptor Plug
description:TonB-dependent Receptor Plug Domain
Response:
We assume the reviewer means the last two descriptions of both examples, in this case, we agree the descriptions are red
We have now implemented a description elimination redundancy method to check for these very small discrepancies. Plea
the following transcript in page 10 lines 923-925 in the subsection “Methods.Using multiple reference data sources”:
Redundant (e.g., repeated identifiers or functional descriptions) or poor quality information (e.g. "hypothetical protein") is
removed from the consensus annotation.
Since it would be too many to list we do not enumerate all the possible “poor quality information” in the manuscript. Pleas
the following code transcript regarding the redundancy elimination:

```
def remove_redundant_descriptions(self, all_descriptions):
res = set()
already_added = set()
unspecific_tokens=[‘enzyme’,’protein’,domain]
for d in all_descriptions:
test = d.lower()
for p in set(punctuation):
test = test.replace(p, '')
test = test.replace('\'', '')
test = test.replace('\"', '')
test = test.strip()
test = test.split(' ')
test = [i.strip() for i in test if i not in unspecific_tokens]
test = ' '.join(test)

if test not in already_added:
res.add(d)
already_added.add(test)
return res
```

Please see the following code transcript regarding the “poor quality information”:

```
def remove_trash_descriptions(self, all_descriptions):
```

```
res = set()
for d in all_descriptions:
current_d = d.strip().lower()
if d.strip().lower() not in [
'enzyme',
'domain',
'protein',
'unknown function',
'domain of unknown function',
'protein of unknown function',
'uncharacterised protein family',
'unknown protein family',
'uncharacterized protein',
'uncharacterised protein',
'uncharacterised conserved protein',
'uncharacterized conserved protein',
'hypothetical protein',
]:
if re.search('(protein|domain|domian|family|repeat|short repeats|region) (of|with) (unknown|unknwon)
function(\s\(?[dp]uf\d{2,}\)?)?',
current_d):
pass
else:
res.add(d)
return res
```

R2C26:
Example 4: comparing hits with and without tax info (-od)
without -od
NP_416050.4 kofam_merged K15268 1 4 | cog:COG0697 kegg_ko:K15268 tcdb:2.A.7.3.2 description:O\-acetylserine/cys
efflux transporter
with -od "Escherichia coli" NP_416050.4 kofam_merged;NOGT561_merged K15268;3XP05 2 4 |
bigg_reaction:iECIAI39_1322.ECIAI39_1\835 cog:COG0697 go:0000101 go:0003333 go:0003674 go:0005215 go:00055
go:0005623 go\:0005886 go:0006810 go:0006811 go:0006812 go:0006820 go:0006865 go:0008150 go:0015562 \ go:0
go:0015804 go:0015849 go:0016020 go:0016021 go:0022857 go:0031224 go:0032973 \ go:0033228 go:0034220 go:0
go:0044425 go:0044464 go:0046942 go:0051179 go:005123\4 go:0055085 go:0071702 go:0071705 go:0071944 go:00
go:0098655 go:0098656 go:014\0115 go:1903712 go:1903825 go:1905039 kegg_brite:ko00000
kegg_brite:ko02000 kegg_ko:K15268 tcd\b:2.A.7.3.2 description:O-acetylserine/cysteine efflux transporter description:M
an export pump for cysteine and other\ metabolites of the cysteine pathway (such as N-acetyl-L-serine (S) and O- acetyl-
(OAS)), and for other amino acids \and their metabolites

In this case, the annotation with -od seems richer. Besides that, there is certain redundancy in identifiers (K15268) and p
in descriptions also (cysteine transporter, export for cysteine).
Response:
Thank you for this comment, however please note that the quality of the annotations (regardless of using or omitting -od)
entirely dependent on the quality of the reference data sources. Should an organism contain protein sequences that have
well characterized in the reference, then inherently, the annotation will also tend to be richer. Mantis does not create any
reference data de novo,it merely downloads and structures publicly available reference data.
Please note that the K15268 is not repeated, the first K15268 is in the HMM profile names columns, whereas the second K
("kegg_ko:K15268") is in the annotation data. For easier parsing, hit information comes before the "|", and annotation
information after the "|".
Lastly, regarding the description redundancy "cysteine transporter" and "export for cysteine", this issue was discussed in I
Unfortunately, without the creation of a scalable biological lexicon, this issue is unavoidable. Creating a biological lexicon i
within the current scope of Mantis.

R2C27:
Example 5: comparing hits with and without tax info (-od) (II)
without -od
NP_417950.1 kofam_merged;NOGG_merged COG0306;K16322 2 3 | enzyme_ec:3.4.14.10 enzyme_ec:5.4\
.2.12 bigg_reaction:iECABU_c1320.ECABU_c33930 bigg_reaction:iECO111_1330.ECO111_4301 bigg_reaction:iNJ661.Rv0
gg_reaction:iNJ661.Rv2281 bigg_reaction:iPC815.YPO3967 cog:COG0306
… GO terms … KEGG brite terms … kegg_ko:K01280 kegg_ko:K03306 kegg_ko:K03569 kegg_ko:K04043 kegg_ko:K146

kegg_ko:K15633 kegg_ko:K16322 kegg_ko:K16331 … KEGG modules, pathways, ...tcdb:2.A.20.1 description:inorganic phosphate transmembrane transporter activity \ description:low-affinity inorganic phosphate transporter
with -od "Escherichia coli"
NP_417950.1 kofam_merged;NOGT561_merged K16322;3XMW3 2 3 | bigg_reaction:iECO111_1330.ECO111_430\1 cog:COG0306 … GO terms ... kegg_brite:ko00000 \ kegg_brite:ko02000 kegg_ko:K16322 tcdb:2.A.20.1 description:low-affinity inorganic phosphate transporter
description:Low-affinity inorganic phosphate transport. Can also transport arsenate
In this case, the without -od annotation seems richier. Again, KEGG and descriptions consensus seem to be somewhat lim[illegible]
Response:
Please see R2C26. Additionally, we also found similar results with eggNOG-mapper, where, for some organisms, annotatio[illegible] quality was higher without using tax_scope.

R2C28:
Example 6: inter-HMM integration
from "output_annotation.tsv"
YP_025301.1 Pfam-A HOK_GEF PF01848.17 5.93440414507772e-22 49 8 43 2 42
YP_025301.1 kofam_merged K18921 - 1.3407357512953368e-23
49 4 46 42 89
YP_025301.1 NOGG_merged NOG.ENOG410ZT31.meta_raw - 2.1979274611398962e-27
49 4 46 56 102
Here all the 3 hits overlap.
From "integrated_annotation.tsv"
Query HMM_file HMM_hit HMM_hit_ac evalue l qs qe ss se
YP_025301.1 Pfam-A HOK_GEF PF01848.17 5.93440414507772e-22 49 8 43 2 42 | pfam:PF01848 \ description:Hok/gef [illegible]
YP_025301.1 kofam_merged K18921 - 1.3407357512953368e-23 49 4 46 42 89 | kegg_ko:K18921 \ description:protein [illegible]
YP_025301.1 NOGG_merged ENOG410ZT31 - 2.1979274611398962e-27 49 4 46 56 102 | description:Hok/gef family
The final "consensus_annotation.tsv"
YP_025301.1 Pfam-A;NOGG_merged ENOG410ZT31;HOK_GEF 2 3 | pfam:PF01848 description:Hok/gef f\amily

Why is the Pfam included here if it does overlap with the NOGG result? Why not the kofam hit? Descriptions of PFAM and [illegible] match, and the evalue is better for NOGG. I would expect either PFAM or NOGG to be discarded, and, just maybe, kofam [illegible] due to slightly different description?
Response:
The idea of the consensus generation is to attempt to find different references that point towards the same function, thus,[illegible] the hits from Pfam-A and NOGG_merged point towards the same function and overlap in residues, they are kept as the fi[illegible] annotation. The hit from kofam_merged does not seem consistent (at least in terms of identifiers and similarity analysis) [illegible] other hits, in addition, this hit's coordinates also overlap with the other hits, therefore, for these two reasons, it's rejected[illegible] Residues overlap as a requirement for hit consistency was recently added, please see the following transcript in page 10, [illegible] 911-913 in the subsection "Methods.Using multiple reference data sources":
Note that hit metadata consistency (through IDs or descriptions) requires a minimum of 70% residues overlap (default bu[illegible] changed).

R2C29:
Example 7: the same as above, but with -od "Escherichia coli"
From "integrated_annotation.tsv":
YP_025301.1 Pfam-A HOK_GEF PF01848.17 5.93440414507772e-22 49 8 43 2 42 | pfam:PF01848 d\escription:Hok/gef f[illegible]
YP_025301.1 kofam_merged K18921 - 1.3407357512953368e-23 49 4 46 42 89 | kegg_ko:K18921 d\escription:protein [illegible]
YP_025301.1 NOGT561_merged 3XRAS - 1.4945906735751294e-15 49 6 46 7 49 | description:Hok/gef family
Final "consensus_annotation.tsv"
YP_025301.1 kofam_merged K18921 1 3 | kegg_ko:K18921 description:protein HokB

In this case only the kofam hit is kept (due to being the one with the best evalue?).
Response:
The KOfam hit does not crosslink with the other reference data sources. While Pfam and NOGG have the same description[illegible] hits have a higher e-value and cover a smaller percentage of the protein sequence. This behaviour depends on the scorin[illegible] formula, but is working as intended.

R2C30:
Example 8:
From "output_annotations.tsv"
1000565.METUNv1_03812 tigrfam_merged TIGR00092 TIGR00092 1.1e-161 363 20 \344 1 368
1000565.METUNv1_03812 kofam_merged K19788 - 2.7e-151 363 20 \344 79 446

1000565.METUNv1_03812 Pfam-A MMR_HSR1 PF01926.24 3.5e-26 363 13 \158 2 87
1000565.METUNv1_03812 Pfam-A YchF-GTPase_C PF06071.14 3.7e-42 363 284 \357 1 84
1000565.METUNv1_03812 NOGG_merged NOG.C.meta_raw - 4.7e-144 363 20 \344 273 573
From "consensus_annotation.tsv"
1000565.METUNv1_03812 kofam_merged;tigrfam_merged;NOGG_merged COG0012;TIGR00092;K19788
The PFAM domains are discarded, whereas kofam and NOGG results are included despite overlapping with with TIGRFAM,
has the best e-value.
Response:
The reason PFAM was discarded was due to the Mantis's consensus to prefer annotations where sources are consistent wit
other. Unlike Pfam, all other references are interconnected:
Tigrfam metadata: GTP-binding protein YchF; GO:0005525
eggNOG metadata: K19788, GO:0005525
KOfam metadata: K19788 , COG:COG0012
This example also highlights the potential of cross-linking between reference data sources, an essential feature for the ge
of a consensus annotation.

R2C31:
Example 9:

From "output_annotation.tsv"

362663.ECP_0061 kofam_merged K02336 - 0.0 783 41 \743 96 876
362663.ECP_0061 Pfam-A DNA_pol_B PF00136.22 5e-47 783 385 \744 22 414
362663.ECP_0061 NOGG_merged NOG.COG0417.clustalo_raw - 2.4e-127 783 70 \742 872 1449
362663.ECP_0061 Pfam-A DNA_pol_B_exo1 PF03104.20 4.6e-10 783 197 \290 241 337
362663.ECP_0061 tigrfam_merged TIGR00592 TIGR00592 8.4e-49 783 217 \736 597 1141

From "consensus_annotation.tsv"

362663.ECP_0061 kofam_merged;NOGG_merged K02336;COG0417

In this case TIGRFAM and PFAM are discarded, whereas NOGG is kept despite overlapping with kofam hit and having wors
value.
Response:
The hits K02336 (the hit with the best e-value) and COG0417 share the kegg identifier K02336, which is why they are me

R2C32:
Overall, I have problems understanding steps v and vi: inter-HMM integration and consensus generation. In my opinion, t
explanations about how the algorithm works should be improved, made more accurate and exhaustive, at least in the Met
section. It could be needed to adjust some of the procedures/parameters to obtain more comprehensive and less redunda
annotations, which should be one of the major advantages of Mantis according to the authors.
Response:
Thank you for the exhaustive review of the algorithms, it really highlighted the need to provide more and better examples
have prepared new figures and examples for the manuscript. We have also explained the reasoning behind some of the gi
examples, we hope it clarifies steps taken and removes all doubts. Please also see R1C1.

R2C33:
I think that if authors want to state that Mantis is scalable to metagenomes they should provide data supporting this in th
text. According to the results in the supplements, it is indeed scalable, in my opinion, although I ran a test with a sample
100k queries and got rather slower results, but capable of running the analysis in a decent amount of time (120946 secs
hours) anyway.
Response:
There are several parameters to make a tool scalable, one of the most important is workflow efficiency. Mantis execution
directly scales with the number of protein sequences, which is, of course, due to the fact that with more protein sequence
searches need to be performed against the reference data. While an iterative execution approach scales linearly, we paral
the steps in Mantis, thus tremendously reducing execution time.
Since parallel work needs to be distributed across different physical processors, hardware will also play a very important r
These include number of processors, processor speed, available RAM, read/write disk speed, etc.
When we state that Mantis is scalable, we say so for two main reasons - we parallelized the execution and we made sure
RAM usage is kept as low as possible. In this manner, Mantis can be used in a personal computer or on an HPC platform,
execution time will depend on the amount of sequences and the available hardware (which Mantis will automatically detec
As such, scaling to metagenomes does not mean much, as it is only the annotation of a larger amount of protein sequenc

Regardless, we also annotated different environmental samples to prove Mantis has no issues scaling to metagenomes. Th
now been moved from the supplements to the main manuscript as section “Annotating metagenomes”.

R2C34:
Methods - Establishing a test environment "True-Positives (TP) are evaluated via two main methods: i) identifiers match a
description match"

Each query can have multiple annotation items (identifiers and descriptions). Each item that matches with one of the refer
a new TP? Or a TP is the annotation of query as a whole? This should further explained.

The same for FP "Annotations which do not match with the reference annotation are classied as False-Positives (FP)."

Is it the whole annotation or each of the items (identifiers and descriptions) could be a FP or TP independently of the othe
Response:
All the classes in the confusion matrix correspond to an evaluation of the whole sequence annotation. Please see R1C2.

R2C35:
How are FPs, TPs ... computed when benchmarking SwissProt proteins? What are the annotation identifiers used from Swi
Or is it only description used?
Response:
The previous benchmark included, besides IDs, an analysis of the text-similarity between functional descriptions. We char
benchmark to only take into account identifiers (enzyme ECs, Gene ontology (GO) IDs, eggNOG IDs, KEGG orthology (KO
Pfam IDs, and TIGRfam IDs). The confusion matrix construction is the same across all benchmarks done in the paper. We
more thorough description of this process. Please also see R1C4.

R2C36:
"Whenever Mantis outputs a valid annotation but the reference is either of poor quality or non-existent, then this annotati
considered a potentially new annotation." (Methods - Establishing a test environment)

I think this sentence is a bit obscure. What is a "valid annotation" from Mantis, in comparison with a reference which is of
quality or non-existent", and what does mean for FPs and TPs the consideration of "potentially new annotation"? Is it not
considered as a TP, a FP, neither? Shouldn't references of poor quality or non-existent just be removed from the benchma
Response:
We opted not to remove these poor-quality/non-existent reference annotations from the benchmark, but instead to mark
PNA (if the tool annotates the respective sequence). We think this is important since it can hint at a tool’s capacity to disc
novel functions. While we consider UniProt the gold-standard during the benchmark, we know this is not exactly true; whi
UniProt does provide very high-quality annotations, it also contains plenty of poor-quality and automatically generated
annotations. Please also see R1C5.

R2C37:
"Annotation coverage is defined here as the number of Mantis' annotations divided by the total amount of protein sequenc
sample." (Methods - Establishing a test environment)

Is this the same as the % of queries which have received (consensus) annotation?
Response:
This is not only valid for Mantis, but also the other tools. In the case of Mantis, yes, it is the same as the amount of
queries/sequences that received the consensus annotation, out of all the sample sequences.

R2C38:
"As seen in supplemental Table 1, being more permissive (by using a higher e-value threshold e.g. 1e - 3) resulted in a hi
annotation coverage and a higher precision." (Analysis - Initial quality control - Function assignment e-value threshold)

It is surprising that higher e-value threshold leads to higher precision: "This is due to Mantis' internal quality control in the
the DFS hit-processing algorithm. Being too strict with the e-value threshold constricts the available solution space, result
lower precision." However, expanding the solution space would yield higher TP rate, which potentially could lead to higher
precision if FP rate does not increase so much as TP rate. However, in supplements.xlsx it is shown (e-value tab) that abs
number of FPs are reduced when using a higher e-value, and this is much more surprising. Although this could be true, th
authors should check this result is an artifact of the definitions and methods used to compute TP/FPs should be discarded.
authors really did, my apologies, and also maybe they could further explain why this happens.
Response:
Thank you for pointing this out. We redid the benchmark and found similar results. As an example, please imagine two sc

in the first we run Mantis with an e-value threshold of 1e-3 and obtain nine hits (five from the same database and four fro
different databases); in the second we run Mantis with an e-value threshold of 1e-30 and obtain five hits (all from the sar
database). In the first scenario, out of all these hits, during hit-processing, we find that four hits from different databases
towards the same function, however, these don’t necessarily appear to be the most significant (e.g. four with e-values abo
30 and another with e-value 1e-35). In the second scenario, from the previous hits, only the hit with e-value 1e-35 is pres
(since all others are above the e-value threshold). In the first scenario, during hit processing Mantis decides to pick these
functionally consistent hits since they come from five different databases, whereas in the second scenario another hit is ch
(e.g. a hit with e-value 1e-36).
In the first scenario the final annotation will contain the unified functional annotation of the five hits, which incidentally wi
contain identifiers that match with the reference annotation and this is then considered a TP. In the second scenario the h
chosen does not contain any identifiers that match with the reference annotation and so it is marked as a FP.
This is of course a limitation of the benchmark (since we are limited to evaluating IDs) but more importantly also due to th
that a lower threshold severely limits amount of available information and thus the integration of data from different datal
The e-value threshold will also change the combination score during hit processing. We have added this discussion (with a
example) to the supplemental PDF for readers that might question the same. While we agree that these results are counte
intuitive (and hard to explain due to the amount of different parts in the workflow), we hope we provided a simple examp
explains why this may occur.

R2C39:
Initial Quality Control "When comparing the hit processing algorithms we found that the DFS algorithm consistently outper
the other algorithms, with an average precision 0.038 and 0.013 higher than the BPO and heuristic algorithms respectivel
Is this difference statistically significative?
Response:
Due to the “low” amount of samples benchmarked we did not find it appropriate to test the significance of the differences
the algorithms (due to low power). We now include a new section “Impact of hit processing algorithms” where we create s
samples and test the impact of the algorithms on F1 score.

R2C40:
"for homology search, Mantis uses HMMER [23] and eggNOG-mapper uses Diamond [22]." (Methods - Establishing a test
environment)
This is not entirely true, since eggNOG-mapper can use both HMMER and/or Diamond, depending on the eggNOG-mapper
being used. I would rather prefer "and for eggNOG-mapper we used the Diamond-based search".
Response:
Thank you for this remark, we initially checked with one of eggNOG-mapper‘s developers (coincidently, the reviewer) rega
this, and we found, at the time, that the current eggNOG-mapper v2 could not use HMMER, only Diamond. Please see the
here (https://github.com/eggnogdb/eggnog-mapper/issues/219) and here (https://github.com/eggnogdb/eggnog-mapper/issues/177).
And also from HMMER search options in eggNOG-mapper’s GitHub page here (https://github.com/eggnogdb/eggnog-mapper/wiki/eggNOG-mapper-v2-*refactor*#HMMer_search_options):
“requires -i FILE. Currently, it is only available for custom databases, and it does not provide annotations. see hmm_mapp
documentation.”
As can be read, HMMER is only allowed for custom databases but does not provide annotations (which somewhat defeats t
whole point). Since this may change in the future, we changed the sentence to what the reviewer suggested.

R2C41:
"Mantis ran with the following command: python mantis run_mantis -t sample.faa -od "NCBI ID" eggNOG-mapper was exe
with the following command: python emapper.py -i sample.faa -o output_folder -m diamond" (Methods - Establishing a te
environment)
According to this, the comparison is performed between Mantis using -od but eggNOG-mapper without taxonomy paramet
results to be comparable, both Mantis and eggNOG-mapper should be run with (-od for Mantis, --tax_scope for eggNOG-m
and without taxonomy.
Response:
We first ran eggNOG-mapper using target_taxa (which did not work as expected, so we removed it), the tax_scope was a
version v2.0.2-rf1 (which did not exist when we first ran the benchmark). We reran eggNOG-mapper with and without tax
Each sample was annotated by calculating its respective taxonomic lineage (e.g. Escherichia coli would run with “--tax_sco
561,1236,1224”), and adding it to the tax_scope during eggNOG-mapper’s execution.

R2C42:
Also regarding comparison of tools, I don't understand why comparison with other tools have not been performed, or thos
already done (included in supp xlsx) are not included in the main text.
Response:
We initially chose only to includebenchmarking against eggNOG-mapper as it is the most similar tool to Mantis. As sugges

the reviewer, we have now also added Prokka.
However it is to note that while Prokka is customizable, natively it includes a much smaller amount of data than Mantis (reformatting all the reference data used by Mantis so that it would be compatible with Prokka would be outside of the sco this manuscript). It also mostly attempts to annotate metabolism-related proteins (e.g., output has one column just for E numbers). Despite this, since Prokka is widely used, we believe it is still important to include it, if only as a point of refere future readers.
Initially we also benchmarked against RAST and deepEC, however we chose not to include these in the main text for differ reasons:

• DeepEC, this tool uses a pre-trained deep learning model which aims at annotating protein sequences with EC numbers. lack of customization and the fact that it was pre-trained does not allow for a fair comparison against Mantis, which uses a extensive amount of HMMs. Other non-reference based tools were not used for benchmarking for similar reasons.
• RAST uses TSHMMs (like eggNOG-mapper and Mantis), unfortunately we could not successfully use it in a local environn therefore high-throughput testing was not feasible. RAST, like Prokka, does its own gene-calling, however, unlike Prokka, significant amount of RAST's gene called sequences did not align to the reference sequences used during the benchmark.

Benchmarking against interproscan was not performed because this tool does not output one annotation per protein seque this regard, since interproscan and Mantis can be customized to use the same data, the main difference would fall in how analysed, Mantis attempts to provide the "best annotation", whereas interproscan provides all annotations. This does not an objective benchmark. Overall, the current functional annotation paradigm is to create high-quality reference datasets, then used to discover novel functions or to functionally characterize samples. Mantis does not attempt to provide novelty i manner, instead its goal is to be adaptable towards different goals whilst making the "best" use of the available data.

R2C43:
Also, running Mantis through the CAFA benchmark would be highly recommended (https://genomebiology.biomedcentral.com/articles/10.1186/s13059-019-1835-8)
Response:
Thank you for the suggestion, however, we opted not to perform this benchmark for several reasons:

1. GO terms centric benchmark : one of the reasons Mantis uses multiple reference data sources is to improve cross-linkin posterior data analysis, benchmarking only against GO terms does not capture this feature.
2. GO terms output broadness: Mantis and eggNOG-mapper both use the eggNOG reference data source. This reference c extensive cross-linking, however, for some "network-based" IDs (e.g., GO), it has been designed so that each functional annotation contains a broad list of related IDs. While this proves advantageous in annotation completeness, it also comes downside of not allowing for a proper benchmark of these IDs (i.e., GO terms) against a reference annotation. For exampl should a functional annotation contain the ID GO:0000451 (rRNA 2'-O-methylation), it will also contain the full list of relat terms, including very broad GO terms (e.g., GO:0009987 Cellular process). Therefore, if a comparison is done between a annotation and a reference annotation that happens to be linked to a cellular process (which is very likely), then this woul at least in our benchmark, in a TP, when in fact it the two annotations could be referring to a different function altogether Unfortunately, finding the leaf/terminal node in the gene ontologies network is not trivial (since it is not a truly hierarchica network). For the reasons above, during benchmark, we limit the amount of IDs of the same type (limit of 10 - empirically determined; please see quality_benchmark.py in the supplements), which in many instances excludes GO terms, instead benchmarking with the remaining types of IDs (e.g. Pfam).
3. Reference based approach - from our understanding CAFA is targeted towards machine learning approaches. While it is feasible to benchmark with CAFA, since Mantis is reference-based this dataset is not very relevant.
4. CAFA is a subset of Swiss-Prot - the main reason we decided not to use it was because CAFA is a selection of Swiss-Pro entries. Mantis was already benchmarked against a much more extensive list of Swiss-Prot entries, therefore while CAFA i benchmark, we did not find it as comprehensive as our own benchmark.

R2C44:
Finally, in my opinion precision (TP / TP + FP) is only part of the story, and besides "Annotation coverage", other metrics s be provided, specially some considering FNs (Sensitivity), which is the other part of the story I guess.
Response:
We have now included two other metrics in the manuscript, specifically recall/sensitivity and F1 score (harmonic mean of and precision). Indeed the F1 score is now used as a performance metric throughout the manuscript (the previous manus used precision). In addition, the supplements also contain all the confusion matrix classes, alongside other confusion matr derived metrics.

R2C45:
Finally, just 2 comments about the GitHub. I would be grateful if the web will point out that NOGT and NOGG are from eg for example here: "NOGT is the collection of taxon specific HMMs, NOGG the collection of all HMMs."
Response:
This has now been rectified in the GitHub page.

R2C46:
Also, I noticed that when using the "eggnog.db" sqlite3 file an index is created for the "name" field. I am not sure this is necessary, since "name" it is already a primary key in the "eggnog" table. I am not an expert on sqlite3 databases though[illegible]
Response:
The reviewer is correct, we removed this line from the code.

Close
