# Supplementary material for: Mantis: flexible and consensus-driven genome annotation
Source: Gigascience. 2021 Jun 2;10(6):giab042. doi: 10.1093/gigascience/giab042 (PMC8170692; doi:10.1093/gigascience/giab042)
Supplement: giab042_Response_to_Reviewer_Comments_Revision_1 [file giab042_response_to_reviewer_comments_revision_1.pdf]

Author's Response To Reviewer Comments

Close

We would like to thank the editor and reviewers for the new round of revision, we are overjoyed that our revised manuscript successfully addressed many of the concerns previously raised. We have organized this document in the same fashion, where each comment is marked as “RXCY”, X being the reviewer number and Y the comment number. R0 corresponds to the editor, R1 to the reviewer Karen Ross and R2 to the reviewer Carlos Cantalapiedra.
In response to the reviewer comments, we have updated some figures and slightly modified the manuscript. All modifications are described in detail in this document.

R0
R0C1:
the e-value: its precise meaning and how users handle this with their data
Response:
We thank the editor for this important comment. We have addressed this in the reviewer comments R1C2 and R2C6 and modified the manuscript accordingly.

R0C2:
impact of the tool for redundancy removal and over-annotation avoidance.
Response:
We have edited the manuscript to address the comment R2C4.

R0C3:
more details on the benchmarks
Response:
We have addressed the concerns that were raised in the comments R2C1 and R2C4.

R1

R1C,3,4,9:
Abstract: meaning of "high-result ion" data is unclear
Analysis: "(iii) how each reference data source's contribution to the final output"--change to "how each reference data source contributed to the final output"
Analysis: "(iv) impact of the consensus generation on annotations quality."-- change "annotations" to "annotation"
In the "Using multiple reference datasources" section, should the 1 in "e.g., if a hit comes from Pfam, that has a weight of 1, and another from eggNOG, that has a weight of 0.8, HMMW would equal to 0.9+0.8 = 0.85)" be "0.9"?
Response:
Thank you for these suggestions, we have amended the manuscript accordingly. Specifically, regarding the HMM weight, the reviewer is correct, since in our example the Pfam has a weight of 1 and eggNOG of 0.8 then the average should be 0.9 (i.e., changed to “(1+0.8)/2 = 0.9”).

R1C2:
Introduction: "depending on how strict the confidence threshold is, it may also increase the false-positives (over-annotation, due to a high confidence threshold) or false- negatives (under-annotation, due to a low confidence threshold)"--Is this backwards? Is seems to be that a high confidence threshold would accept only the most confident predictions and therefore lead to false negatives and vice versa.
Response:
We thank the reviewer for this question. Since the confidence threshold used throughout the manuscript corresponds to an e-value threshold, a higher threshold (e.g. 1e-3) would indeed produce more false positives, whereas a lower threshold (e.g. 1e-6) would produce more false-negatives. This is of course due to the inverse nature of the e-value, i.e., the closer to 0 the better. However, we agree that this sentence (lines 72-79) might be somewhat confusing, and have therefore have changed it to:

The selection of reference HMMs is also critical, as PFA will ultimately be based on the available reference data. Whilst using unspecific HMMs to annotate a taxonomically classified sample may result in a fair amount of true-positives (correct annotations), depending on the confidence threshold used, it may also increase the rate of false-positives (over-annotation, due to a less strict confidence threshold) or false-negatives (under-annotation, due to a more strict confidence threshold).

R1C5:
5. Figure 3: The difference between heuristic and DFS is very small and only visible with the 2020 sample, so the statement in the legend "The DFS algorithm outperforms the other algorithms." seems like a bit of an overstatement.
Response:
We agree with the reviewer, therefore we have changed the figure legend to:
"Overall, the DFS and heuristic algorithms achieve similar results, outperforming the BPO algorithm."

R1C6:
6. Figure 4: Sometimes the dot for one of the three methods is not visible (e.g., DFS dot in Cryptococcus without taxonomy). I assume this is because it is hidden by one of the other dots; maybe you could offset one of the dots above or below the other so that it is visible?
Response:
Thank you for the suggestion, we added a slight offset to the dark grey and yellow circles.

R1C7:
7. Figure 5: Should the scale bar for the F1 score be spanning the radius of the circle? Currently, it does not.
Response:
We have rechecked the image, and, unless we misunderstand what the reviewer means, the F1 score scale seems to span the radius. Please see the image below where we highlight that particular section of the image (we drew a square where the upper left vertice is fixed on the circle's center and the bottom right vertice is fixed on the F1 score scale number 1).

R1C8:
8. Computational efficiency: Why were pseudo-random sequences used for the efficiency test?
Response:
Since the reference datasets likely contain UniProt sequences (which were used for the computational efficiency test) we used pseudo-random sequences in order to add a degree of randomness to the homology search. However, we do agree this might not be necessary, therefore we have now removed it from the manuscript (edited lines 437-445).

R2

R2C1:
I think that the authors would improve the manuscript by reorganizing the Methods subsections related to sample selection and benchmarking, but just to facilitate readers interested in understanding better these specific aspects of the manuscript.
Response:
We thank the reviewer for the suggestion, we have moved the section "Sample selection" to before the section "Establishing a test environment" (previously it was after).

R2C2:
I just regret that you didn't agree with allowing using CDS as input, since it would be very useful for end users. Note that translating a CDS does not need any third party tool, but just a translation table (or using Biopython, which could be considered as using another third party package? though of broad use in python bioinformatics). I respect the author's decision though.
Response:
We thank the reviewer for this suggestion. In the previous revision we misunderstood the reviewer's suggestion. We are currently implementing other enhancements, but we have added this on to the to-do list for a next version.

R2C3:
Regarding reproducibility, I still disagree with the notion of "reproducible" used in the paper (e.g.: "a versalite and reproducible tool" (lines 204-205). How can a tool be reproducible?). Actually, the paper from Mangul is about "requirements to promote installability and long-term archival stability of software tools" which is not a feature of Mantis, but related to the requirements that have been asked by the editor (as you mention in the Discussion, with better criteria than in the introduction, in my opinion). Also, Snakemake, (Galaxy, other tools) may facilitate reproducibility, as a conda environment or even git versioning could do, for example. I am not saying reproducibility is not an important topic. I just don't like the use of the term in the manuscript. But I of course respect the author's point of view.
Response:
We agree with the reviewer that there is an ongoing discussion whether a tool can be reproducible and we respect the reviewer’s point of view, but will stay with the current wording.

R2C4:
Regarding over-annotation, I have yet doubts about whether you are really showing in the paper that this is true, or to what extent. In your benchmarks, you are measuring whether there is intersection of a single ID with the references, not whether the absent IDs are TNs or FNs due to the consensus you carry out. As such, how can you be sure that "over-annotation is minimized" and that "these three annotations are more likely to be valid" (line 186), and not just that these 3 are valid but the other 2 are also good (e.g. ID3 and ID4 in Figure 7). Avoiding over-annotation would be true only when you are correctly dealing with FPs being classified as TNs? I agree about "This is clearly a problem" and that consensus generation could be of help, but not sure to what extent Mantis does "addresses" this "very relevant" issue (lines 176-177) . Maybe some results regarding Precision with and without consensus, something similar, could help. Could you provide some evidence about this
among the results or in the discussion?
Response:
We thank the reviewer for this remark. We do provide results with and without consensus in the section “Impact of consensus generation” (second paragraph) and later provide a brief discussion on the benefit of using a consensus generation method; however, this does not directly address the reviewer’s concern. Our approach to dealing with over-annotation is similar to a voting system, where the majority is more likely to be correct. As we have previously acknowledged, a benchmark for this cannot be easily implemented (for example due to the heterogeneity of the reference data and reference annotations), therefore we have instead chosen to rephrase the sentence that mentions over-annotation:
We have attempted to avoid over-annotation through the generation of a consensus-driven annotation, which identifies and merges annotations that are consistent (i.e., similar function) with each other (e.g., if three out of five independent sources point towards the same function and two others point towards other, unrelated functions, then these three annotations are more likely to be valid), and eliminating the remaining inconsistent annotations.

We have also moved the paragraph explaining how we address under-annotation, over-annotation, and redundancy to the discussion (now in lines 464-479).

R2C5:
Also, after better understanding the use of free-text descriptions ("Similar functional descriptions, unless exactly the same, are kept regardless of their text similarity (e.g. "glucose degradation" and "degrades glucose"), in your answers", I disagree that "Redudancy is eliminated by removing duplicate database IDs and/or extremely similar descriptions" (line 187-189). I think it would be more accurate to say something like "Redundancy, which is a drawback inherent to consensus-driven annotation, is ameliorated by removing duplicate database IDs and/or identical descriptions."
Response:
Thank you for the suggestion, we agree with the suggestion, therefore the sentence was replaced (lines 469-471).

R2C6:
Regarding the e-value, I would really appreciate the authors to make clear the adjustment depending on sample size, since this has an impact on users results and reproducibility. As you describe it in your answers, is the e-value going to depend on the dinamically generated chunks of the input sequences? (in this sense, does the e-value represent the odds to find false positives in the HMM references, which

are the actual databases where the query is searched?). Also, the same query used as input along different queries (for example, a file with 10 sequences and a file with 1000 sequences, using different CPUs, or different distribution in a cluster) would lead to different e-values? Maybe this could be clarified in the "Multiple hits per protein" section, unless I am mistaken, in which case I would thank very much further clarification by the authors.
Response:
Yes, the raw e-value will depend on the dynamically generated chunk size of the input sequences, however, during processing, it is scaled to the original sequence size. As suggested by the reviewer, we added this sentence to the section "Accessibility and Scaling" (since it is here that we refer to sample splitting):
To note that Mantis uses HMMER's hmmsearch for homology search, which outputs an e-value scaled to the sample/chunk size. Since Mantis splits the samples into chunks, during hit processing, the e-value is scaled to the original sample size.

R2C7:
Abstract: What does "high-result ion" mean? Should it be "high-resolution"?
Response:
Thank you for this note, we have corrected it.

Close
