# Supplementary material for: Mantis: flexible and consensus-driven genome annotation
Source: Gigascience. 2021 Jun 2;10(6):giab042. doi: 10.1093/gigascience/giab042 (PMC8170692; doi:10.1093/gigascience/giab042)
Supplement: giab042_Response_to_Reviewer_Comments_Revision_2 [file giab042_response_to_reviewer_comments_revision_2.pdf]

Author's Response To Reviewer Comments

Close

Dear Editor,

We have updated the manuscript:
(i) added GigaScienceDB citation
(ii) added ORCIDs for all authors

Close
