# Supplementary material for: Mantis: flexible and consensus-driven genome annotation
Source: Gigascience. 2021 Jun 2;10(6):giab042. doi: 10.1093/gigascience/giab042 (PMC8170692; doi:10.1093/gigascience/giab042)
Supplement: giab042_Reviewer_1_Report_Original_Submission — Karen Ross -- 12/1/2020 Reviewed [file giab042_reviewer_1_report_original_submission.pdf]

Reviewer Report

Title: Mantis: flexible and consensus-driven genome annotation

Version: Original Submission **Date:** 12/1/2020

Reviewer name: Karen Ross

Reviewer Comments to Author:

This manuscript describes Mantis, a tool for protein function annotation based on sequence homology. There is a great need for high quality protein annotation tools due to the rapidly increasing amount of genome sequencing information that is available. Through its use of a depth first search algorithm and integration of multiple data resources, this tool has the potential to provide richer, more comprehensive protein annotations. However, the presentation of the method and its testing needs clarification to enable understanding of exactly how the tool works and to properly evaluate its performance. Providing specific examples in a few key places would help a lot.

1. Figures 5 and 6 make it clear that Mantis can find matches to multiple non-overlapping regions of the protein (multiple domain matches). It seems plausible that sometimes Mantis would identify some but not all of the domains in a manually annotated SwissProt entry or might identify some domains correctly but then also have one or more false positive domains. However, the calculation of %TP and %FP appears to be done at the whole protein level--"The % TPs and % FPs are, respectively, defined as the number of TPs and FPs divided by the number of sequences in the sample." How does this calculation take into account multiple matches per protein? An example here would help.
2. When creating the consensus annotation, Mantis evaluates whether the annotations from two different resources are similar. First, it is confusing that the authors use the word "complementary" to describe the situation where annotations from two resources are similar. "Consistent" or "confirmatory" might be better word choices. Second, it is unclear what the outcome would be if two resources find hits on two non-overlapping regions. These hits would obvious be very dissimilar in annotation. Would they both be included in the consensus annotation?
3. In the "Establishing a test environment" sub-section of the Methods, it would be helpful to clarify what is meant by "reference annotation". Where does it come from and what exactly does it consist of? Also, this section says that Mantis annotations that don't match the reference are considered false positives, but it also says that when Mantis outputs "valid annotation but the reference is either of poor quality or non-existent" that this is considered a potential new annotation. How exactly is the distinction made between a false positive and potential new annotation?
4. When calculating the similarity threshold (supplemental material), the authors analyzed a group of proteins where the identifiers disagreed. Were these cases where the Mantis annotation of a protein sequence had no identifiers in common with the Swiss-Prot annotation of the same sequence? Or was the Mantis-annotated sequence compared with a different sequence in Swiss-Prot that had no identifiers in common? If the sequences were the same, then why did they have no identifiers in common? A specific example would be helpful here.
5. How much does the text mining analysis of the free text descriptions contribute to the similarity

score? Is the similarity dominated by identifier matching or does the free text analysis contribute significantly?
6. Although this information is in the Methods, it would be helpful to define briefly in the Results section how precision is being calculated. It would be useful to include the explanation of why precision is considered more important than recall in the Results as well.
7. It would be useful to move figure 5 earlier in the paper when the hit processing algorithms are first introduced.
8. In the Background section, annotation iii could be removed from the first two examples of glucose degradation annotation because it isn't used to make the authors' point.

Level of Interest

Please indicate how interesting you found the manuscript: Choose an item.

Quality of Written English

Please indicate the quality of language in the manuscript: Choose an item.

Declaration of Competing Interests

Please complete a declaration of competing interests, considering the following questions:

- Have you in the past five years received reimbursements, fees, funding, or salary from an organisation that may in any way gain or lose financially from the publication of this manuscript, either now or in the future?
- Do you hold any stocks or shares in an organisation that may in any way gain or lose financially from the publication of this manuscript, either now or in the future?
- Do you hold or are you currently applying for any patents relating to the content of the manuscript?
- Have you received reimbursements, fees, funding, or salary from an organization that holds or has applied for patents relating to the content of the manuscript?
- Do you have any other financial competing interests?
- Do you have any non-financial competing interests in relation to this paper?

If you can answer no to all of the above, write 'I declare that I have no competing interests' below. If your reply is yes to any, please give details below.

I declare that I have no competing interests.

I agree to the open peer review policy of the journal. I understand that my name will be included on my report to the authors and, if the manuscript is accepted for publication, my named report including any attachments I upload will be posted on the website along with the authors' responses. I agree for my

report to be made available under an Open Access Creative Commons CC-BY license (http://creativecommons.org/licenses/by/4.0/). I understand that any comments which I do not wish to be included in my named report can be included as confidential comments to the editors, which will not be published.

Choose an item.

To further support our reviewers, we have joined with Publons, where you can gain additional credit to further highlight your hard work (see: https://publons.com/journal/530/gigascience). On publication of this paper, your review will be automatically added to Publons, you can then choose whether or not to claim your Publons credit. I understand this statement.

Yes Choose an item.
