# Supplementary material for: Mantis: flexible and consensus-driven genome annotation
Source: Gigascience. 2021 Jun 2;10(6):giab042. doi: 10.1093/gigascience/giab042 (PMC8170692; doi:10.1093/gigascience/giab042)
Supplement: giab042_Reviewer_1_Report_Revision_1 — Karen Ross -- 4/6/2021 Reviewed [file giab042_reviewer_1_report_revision_1.pdf]

Reviewer Report

Title: Mantis: flexible and consensus-driven genome annotation

Version: Revision 1 **Date:** 4/6/2021

Reviewer name: Karen Ross

Reviewer Comments to Author:

The manuscript is much improved. My concerns have been addressed, and I think the manuscript is suitable for publication. Below are a few minor suggestions to improve the presentation:
1. Abstract: meaning of "high-result ion" data is unclear
2. Introduction: "depending on how strict the confidence threshold is, it may also increase the false-positives (over-annotation, due to a high confidence threshold) or false- negatives (under-annotation, due to a low confidence threshold)"--Is this backwards? Is seems to be that a high confidence threshold would accept only the most confident predictions and therefore lead to false negatives and vice versa.
3.Analysis: "(iii) how each reference data source's contribution to the final output"--change to "how each reference data source contributed to the final output"
4. Analysis: "(iv) impact of the consensus generation on annotations quality."-- change "annotations" to "annotation"
5. Figure 3: The difference between heuristic and DFS is very small and only visible with the 2020 sample, so the statement in the legend "The DFS algorithm outperforms the other algorithms." seems like a bit of an overstatement.
6. Figure 4: Sometimes the dot for one of the three methods is not visible (e.g., DFS dot in Cryptococcus without taxonomy). I assume this is because it is hidden by one of the other dots; maybe you could offset one of the dots above or below the other so that it is visible?
7. Figure 5: Should the scale bar for the F1 score be spanning the radius of the circle? Currently, it does not.
8. Computational efficiency: Why were pseudo-random sequences used for the efficiency test?
9. In the "Using multiple reference datasources" section, should the 1 in "e.g., if a hit comes from Pfam, that has a weight of 1, and another from eggNOG, that has a weight of 0.8, HMMW would equal to 0.9+0.8 = 0.85)" be "0.9"?

Level of Interest

Please indicate how interesting you found the manuscript: Choose an item.

Quality of Written English

Please indicate the quality of language in the manuscript: Choose an item.

Declaration of Competing Interests

Please complete a declaration of competing interests, considering the following questions:

- Have you in the past five years received reimbursements, fees, funding, or salary from an organisation that may in any way gain or lose financially from the publication of this manuscript, either now or in the future?
- Do you hold any stocks or shares in an organisation that may in any way gain or lose financially from the publication of this manuscript, either now or in the future?
- Do you hold or are you currently applying for any patents relating to the content of the manuscript?
- Have you received reimbursements, fees, funding, or salary from an organization that holds or has applied for patents relating to the content of the manuscript?
- Do you have any other financial competing interests?
- Do you have any non-financial competing interests in relation to this paper?

If you can answer no to all of the above, write 'I declare that I have no competing interests' below. If your reply is yes to any, please give details below.

I declare that I have not competing interests.

I agree to the open peer review policy of the journal. I understand that my name will be included on my report to the authors and, if the manuscript is accepted for publication, my named report including any attachments I upload will be posted on the website along with the authors' responses. I agree for my report to be made available under an Open Access Creative Commons CC-BY license (http://creativecommons.org/licenses/by/4.0/). I understand that any comments which I do not wish to be included in my named report can be included as confidential comments to the editors, which will not be published.

Choose an item.

To further support our reviewers, we have joined with Publons, where you can gain additional credit to further highlight your hard work (see: https://publons.com/journal/530/gigascience). On publication of this paper, your review will be automatically added to Publons, you can then choose whether or not to claim your Publons credit. I understand this statement.

Yes Choose an item.
