# Supplementary material for: Mantis: flexible and consensus-driven genome annotation
Source: Gigascience. 2021 Jun 2;10(6):giab042. doi: 10.1093/gigascience/giab042 (PMC8170692; doi:10.1093/gigascience/giab042)
Supplement: giab042_Reviewer_2_Report_Original_Submission — Carlos Cantalapiedra -- 12/20/2020 Reviewed [file giab042_reviewer_2_report_original_submission.pdf]

Reviewer Report

Title: Mantis: flexible and consensus-driven genome annotation

Version: Original Submission **Date:** 12/20/2020

Reviewer name: Carlos Cantalapiedra

Reviewer Comments to Author:

Dear authors,
"Mantis: flexible and consensus-driven genome annotation" is a manuscript presenting novel software for computational functional annotation of proteins called Mantis. The software is presented as flexible, adaptable, reproducible, fast and scalable to metagenomes. The main features described in the manuscript are the use of a Depth-First Search algorithm to search for the best combination of hits from which to transfer annotations to a given protein query, the combination of unspecific and taxa-specific HMMs along with other HMM references (PFAM, KOFAM, TIGRFAM), and the consensus-driven integration of annotations from the different resources, which can be easily customized.
At first sight, it could be seen as a lighter, more easily customizable version of eggNOG-mapper. However, there are substantial differences which makes Mantis a very interesting software and a good contribution to the repertoire of functional annotation software. After testing Mantis, I would say that the main advantage I have found is that it is fast and it is rather easy to setup and customize.
The manuscript itself focuses on i) describing how Mantis works, ii) what are the advantages of the DFS algorithm, iii) what are the advantages of the use of taxa-specific HMMs, iv) the comparison of using different HMM DBs and with eggNOG-mapper.
In general, in my opinion, the manuscript is successful in some aspects: i) showing the advantages of using the DFS algorithm, ii) showing the advantages of using TSHMMs, iii) showing Mantis as a easy to customize software and flexible regarding the use of different databases.
There are other aspects that I personally would need more explanation to understand: i) what do the authors mean with adaptable, if it is different than flexible, ii) what do the authors mean with reproducible (which in general I would say it is considered implicit for most computational tools of this kind), iii) how several steps of the Mantis annotation algorithm actual work (specially those related with inter-HMM and consensus-driven integration), iv) how it actually scales from genomes to metagenomes, v) how it actually compares to other tools.
Also, there are some other more technical aspects that I consider that should be addressed before recommending the manuscript for publication.
Next, I will try to ellaborate in more detail my concerns with some of the points mentioned above.
I will start with some very minor points that the authors should review, but which should be completely solved before publication.
First, the list of keywords maybe should be reviewed: function as a keyword it is too generic in my opinion. protein; function; annotation could be merged into protein function annotation. Also, I could not find an explanation of what NLP means. Please, review also the other keywords included. The same for the list of abbreviations. For example, I could not find NLP there, but I found that BPO is also not

included. Please, review all abbreviations both from the main text and from the list.
Please, review also the text for potential errors. For example, "whilst sing" --> "whilst using", or "herein after" --> "hereinafter". I am also not native English, so my review in this sense could be not the best, so maybe a professional language review could be good to improve the manuscript. I leave this decision to the authors, editor and maybe some Ensligh-native speaker from among the reviewers.
Regarding supplemental material, it has been a bit confusing for me. There are links throughout the text, but there are also other supplemental files at the end of the manuscript. Are all of them valid?
Nomenclature of sections, tables, figures, etc from the different materials should be merged, and all supplemental material provided in a common way. This is what I had:
Throughout the text
Supplemental Table 1 is a link to
https://htmlpreview.github.io/?https://raw.githubusercontent.com/wiki/PedroMTQ/mantis/Resources/tab_e_value.html
Supplemental Table 2 is a link to
https://htmlpreview.github.io/?https://raw.githubusercontent.com/wiki/PedroMTQ/mantis/Resources/tab_uniprot_algorithms.html
Supplemental Table 3 is a link to
https://htmlpreview.github.io/?https://raw.githubusercontent.com/wiki/PedroMTQ/mantis/Resources/tab_genomes.html
At the end of the manuscript
Supplements.pdf
Defining a similarity threshold (Figure 1)
Annotating metagenomes
0.1 Benchmarking annotation efficiency (Figure 2, Table 1, Table 2)
Supplementary benchmarking against other PFA tools (Table 3, Table 4)
Supplemental tables (Table 5, Table 6, Table 7, Table 8)
References
Supplements.xlsx
Mantis vs emapper
DFS vs BPO vs heuristic
Comparisons with Prokka, DeepEC, emapper, RAST
Some comments about the abstract. What do the authors mean with "adaptable"? How is this different from "flexible"? Also, I would say that "reproducible" is implicit of computational tools, in general terms. Why are the authors stressing this in the abstract as a feature specifically of Mantis? Also, the authors say that "Mantis is fast, annotating an average genome in 25-40 minutes", which I would like better "an average bacterial genome", for instance.
And some comments about the Background section. I personally would remove the first 2 sentences, but this is completely personal preference. Also, I am not sure to agree with the protein function annotation definition which is given both in the abstract and in Background: "protein function annotation(PFA), which is the identification of regions of interest (domains) in a sequence and assignment of biological function(s) to these regions."
Later, the authors say that: "We reviewed the implementation of three widely used PFA tools [13, 24,

14] and observed that the processing of candidate annotations (i.e. sequences or HMM profiles which are highly similar to the query sequence) is done by capturing only the most significant candidate between the references ("best prediction only", herein after called BPO)". That is: Prokka, eggNOG-mapper and InterProScan 5. And, "This classic PFA approach works well for single-domain proteins, but multi-domain proteins may have multiple putative predictions [30, 31, 32], whose location in the sequence may or may not overlap.". The previous 2 sentences, although are true to some extent, are in my opinion an oversimplification. For example, stating that eggNOG-mapper picks the most significant candidate between the references is true, but only part of the story. eggNOG-mapper picks the most significant homolog as seed to retrieve orthologs from which annotations are transferred. Therefore, a protein can be annotated with multiple domains which may or may not overlap, which I believe is true also for Prokka and InterProScan 5. Therefore, although I think that the authors have a valid point here, I believe that they should focus on the advantages of identification of multiple references, but not as a mandatory requirement for multi-domain annotation. Actually, the 3 "reviewed" tools are very different, and if they are mentioned specifically as reviewed, a more precise description of their approaches, weaknesses or features which could be improved would be expected.

Then, regarding consensus integration of annotations the authors say that "This approach addresses three very relevant issues with PFA [34, 35, 51, 52]: over-annotation (through the use of overlapping but independent sources, thus obtaining a more reliable final annotation); under-annotation (through the use of multiple reference sources, which implicitly leads to a wider search space); and elimination of redundancy (through the creation of a consensus-driven annotation)." I agree that this approach would deal with under-annotation and with elimination of redundancy, due to integration of annotations from different sources. However, I fail to understand how this approach helps with over-annotation.

I will next comment on the sections in which how Mantis works is described.

First, regarding reference data, the authors say that eggNOG OGs are from eggNOG 5. However, actually TSHMMs and annotations downloaded by Mantis correspond to eggNOG 5 data, but the unspecific HMMs are from eggNOG 4.5. Checking some lines of code:

```
./source/MANTIS_DB.py:       eggnog_downloads_page_hmm =
'http://eggnogdb.embl.de/download/eggnog_4.5/data/NOG/NOG.hmm.tar.gz'
./source/MANTIS_DB.py:       eggnog_downloads_page_annot =
'http://eggnogdb.embl.de/download/eggnog_4.5/data/NOG/NOG.annotations.tsv.gz'
./source/MANTIS_DB.py:       eggnog_downloads_page =
'http://eggnog5.embl.de/download/latest/per_tax_level/'+str(taxon_id)+'/'
./source/MANTIS_DB.py:       url = 'http://eggnogdb.embl.de/download/emapperdb-5.0.0/eggnog.db.gz'
```

This is important, and it will impact both the use of the tool by users and the benchmarks presented in the paper. Note that eggNOG 4.5 and eggNOG 5 orthologous groups are not cross-linked, and therefore annotations for unspecific NOGs will be lacking in Mantis output. For example, I annotated with Mantis a protein using unspecific NOGs as ENOG4111x9y. This is an OG from eggNOG 4.5, as expected, and I got only the description as annotation ("uncharacterized protein yaho"), when I should be getting also annotation identifiers, GO terms for example. The equivalent OG in eggNOG 5 is ENOG502DN1T though. This can be checked comparing OGs from http://eggnog45.embl.de/ and http://eggnog5.embl.de/

Besides that, "TSHMMs metadata was extracted from the eggNOG SQL database" I would ask authors to specify that this is an eggNOG-mapper DB, not an eggNOG one, as can be seen in the link above.

Also, the TSHMMs databases are huge as a whole. In my opinion, including in Mantis an option to download only TSHMMs from a specific taxa would be great and almost mandatory for the "flexibility" that the authors have as a goal and claim in the manuscript (which in other aspects, like customization of DBs is achieved, in my opinion).

Mantis workflow is described as consisting of 6 steps:

i) sample pre-processing, where samples are split in chunks for parallelization. Also, input data should be proteins. Are CDS queries (fasta in nucleotide/DNA format) also accepted?

ii) HMM profile-based homolgy search against each reference dataset using HMMER (hmmsearch). It is a bottom-top hierarchical search for TSHMMs, which is great.

iii) intra-HMM reference hits processing.

"HMMER outputs a domtblout le [23], where each line corresponds to a hit/match between the reference dataset and the unknown protein sequence. The e-value within the HMMER command limits the available solution space to be analyzed in the posterior processing steps." Which is the e-value used by Mantis then? The e-value from "the HMMER command" or the e-value from the "domtblout" file? Note that e-value in "domtblout" is the "e-value of the overall sequence/profile comparison (including all domains)", which outputs also other values, like "c-evalue" and "i-evalue". (http://eddylab.org/software/hmmer/Userguide.pdf, pp70-71, see also pp 34-35). Therefore, if using the "e-value", either all the hits from such domain should be included for intra-HMM integration, or preferably some hits should be discarded using the "i-evalue", for example. Also note that as the score used to compare combinations through DFS includes e-value, if several repeated domains exist in a sequence maybe the i-evalue should be included in the score instead of the overall e-value several times, which would inflate the combination in which several domains are part of the sequence.

"should the DFS algorithm running time exceed 60 seconds, Mantis employs the previously described "heuristic" algorithm [30], which scales linearly and outputs an unique combination of hits." How many queries in average could be affected by this? Is the user wanred somehow if some results are produced with the heuristic algorithm due to this DFS time limit?

Are "output_annotation.tsv" files a product of this 3rd step?

iv) metadata integration. Annotation description and identifiers are added to the respective hits. I guess this yields the "integrated_annotation.tsv" output files.

v) inter-HMM reference hits processing.

I have many doubts with this step. First, in "Mantis" section it is said that "During inter-HMM hits processing the DFS algorithm is again used to generate all the combinations of hits for all HMM sources." However, in "Methods - Using multiple reference datasets" this is not confirmed not further explained. Also, if DFS is used here, how are the e-values from the different references used to compute the scores. Because e-values are depend on database size, in contrast with bit-scores. Also, query coverage will be very different by the very nature of the HMM profiles of the different databases. For example, PFAM is a database of domains, whereas eggNOG contains profiles which usually span a large section of the protein sequence.

Moreover, it said later that "Since several groups of consensus annotations may be generated, we evaluate their quality and select the best one, considering the following: percentage of the sequence covered by the hits in the consensus, the significance of the hits (e-value) in the consensus, significance of the reference datasets (customizable), and the number of different reference datasets in the

consensus."
"Since some sources are more specific than others, the user may also customize the weight given to each source during consensus generation [73]." (Methods - Reference data and customization)
Does this have to do with the DFS algorithm for inter-HMM integration? Also, how is the weighting of different references used? In the GitHub it is explained that it is a [0-1] value, but, is it just a priority value or is it included in the DFS scoring formula somehow? I fail to find an explanation about how this weighting is used at all.
vi) Consensus generation
From Background section: "However, to our knowledge, there is no tool for the dynamic generation of a consensus from multiple protein annotations." "We implemented a two-fold approach to build a consensus annotation, first by checking for any intersecting annotation identifiers and second by evaluating how similar the free-text annotation descriptions are." From the "Mantis" section: "all the combinations of hits are expanded and intersected (if possible), the best consensus combination of hits is then selected for each query sequence."
So the tool is "checking" and/or "evaluating" intersections of identifiers and similarity of descriptions. But I fail to understand what is the desired or expected output after the check is done. According to the text, one would expect to obtain the intersection of the identifiers, which actually does not make sense at all, since many identifiers would be discarded just because they are not part of one or the other reference. The same question regarding descriptions: several descriptions are compared in a pairwise manner and those pairs with similarity are kept and those without any similar description are discarded?
Again, in "Methods - using multiple reference datasets": "For the integration of multiple reference datasets, a two-fold text mining approach was used: 1. Consensus between identifiers; and 2. Consensus between the free-text annotation description." "The consensus between identifiers is calculated by identifying intersections between the different sources. Identifiers within the free-text annotation descriptions are extracted and used here."
And also, "If no consensus between identifiers is found, then we proceed with a consensus calculation between annotation descriptions." so I guess that similarity of descriptions is only evaluated when no intersection is found among all the annotation identifiers? If this is true, then multiple identical descriptions would be kept just because a single domain is common to both reference sources? My sincere apologies, but I am missing some key point for sure here. I would ask the authors to explain in more detail how this works.
Also, if I understood correctly, there can be several descriptions from a single source. For example, if the right combination of hits for a query are 2 different PFAM domains, with different descriptions. How is this handled? Are all descriptions compared in a pairwise manner intra and inter reference?
Regarding the similarity of descriptions, the authors say that "Alongside Mantis, We developed a standalone tool [58] that allows the use of multi-ple reference datasets through the generation of a consensus annotation. As we have shown in the supplement "Defining a similarity threshold", this tool has high specificity, thus, in the context of Mantis, it allows for the correct identification of similar free-text annotation descriptions."
I guess this "high specificity" and how the threshold is defined should be part of the main manuscript in the Methods section? Even if this is left in the supplemental, it should be explained how this "high specificity" is achieved, and also how the definition of 0.8 as threshold is not arbitrary, and it is not as

good as 0.6, 0.7 or 0.9. Also they say (in supplemental) that: "In the current scenario, sensitivity is unimportant for two reasons:
1. the reference datasets from Mantis may contain data from Swiss-Prot (TPs inflation)
2. two annotations may completely agree in identifiers but, while describing the same function, be lexically different (FNs inflation)"
which I fail to understand. They define "False-positives (FP) = similarity score is above the threshold and identifiers disagree", which they say in the introduction that it is the goal of the consensus-description annotation. Thus, if you define these as FPs, you are contradicting the goal you define in the introduction. Also, "False-negatives (FN) = similarity score is below the threshold and identifiers agree", even when FNs are not going to be processed if those annotations with common identifiers are not processed for consensus-description, it genuinely gives an idea of the FN rate you get, and thus the sensitivity of the method, which is relevant to assess the potential impact of the consensus-description method to expand the annotations from results without common identifiers.
Besides the above comments, I tried to understand how these v and vi steps work by looking at some examples.
Example 1: 5 descriptions from a query:
description:Response Regulator
description:Two-component systems (sub1role)
description:Signal transduction (mainrole)
description:Transcriptional regulator
description:two-component system, sensor histidine kinase ChiS
Shouldn't at least 2 of the descriptions be merged?
Example 2: 7 descriptions from a query
description:DNA metabolism (mainrole)
description:helicase
description:Restriction endonuclease
description:Type ISP C-terminal specificity domain
description:Restriction/modification (sub1role)
description:type iii restriction protein res subunit
description:Type III restriction enzyme, res subunit
Example 3: full annotation of a query
query_ID NOGG_merged;Pfam-A Plug;ENOG410XQM1 2 6 | pfam:PF07715
description:tonB-dependent Receptor Plug
description:TonB-dependent Receptor Plug Domain
Example 4: comparing hits with and without tax info (-od)
without -od
NP_416050.4 kofam_merged K15268 1 4 | cog:COG0697 kegg_ko:K15268
tcdb:2.A.7.3.2 description:O\-acetylserine/cysteine efflux transporter
with -od "Escherichia coli"
NP_416050.4 kofam_merged;NOGT561_merged K15268;3XP05 2 4 |
bigg_reaction:iECIAI39_1322.ECIAI39_1\835 cog:COG0697 go:0000101 go:0003333
go:0003674 go:0005215 go:0005575 go:0005623 go\:0005886 go:0006810 go:0006811

go:0006812 go:0006820 go:0006865 go:0008150 go:0015562 \ go:0015711 go:0015804 go:0015849 go:0016020 go:0016021 go:0022857 go:0031224 go:0032973 \ go:0033228 go:0034220 go:0042883 go:0044425 go:0044464 go:0046942 go:0051179 go:005123\4 go:0055085 go:0071702 go:0071705 go:0071944 go:0072348 go:0098655 go:0098656 go:014\0115 go:1903712 go:1903825 go:1905039 kegg_brite:ko00000 kegg_brite:ko02000 kegg_ko:K15268 tcd\b:2.A.7.3.2 description:O-acetylserine/cysteine efflux transporter description:May be an export pump for cysteine and other\ metabolites of the cysteine pathway (such as N-acetyl-L-serine (S) and O- acetyl-L-serine (OAS)), and for other amino acids \and their metabolites

In this case, the annotation with -od seems richer. Besides that, there is certain redundancy in identifiers (K15268) and possibly in descriptions also (cysteine transporter, export for cysteine).

Example 5: comparing hits with and without tax info (-od) (II)

without -od

NP_417950.1 kofam_merged;NOGG_merged COG0306;K16322 2 3 | enzyme_ec:3.4.14.10 enzyme_ec:5.4\
.2.12 bigg_reaction:iECABU_c1320.ECABU_c33930 bigg_reaction:iECO111_1330.ECO111_4301 bigg_reaction:iNJ661.Rv0545c bi\
gg_reaction:iNJ661.Rv2281 bigg_reaction:iPC815.YPO3967 cog:COG0306
… GO terms … KEGG brite terms … kegg_ko:K01280 kegg_ko:K03306 kegg_ko:K03569 kegg_ko:K04043 kegg_ko:K14640 kegg_ko:K15633 kegg_ko:K16322 kegg_ko:K16331 … KEGG modules, pathways, ...tcdb:2.A.20.1 description:inorganic phosphate transmembrane transporter activity \ description:low-affinity inorganic phosphate transporter

with -od "Escherichia coli"

NP_417950.1 kofam_merged;NOGT561_merged K16322;3XMW3 2 3 | bigg_reaction:iECO111_1330.ECO111_430\1 cog:COG0306 … GO terms ... kegg_brite:ko00000 \ kegg_brite:ko02000 kegg_ko:K16322 tcdb:2.A.20.1 description:low-affinity inorganic phosphate transporter de\scription:Low-affinity inorganic phosphate transport. Can also transport arsenate

In this case, the without -od annotation seems richier. Again, KEGG and descriptions consensus seem to be somewhat limited.

Example 6: inter-HMM integration

from "output_annotation.tsv"

YP_025301.1 Pfam-A HOK_GEF PF01848.17 5.93440414507772e-22 49 8 43 2 42
YP_025301.1 kofam_merged K18921 - 1.3407357512953368e-23 49 4 46 42 89
YP_025301.1 NOGG_merged NOG.ENOG410ZT31.meta_raw - 2.1979274611398962e-27 49 4 46 56 102

Here all the 3 hits overlap.

From "integrated_annotation.tsv"

Query HMM_file HMM_hit HMM_hit_ac evalue l qs qe ss se
YP_025301.1 Pfam-A HOK_GEF PF01848.17 5.93440414507772e-22 49 8 43 2 42 | pfam:PF01848 \ description:Hok/gef family
YP_025301.1 kofam_merged K18921 - 1.3407357512953368e-23 49 4 46 42 89 | kegg_ko:K18921 \ description:protein HokB

YP_025301.1 NOGG_merged ENOG410ZT31 - 2.1979274611398962e-27 49 4 46 56 102 | description:Hok/gef family

The final "consensus_annotation.tsv"

YP_025301.1 Pfam-A;NOGG_merged ENOG410ZT31;HOK_GEF 2 3 | pfam:PF01848 description:Hok/gef f\amily

Why is the Pfam included here if it does overlap with the NOGG result? Why not the kofam hit? Descriptions of PFAM and NOGG match, and the evalue is better for NOGG. I would expect either PFAM or NOGG to be discarded, and, just maybe, kofam included due to slightly different description?

Example 7: the same as above, but with -od "Escherichia coli"

From "integrated_annotation.tsv":

YP_025301.1 Pfam-A HOK_GEF PF01848.17 5.93440414507772e-22 49 8 43 2 42 | pfam:PF01848 d\escription:Hok/gef family

YP_025301.1 kofam_merged K18921 - 1.3407357512953368e-23 49 4 46 42 89 | kegg_ko:K18921 d\escription:protein HokB

YP_025301.1 NOGT561_merged 3XRAS - 1.4945906735751294e-15 49 6 46 7 49 | description:Hok/gef family

Final "consensus_annotation.tsv"

YP_025301.1 kofam_merged K18921 1 3 | kegg_ko:K18921 description:protein HokB

In this case only the kofam hit is kept (due to being the one with the best evalue?).

Example 8:

From "output_annotations.tsv"

1000565.METUNv1_03812 tigrfam_merged TIGR00092 TIGR00092 1.1e-161 363 20 \344 1 368

1000565.METUNv1_03812 kofam_merged K19788 - 2.7e-151 363 20 \344 79 446

1000565.METUNv1_03812 Pfam-A MMR_HSR1 PF01926.24 3.5e-26 363 13 \158 2 87

1000565.METUNv1_03812 Pfam-A YchF-GTPase_C PF06071.14 3.7e-42 363 284 \357 1 84

1000565.METUNv1_03812 NOGG_merged NOG.COG0012.meta_raw - 4.7e-144 363 20 \344 273 573

From "consensus_annotation.tsv"

1000565.METUNv1_03812 kofam_merged;tigrfam_merged;NOGG_merged COG0012;TIGR00092;K19788

The PFAM domains are discarded, whereas kofam and NOGG results are included despite overlapping with with TIGRFAM, which has the best e-value.

Example 9:

From "output_annotation.tsv"

362663.ECP_0061 kofam_merged K02336 - 0.0 783 41 \743 96 876

362663.ECP_0061 Pfam-A DNA_pol_B PF00136.22 5e-47 783 385 \744 22 414

362663.ECP_0061 NOGG_merged NOG.COG0417.clustalo_raw - 2.4e-127 783 70
\742 872 1449
362663.ECP_0061 Pfam-A DNA_pol_B_exo1 PF03104.20 4.6e-10 783 197
\290 241 337
362663.ECP_0061 tigrfam_merged TIGR00592 TIGR00592 8.4e-49 783 217
\736 597 1141
From "consensus_annotation.tsv"
362663.ECP_0061 kofam_merged;NOGG_merged K02336;COG0417
In this case TIGRFAM and PFAM are discarded, whereas NOGG is kept despite overlapping with kofam hit and having worse e-value.
Overall, I have problems understanding steps v and vi: inter-HMM integration and consensus generation. In my opinion, the explanations about how the algorithm works should be improved, made more accurate and exhaustive, at least in the Methods section. It could be needed to adjust some of the procedures/parameters to obtain more comprehensive and less redundant annotations, which should be one of the major advantages of Mantis according to the authors.
Now, I will comment regarding to the benchmarks presented in the manuscript.
In Methods - Accessibility and Scaling "Mantis is also scalable to metagenomes. For more details on performance see Annotating metagenomes in supplements." Also said in Abstract/Conclusions, at the end of the Background section
I think that if authors want to state that Mantis is scalable to metagenomes they should provide data supporting this in the main text. According to the results in the supplements, it is indeed scalable, in my opinion, although I ran a test with a sample with 100k queries and got rather slower results, but capable of running the analysis in a decent amount of time (120946 secs â†’ 33.60 hours) anyway.
Methods - Stablishing a test environment "True-Positives (TP) are evaluated via two main methods: i) identifiers match and ii) description match"
Each query can have multiple annotation items (identifiers and descriptions). Each item that matches with one of the reference is a new TP? Or a TP is the annotation of query as a whole? This should further explained.
The same for FP "Annotations which do not match with the reference annotation are classied as False-Positives (FP)."
Is it the whole annotation or each of the items (identifiers and descriptions) could be a FP or TP independently of the others?
How are FPs, TPs ... computed when benchmarking SwissProt proteins? What are the annotation identifiers used from SwissProt? Or is it only description used?
"Whenever Mantis outputs a valid annotation but the reference is either of poor quality or non-existent, then this annotation is considered a potentially new annotation." (Methods - Establishing a test environment)
I think this sentence is a bit obscure. What is a "valid annotation" from Mantis, in comparison with a reference which is of "poor quality or non-existent", and what does mean for FPs and TPs the consideration of "potentially new annotation"? Is it not considered as a TP, a FP, neither? Shouldn't references of poor quality or non-existent just be removed from the benchmark?
"Annotation coverage is defined here as the number of Mantis' annotations divided by the total amount

of protein sequences in a sample." (Methods - Establishing a test environment)
Is this the same as the % of queries which have received (consensus) annotation?
"As seen in supplemental Table 1, being more permissive (by using a higher e-value threshold e.g. 1e - 3) resulted in a higher annotation coverage and a higher precision." (Analysis - Initial quality control - Function assignment e-value threshold)
It is surprising that higher e-value threshold leads to higher precision: "This is due to Mantis' internal quality control in the form of the DFS hit-processing algorithm. Being too strict with the e-value threshold constricts the available solution space, resulting in a lower precision." However, expanding the solution space would yield higher TP rate, which potentially could lead to higher precision if FP rate does not increase so much as TP rate. However, in supplements.xlsx it is shown (e-value tab) that absolute number of FPs are reduced when using a higher e-value, and this is much more surprising. Although this could be true, the authors should check this result is an artifact of the definitions and methods used to compute TP/FPs should be discarded. If the authors really did, my apologies, and also maybe they could further explain why this happens.
Initial Quality Control "When comparing the hit processing algorithms we found that the DFS algorithm consistently outperformed the other algorithms, with an average precision 0.038 and 0.013 higher than the BPO and heuristic algorithms respectively."
Is this difference statistically significative?
"for homology search, Mantis uses HMMER [23] and eggNOG-mapper uses Diamond [22]." (Methods - Establishing a test environment)
This is not entirely true, since eggNOG-mapper can use both HMMER and/or Diamond, depending on the eggNOG-mapper version being used. I would rather prefer "and for eggNOG-mapper we used the Diamond-based search".
"Mantis ran with the following command: python mantis run_mantis -t sample.faa -od "NCBI ID" eggNOG-mapper was executed with the following command: python emapper.py -i sample.faa -o output_folder -m diamond" (Methods - Establishing a test environment)
According to this, the comparison is performed between Mantis using -od but eggNOG-mapper without taxonomy parameter. For results to be comparable, both Mantis and eggNOG-mapper should be run with (-od for Mantis, --tax_scope for eggNOG-mapper) and without taxonomy.
Also regarding comparison of tools, I don't understand why comparison with other tools have not been performed, or those already done (included in supp xlsx) are not included in the main text. Also, running Mantis through the CAFA benchmark would be highly recommended (https://genomebiology.biomedcentral.com/articles/10.1186/s13059-019-1835-8)
Finally, in my opinion precision (TP / TP + FP) is only part of the story, and besides "Annotation coverage", other metrics should be provided, specially some considering FNs (Sensitivity), which is the other part of the story I guess.
Finally, just 2 comments about the GitHub. I would be grateful if the web will point out that NOGT and NOGG are from eggNOG, for example here: "NOGT is the collection of taxon specific HMMs, NOGG the collection of all HMMs."
Also, I noticed that when using the "eggnog.db" sqlite3 file an index is created for the "name" field. I am not sure this is necessary, since "name" it is already a primary key in the "eggnog" table. I am not an expert on sqlite3 databases though.

I would like to thank the authors for developing this tool, and I would ask them sincerely to improve it, and also the manuscript, so that more people can take advantage of it.
Best

Level of Interest

Please indicate how interesting you found the manuscript: Choose an item.

Quality of Written English

Please indicate the quality of language in the manuscript: Choose an item.

Declaration of Competing Interests

Please complete a declaration of competing interests, considering the following questions:

- Have you in the past five years received reimbursements, fees, funding, or salary from an organisation that may in any way gain or lose financially from the publication of this manuscript, either now or in the future?
- Do you hold any stocks or shares in an organisation that may in any way gain or lose financially from the publication of this manuscript, either now or in the future?
- Do you hold or are you currently applying for any patents relating to the content of the manuscript?
- Have you received reimbursements, fees, funding, or salary from an organization that holds or has applied for patents relating to the content of the manuscript?
- Do you have any other financial competing interests?
- Do you have any non-financial competing interests in relation to this paper?

If you can answer no to all of the above, write 'I declare that I have no competing interests' below. If your reply is yes to any, please give details below.

6. I am member of the main development team of eggNOG-mapper, which could be considered direct competition for Mantis, and which is compared to it in the manuscript.

I agree to the open peer review policy of the journal. I understand that my name will be included on my report to the authors and, if the manuscript is accepted for publication, my named report including any attachments I upload will be posted on the website along with the authors' responses. I agree for my report to be made available under an Open Access Creative Commons CC-BY license (http://creativecommons.org/licenses/by/4.0/). I understand that any comments which I do not wish to

be included in my named report can be included as confidential comments to the editors, which will not be published.

Choose an item.

To further support our reviewers, we have joined with Publons, where you can gain additional credit to further highlight your hard work (see: https://publons.com/journal/530/gigascience). On publication of this paper, your review will be automatically added to Publons, you can then choose whether or not to claim your Publons credit. I understand this statement.

Yes Choose an item.
