# Supplementary material for: Mantis: flexible and consensus-driven genome annotation
Source: Gigascience. 2021 Jun 2;10(6):giab042. doi: 10.1093/gigascience/giab042 (PMC8170692; doi:10.1093/gigascience/giab042)
Supplement: giab042_Reviewer_2_Report_Revision_1 — Carlos Cantalapiedra -- 4/22/2021 Reviewed [file giab042_reviewer_2_report_revision_1.pdf]

Reviewer Report

Title: Mantis: flexible and consensus-driven genome annotation

Version: Revision 1 **Date:** 4/22/2021

Reviewer name: Carlos Cantalapiedra

Reviewer Comments to Author:

Dear authors,
First of all, I would like to thank you for improving the manuscript, for providing the means to facilitate our tasks as reviewers, including the reorganization of supplementary material, and for the patience when answering my questions and doubts.
I like the new additions, especially Figures 2 and 7. I believe that the Mantis algorithm can be understood better now, specially the inter-HMM integration and consensus steps, which I was struggling to understand in practice. I also thank for the many explanations regarding the use of HMMER e-values, the notes about impact of DFS time limit, and the description of the output files generated by Mantis. I hope that all this is of help for readers also. In this regard, I think that the authors would improve the manuscript by reorganizing the Methods subsections related to sample selection and benchmarking, but just to facilitate readers interested in understanding better these specific aspects of the manuscript.
I also believe that the statements about other PFA tools are more fair now. Also, thank you for updating the eggNOG general HMMs to use eggNOG 5, and for making more accurate the references to eggNOG and eggNOG-mapper resources. Also thank you for changing the sentence about eggNOG-mapper homology search. I just didn't want the reader to think that eggNOG-mapper is using only Diamond, because nowadays it is not true, although the authors are completely right that at the moment they asked we were only giving support for diamond search (for version 2). Note that hmm_mapper.py does not perform annotation, but emapper.py yes, it is now again able to use the hmmer mode, after downloading and building the corresponding eggNOG 5 databases, of course. We still recommend using Diamond in most cases, though. I guess changing directions is a normal thing during support and the cycle of development of any tool. I am surprised about the tax_scope though, since I thought it was present since version 1? Since you very kindly did the tax scope analysis I won't even check. I just think that your decision improves the comparison and the taxa-based annotation discussion.
I also think that including Prokka improves the interest of the results for the audience, due to the broad use of this tool for PFA (especially in prokaryotes). It is a shame that other tools and the CAFA benchmark did not make it, but I thank the author's explanations about why they are out.
I also thank you for acknowledging the proposed changes in the tool, which were made with the only aim to enhance Mantis flexibility for users. I just regret that you didn't agree with allowing using CDS as input, since it would be very useful for end users. Note that translating a CDS does not need any third party tool, but just a translation table (or using Biopython, which could be considered as using another third party package? though of broad use in python bioinformatics). I respect the author's decision though.
Regarding reproducibility, I still disagree with the notion of "reproducible" used in the paper (e.g.: "a

versalite and reproducible tool" (lines 204-205). How can a tool be reproducible?). Actually, the paper from Mangul is about "requirements to promote installability and long-term archival stability of software tools" which is not a feature of Mantis, but related to the requirements that have been asked by the editor (as you mention in the Discussion, with better criteria than in the introduction, in my opinion). Also, Snakemake, (Galaxy, other tools) may facilitate reproducibility, as a conda environment or even git versioning could do, for example. I am not saying reproducibility is not an important topic. I just don't like the use of the term in the manuscript. But I of course respect the author's point of view.
Regarding over-annotation, I have yet doubts about whether you are really showing in the paper that this is true, or to what extent. In your benchmarks, you are measuring whether there is intersection of a single ID with the references, not whether the absent IDs are TNs or FNs due to the consensus you carry out. As such, how can you be sure that "over-annotation is minimized" and that "these three annotations are more likely to be valid" (line 186), and not just that these 3 are valid but the other 2 are also good (e.g. ID3 and ID4 in Figure 7). Avoiding over-annotation would be true only when you are correctly dealing with FPs being classified as TNs? I agree about "This is clearly a problem" and that consensus generation could be of help, but not sure to what extent Mantis does "addresses" this "very relevant" issue (lines 176-177) . Maybe some results regarding Precision with and without consensus, something similar, could help. Could you provide some evidence about this among the results or in the discussion?
Also, after better understanding the use of free-text descriptions ("Similar functional descriptions, unless exactly the same, are kept regardless of their text similarity (e.g. "glucose degradation" and "degrades glucose"), in your answers", I disagree that "Redudancy is eliminated by removing duplicate database IDs and/or extremely similar descriptions" (line 187-189). I think it would be more accurate to say something like "Redundancy, which is a drawback inherent to consensus-driven annotation, is ameliorated by removing duplicate database IDs and/or identical descriptions."
Regarding the e-value, I would really appreciate the authors to make clear the adjustment depending on sample size, since this has an impact on users results and reproducibility. As you describe it in your answers, is the e-value going to depend on the dinamically generated chunks of the input sequences? (in this sense, does the e-value represent the odds to find false positives in the HMM references, which are the actual databases where the query is searched?). Also, the same query used as input along different queries (for example, a file with 10 sequences and a file with 1000 sequences, using different CPUs, or different distribution in a cluster) would lead to different e-values? Maybe this could be clarified in the "Multiple hits per protein" section, unless I am mistaken, in which case I would thank very much further clarification by the authors.
Minor things:
Abstract: What does "high-result ion" mean? Should it be "high-resolution"?

Level of Interest

Please indicate how interesting you found the manuscript: Choose an item.

Quality of Written English

Please indicate the quality of language in the manuscript: Choose an item.

Declaration of Competing Interests

Please complete a declaration of competing interests, considering the following questions:

- Have you in the past five years received reimbursements, fees, funding, or salary from an organisation that may in any way gain or lose financially from the publication of this manuscript, either now or in the future?
- Do you hold any stocks or shares in an organisation that may in any way gain or lose financially from the publication of this manuscript, either now or in the future?
- Do you hold or are you currently applying for any patents relating to the content of the manuscript?
- Have you received reimbursements, fees, funding, or salary from an organization that holds or has applied for patents relating to the content of the manuscript?
- Do you have any other financial competing interests?
- Do you have any non-financial competing interests in relation to this paper?

If you can answer no to all of the above, write 'I declare that I have no competing interests' below. If your reply is yes to any, please give details below.

6. I am developer of a competing tool (eggNOG-mapper)

I agree to the open peer review policy of the journal. I understand that my name will be included on my report to the authors and, if the manuscript is accepted for publication, my named report including any attachments I upload will be posted on the website along with the authors' responses. I agree for my report to be made available under an Open Access Creative Commons CC-BY license (http://creativecommons.org/licenses/by/4.0/). I understand that any comments which I do not wish to be included in my named report can be included as confidential comments to the editors, which will not be published.

Choose an item.

To further support our reviewers, we have joined with Publons, where you can gain additional credit to further highlight your hard work (see: https://publons.com/journal/530/gigascience). On publication of this paper, your review will be automatically added to Publons, you can then choose whether or not to claim your Publons credit. I understand this statement.

Yes Choose an item.
